# Modelling of Yeast Mating Reveals Robustness Strategies for Cell-Cell Interactions

**DOI:** 10.1371/journal.pcbi.1004988

**Published:** 2016-07-12

**Authors:** Weitao Chen, Qing Nie, Tau-Mu Yi, Ching-Shan Chou

**Affiliations:** 1 Department of Mathematics, University of California, Irvine, Irvine, California, United States of America; 2 Molecular, Cellular, and Developmental Biology, University of California, Santa Barbara, Santa Barbara, California, United States of America; 3 Department of Mathematics, The Ohio State University, Columbus, Ohio, United States of America; University of British Columbia, CANADA

## Abstract

Mating of budding yeast cells is a model system for studying cell-cell interactions. Haploid yeast cells secrete mating pheromones that are sensed by the partner which responds by growing a mating projection toward the source. The two projections meet and fuse to form the diploid. Successful mating relies on precise coordination of dynamic extracellular signals, signaling pathways, and cell shape changes in a noisy background. It remains elusive how cells mate accurately and efficiently in a natural multi-cell environment. Here we present the first stochastic model of multiple mating cells whose morphologies are driven by pheromone gradients and intracellular signals. Our novel computational framework encompassed a moving boundary method for modeling both **a**-cells and α-cells and their cell shape changes, the extracellular diffusion of mating pheromones dynamically coupled with cell polarization, and both external and internal noise. Quantification of mating efficiency was developed and tested for different model parameters. Computer simulations revealed important robustness strategies for mating in the presence of noise. These strategies included the polarized secretion of pheromone, the presence of the α-factor protease Bar1, and the regulation of sensing sensitivity; all were consistent with data in the literature. In addition, we investigated mating discrimination, the ability of an **a**-cell to distinguish between α-cells either making or not making α-factor, and mating competition, in which multiple **a**-cells compete to mate with one α-cell. Our simulations were consistent with previous experimental results. Moreover, we performed a combination of simulations and experiments to estimate the diffusion rate of the pheromone **a**-factor. In summary, we constructed a framework for simulating yeast mating with multiple cells in a noisy environment, and used this framework to reproduce mating behaviors and to identify strategies for robust cell-cell interactions.

## Author Summary

One of the riddles of Nature is how cells interact with one another to create complex cellular networks such as the neural networks in the brain. Forming precise connections between irregularly shaped cells is a challenge for biology. We developed computational methods for simulating these complex cell-cell interactions. We applied these methods to investigate yeast mating in which two yeast cells grow projections that meet and fuse guided by pheromone attractants. The simulations described molecules both inside and outside of the cell, and represented the continually changing shapes of the cells. We found that positioning the secretion and sensing of pheromones at the same location on the cell surface was important. Other key factors for robust mating included secreting a protein that removed excess pheromone from outside of the cell so that the signal would not be too strong. An important advance was being able to simulate as many as five cells in complex mating arrangements. Taken together we used our novel computational methods to describe in greater detail the yeast mating process, and more generally, interactions among cells changing their shapes in response to their neighbors.

## Introduction

Cell-to-cell signaling via diffusible molecules is an important mode of communication between cells in many mammalian systems such as neuron axon guidance [1], immune cell recognition [2], and angiogenesis [3]. These interactions involve sensing an attractant from the partner and responding by moving or growing in the appropriate direction (i.e. chemo-taxis/tropism), while secreting signaling molecules in a reciprocal fashion. This behavior is conserved in eukaryotes from fungi to humans [4,5].

The budding yeast *Saccharomyces cerevisiae*, undergoes a mating response that has served as a model system for studying cell-to-cell communication [6]. Yeast cells have two haploid mating types, **a** and α. By sensing the pheromone molecules (α-factor and **a**-factor), **a**- and α-cells detect the presence of a mating partner. These secreted peptides form a spatial gradient, bind to the pheromone-specific receptors, and elicit a response that includes cell-cycle arrest, gene expression, and formation of a mating projection (“shmoo”). Ultimately, the mating response results in the fusion of the two cells and nuclei to create an **a**/α diploid cell (reviewed in [7]).

Mathematical modeling has provided a useful tool for studying cell-cell interactions. Previously, moving interface models have been used to investigate deforming the shape of eukaryotic cells. In [8], a 1D continuum model of cell motility in amoeboid cells based on a viscoelastic description of the cytoplasm was developed, and in [9], cells in a 2D domain were treated as a two-phase fluid. The immerse boundary and finite element based approach was developed to model the actin network and cell morphogenesis in [10], an evolving surface finite element method modeled cell motility and chemotaxis in [11], and the boundary tracking Lagrangian framework was used in [12,13]. Other models used agent-based frameworks such as the Potts model, which takes into account detailed chemical networks and moving cells [14]. Level set approaches have also been adopted [15,16] to simulate the cell membrane deformation coupled to chemistry reaction dynamics. Previous studies focused on the relationship between morphogenesis and its underlying biochemical or mechanical machinery. In this work, we extend this concept by including the molecular dynamics within the extracellular space to study multi-cell interactions.

In laboratory yeast mating assays, wild-type cells mate with approximately 100% efficiency [17]. Genetic screens have identified mutants that mate at reduced efficiency [18]. One class of mutants prevents mating altogether. In addition, Hartwell and colleagues have modified the basic assay to investigate “three-way” mating between an **a**-cell that can mate with either an α-cell that makes α-factor or an α-cell that does not [19,20]. In this mating discrimination test, wild-type **a**-cells mate almost exclusively with α-factor producers. Mutations that affect the sensitivity of the system, such as the deletion of *SST2* (a gene which downregulates signaling via the heterotrimeric G-protein) or the deletion of *BAR1* (which encodes for an α-factor protease), dramatically reduce both mating efficiency and mating discrimination [20].

The communication between mating cells is mediated by the mating pheromones which bind their cognate G-protein-coupled receptors turning them on. Active receptor catalyzes the conversion of heterotrimeric G-protein into Gα-GTP and free Gβγ. The resulting Gβγ subunit can then recruit Cdc24 to the membrane where it activates Cdc42. Active Cdc42 is a master regulator of the cell polarity response orchestrating the cytoskeleton, exo/endocytosis, and signaling complexes [21,22]. All of these processes involve noise due to Brownian motion, stochasticity in gene expression or other intracellular fluctuations [23–26], which may affect cell assessment of signals and their responses [27]. In particular, the diffusion of ligand into the local neighborhood of the cell and the binding of ligand to receptor are thought to introduce significant stochasticity to gradient-sensing systems [24,28]. Therefore, it is necessary to consider the effects of noise when exploring cell behavior during mating.

There has been extensive mathematical modeling of the yeast pheromone response system. The early models were non-spatial and emphasized signaling dynamics [29–31]. More recent modeling efforts have incorporated spatial dynamics, both deterministic [32–34] and stochastic [35–37]. Models have ranged from simple generic formulations to detailed mechanistic descriptions. Finally, we and others have modeled pheromone-induced morphological changes to cell shape [12,38]. In related research, Diener et al. employed a combination of image processing and computational modeling to describe the extracellular α-factor dynamics in a population of mating cells, and how those dynamics were altered by the protease Bar1 [39]. However, missing from the literature is modeling of the yeast mating process itself involving both **a-** and α-cells.

In this paper, the goal was to construct the computational infrastructure for simulating the mating of two or more yeast cells, and then to investigate the factors responsible for robust mating behavior. We want to use our models to understand in greater detail the spatial dynamics that ensure efficient mating, and provide quantitative explanations and predictions on how perturbing these dynamics (e.g. mutants) disrupts the cell-cell interactions during mating. We succeeded in developing numerical methods for simulating yeast mating. Key elements include modeling the shape of the cell described by a moving boundary technique, and the extracellular diffusion dynamics of the pheromone ligand. Using this framework, we explored different model structures and parameters in a systematic fashion using generic models. We were able to simulate the high efficiency of mating among wild-type cells, and their ability to discriminate among partners that synthesized mating pheromone at different levels. Our simulations suggested that two critical factors ensuring robust mating under noisy conditions were the polarized secretion of mating pheromone, and the presence of the Bar1 protease. In addition, we demonstrated that supersensitive mutants disrupted both mating efficiency and discrimination, reproducing experimental data. More generally, this work makes progress toward the goal of a more detailed description of cell-cell interactions.

## Results

### 1. A stochastic model with dynamic cell shapes for multi-cell mating systems

In this section, we describe the stochastic model for multi-cell mating systems in two-dimensional space. Cell shape is represented by a level set formulation to capture the deforming plasma membrane induced by pheromone signaling.

**Fig 1:**
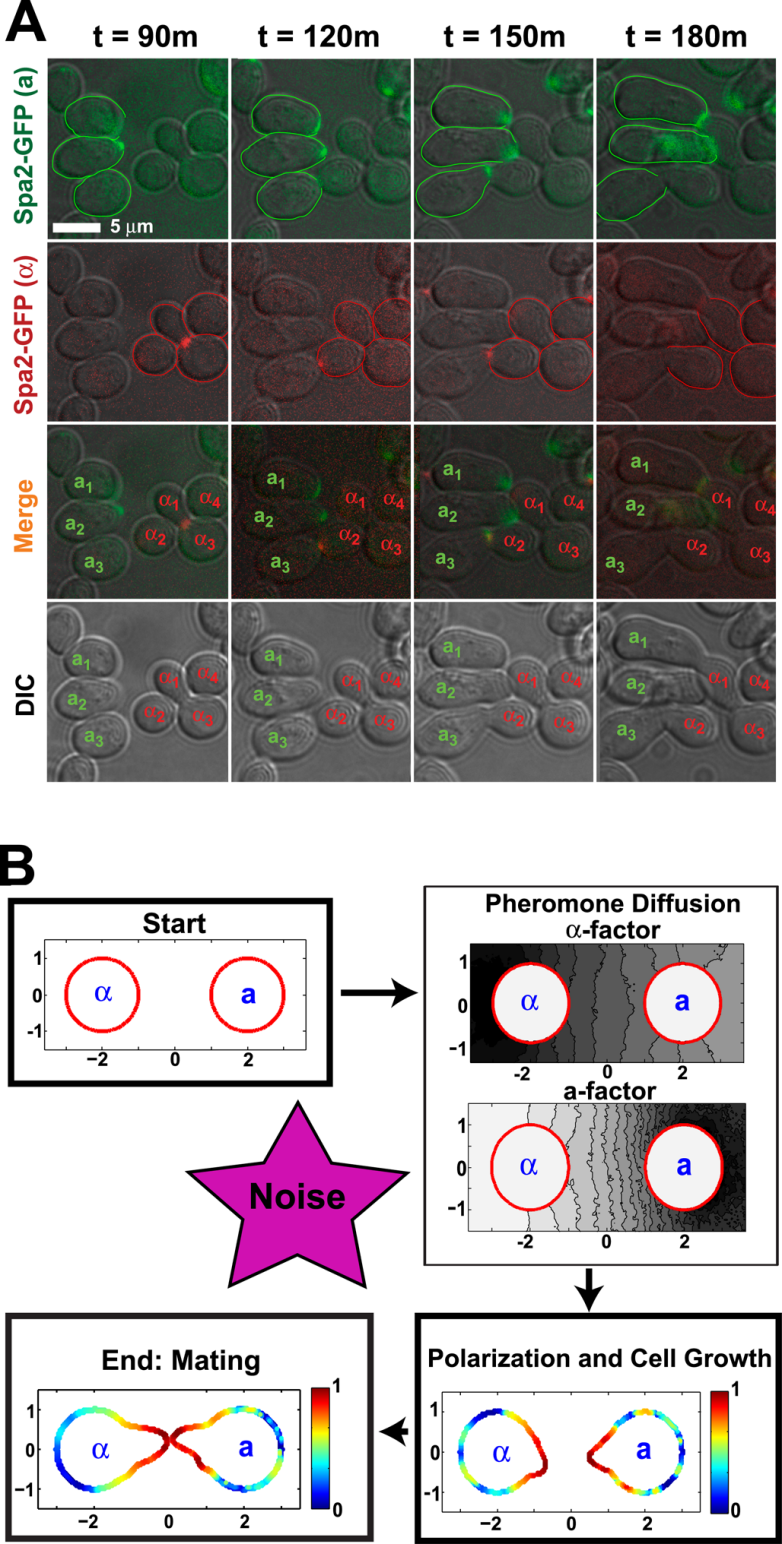
Yeast mating: experiments and simulations. **(A)** Time-lapse microscopy of mating yeast cells. Wild-type bar1Δ **a**-cells (left) and α-cells (right) were imaged over a two-hour period starting at t = 90m. The polarisome was labeled with Spa2-GFP (**a**, first row, cells outlined in green) and Spa2-mCherry (α, second row, cells outlined in red). The GFP and mCherry channels are merged (third row) with the three **a**-cells and four α-cells labeled. The DIC images are shown in the fourth row. The pairs **a**_**1**_-α_1_ and **a**_3_-α_2_ mated successfully. The presence of Bar1 over time will degrade the α-factor in a mating mix, and so to maximize the mating response we employed a bar1Δ strain. Scale bar = 5 μm. **(B)** Schematic diagram of yeast mating simulations. At the start, two cells are separated by 4 microns. The two mating pheromones (**a**-factor and α-factor) diffuse from their respective sources (**a**-cell and α-cell) which are sensed by the respective partners. The spatial dynamics of the biochemical reaction network are triggered resulting in the polarization of the membrane species. The boundary of the cell moves in response to the concentration of the polarized species resulting in the growth of a mating projection. Mating ends when the tips of the projections contact one another.

As described in the Introduction, mating occurs when an **a**-cell and α-cell are in close proximity (Fig 1A). They sense the pheromone gradient generated by the partner and project toward the source. In Fig 1A, cells are labeled with a marker for the polarisome (**a**: Spa2-GFP or α: Spa2-mCherry), a cellular structure at the tip of the mating projection. From a simulation standpoint, this process can be broken down into a series of steps (Fig 1B) from the secretion and diffusion of pheromones to the resulting growth in the mating projection.

Describing the mating process between two cells requires solving diffusion equations for the ligands in the extracellular space, which evolve according to the shifting positions of the pheromone sources. These sources in turn depend on the sensing of the ligand input and the morphological response. Thus, the cell boundary is evolved together with the molecular dynamics associated with the membrane for each cell.

Unlike our previous model of a single polarizing cell which solves surface reaction-diffusion equations in Lagrangian coordinates to capture deformation of the cell membrane [12], here we apply the level set method [40], which can track the moving curve front implicitly by solving a Hamilton-Jacobi equation. In this way, it is easier to study the interactions of multiple cells, and it allows a straightforward extension to the case of multicellular interactions by introducing level set functions for each different cell, and inclusion of the surface diffusions for molecules on the cell membrane. With this methodology, we can distinguish between the intracellular and extracellular space, and couple extracellular pheromone diffusion with the intracellular reaction-diffusion dynamics. The numerical scheme is described in the Methods section.

For simplicity, the cell is modeled as a two-dimensional (2D) circle with radius of 1 μm; the actual yeast cell is a three-dimensional (3D) sphere with radius 2 μm. The experimental mating assay involves placing the cells on a surface (i.e. paper filter) so that the mating reaction is effectively in two dimensions. The time unit is 100 seconds to approximate within an order of magnitude the growth velocity observed in experiments.

In this model, the mating pheromone is denoted by *f*, which is the external cue of cell polarization. Two membrane-associated species, *u*_*1*_ and *u*_*2*_, initially are uniformly distributed and then undergo polarization upon sensing the pheromone signal. The system forms a two-stage cascade in which the output of the first stage (*u*_*1*_) is the input to the second stage whose output is *u*_*2*_. The species *v*_*1*_ and *v*_*2*_ provide negative feedback (integral feedback) to regulate *u*_*1*_ and *u*_*2*_ (see S1 Text). The cell grows in the direction determined by *u*_*2*_. This model is a generic model of the mating system and abstracts away the mechanistic details of yeast mating.

As studied in the previous model for the two-stage yeast cell polarity system on a single cell [41], *u*_*1*_ represents the protein Gβγ, which is the output of the heterotrimeric G-protein system and the input to the Cdc42 system, and *u*_*2*_ represents active Cdc42, which is the master regulator of yeast cell polarization. Finally, the peak of the *u*_*2*_ distribution represents the polarisome which directs new secretion driving mating projection growth.

To track morphological changes of multiple cells, we use a level set function, denoted by *ϕ*(*x*, *t*), to distinguish exterior and interior of one cell such that the domain *D* is decomposed into three regions: Γ = {*x*: *ϕ*(*x*, *t*) = 0} representing the plasma membrane, Ω^*in*^ = {*x*: *ϕ*(*x*, *t*) < 0} corresponding to the intracellular space, and Ω^*ex*^ = {*x*: *ϕ*(*x*, *t*) > 0} is the extracellular space. The membrane is moving in a given velocity field ***V***(*x*) which is described in the Methods section and represents growth of the projection. The stochastic dynamics of the diffusing pheromone ligand (*f*_*α*_ represents α-factor and *f*_*a*_ represents **a**-factor) are described in (1) and (2):

$$\frac{\partial f_{\alpha }}{\partial t}=D_{\alpha }\Delta f_{\alpha }+S_{\alpha }\left(x,t\right)-k_{\alpha }f_{\alpha }+\kappa_{1}f_{\alpha }\frac{\partial^{2}W_{1}\left(x,t\right)}{\partial t\partial x},\;x\in \Omega^{ex}$$ (1)

$$\frac{\partial f_{a}}{\partial t}=D_{a}\Delta f_{a}+S_{a}\left(x,t\right)-k_{a}f_{a}+\kappa_{1}f_{a}\frac{\partial^{2}W_{2}\left(x,t\right)}{\partial t\partial x},\;x\in \Omega^{ex},$$ (2)

where *S*_*α*_(*x*, *t*) and *S*_*a*_(*x*, *t*) denote the sources of pheromones, and they are either constant or localized Gaussian distributions with support on the membrane (see Methods).

Each cell contains the membrane-associated species (*u*_*j*_, *v*_*j*_), *j = 1*,*2*, whose dynamics are described in Eqs (3–6), and the membrane velocity is described in (7):

$$\frac{\partial u_{1}}{\partial t}=D_{s}\Delta_{s}u_{1}+\frac{k_{10}}{1+{\left(\beta_{1}\tilde{f}\right)}^{-q_{1}}}+\frac{k_{11}}{1+{\left(\gamma_{1}u_{1}p_{1}\right)}^{-h_{1}}}-\left(k_{12}+k_{13}v_{1}\right)u_{1}+\kappa_{2}u_{1}\frac{\partial^{2}W_{3}\left(x,t\right)}{\partial t\partial x},\;x\in \Gamma$$ (3)

$$\frac{\partial v_{1}}{\partial t}=k_{14}\left({\tilde{u}}_{1}-k_{1ss}\right)v_{1}+\kappa_{3}v_{1}\frac{\partial^{2}W_{4}\left(x,t\right)}{\partial t\partial x},\;x\in \Gamma$$ (4)

$$\frac{\partial u_{2}}{\partial t}=D_{s}\Delta_{s}u_{2}+\frac{k_{20}}{1+{\left(\beta_{2}u_{1}\right)}^{-q_{2}}}+\frac{k_{21}}{1+{\left(\gamma_{2}u_{2}p_{2}\right)}^{-h_{2}}}-\left(k_{22}+k_{23}v_{2}\right)u_{2},\;x\in \Gamma$$ (5)

$$\frac{\partial v_{2}}{\partial t}=k_{24}\left({\tilde{u}}_{2}-k_{2ss}\right)v_{2},\;x\in \Gamma$$ (6)

$$V\left(x,t\right)=V_{amp}\cdot u_{2}\cdot \text{max}\left(0,\langle \vec{n},{\vec{d}}_{\text{max}}\rangle \right),\;x\in \Gamma$$ (7)

$$\tilde{f}=\frac{f}{\underset{x\in S}{\text{max}}f}+0.1,\;{\tilde{u}}_{1}=\frac{\int_{s}u_{1}ds}{\int_{s}ds},\;{\tilde{u}}_{2}=\frac{\int_{s}u_{2}ds}{\int_{s}ds},$$

$$p_{1}=\frac{k_{10}}{1+{\left(\beta_{1}\tilde{f}\right)}^{-q_{1}}},\;p_{2}=\frac{k_{20}}{1+{\left(\beta_{2}u_{1}\right)}^{-q_{2}}}.$$

Note that Eqs (3–7) are restricted on the plasma membrane Γ, where Δ_*s*_ denotes the surface Laplace-Betrami operator for the lateral surface diffusion. In Eq (3), $\tilde{f}$ is associated with the pheromone factor from the opposite mating type; that is, if the cell is an **a**-cell, then $\tilde{f}$ is ${\tilde{f}}_{\alpha }$. In addition, instead of *f*, we use the normalized distribution $\tilde{f}$; in this definition, a constant is added to make the parameter consistent with [12]. This normalization represents the adjustable dynamic range mechanisms in the system designed to prevent the sensing from saturating (see Discussion). In Eqs (3–6), we ignore the advection terms ∇_*S*_⋅(*u*_*j*_***V***) and ∇_*S*_⋅(*v*_*j*_***V***), which describe the increased surface area where the membrane species reside, because this dilution effect is minimized by the integral control feedback in the model (see S1 Text). On the other hand, these terms necessitate a smaller time step because of the curvature appearing during the computation. Therefore, for numerical efficiency, we simulate Eqs (3–6) on the deforming membrane without the advection terms.

In Eq (7), *V*(*x*,*t*) denotes the normal component of the growth velocity of the plasma membrane which describes the rate and direction of membrane movement (the tangential component is ignored by assuming it is small). *V*_*amp*_ is a constant specified with respect to the time scale, $\vec{n}$ is the unit outward normal vector, and ${\vec{d}}_{\text{max}}$ is the growth direction defined as the unit outward normal vector at the center of the polarisome. The normal velocity *V(x*,*t)* is assumed to be proportional to *u*_*2*_, i.e., active Cdc42. The concentration and location of active Cdc42 determines the position of the polarisome, which directs the secretion [21]. We model the growth direction to be aligned with the normal direction at the polarisome.

Multiplicative noise was adopted and each of the noise terms was weighted by a parameter *κ*_*i*_, representing external or internal noise sources. The function *W*_*i*_(*x*, *t*) is a random variable such that the white noise term $\frac{\partial^{2}W_{i}\left(x,t\right)}{\partial t\partial x}$ follows a normal distribution with variance the same as the time step according to the definition of a Wiener process in our simulations. For simplicity, we considered three noise effects in the simulations. One represents the diffusive noise of the extracellular ligands which is described in the pheromone equation. The second is associated with the dynamics of *u*_*1*_ (Gβγ) which represents noisy internal processes such as fluctuations in ligand-receptor binding and receptor activation of G-protein. The final noise effect represents noise in the regulatory feedback loop (*v*_*1*_). Noise introduced in the second stage of the model is ignored to focus on the sensing noise. In addition, we modified the definition of the velocity function in the stochastic model. Since the velocity depends on *u*_*2*_ which is fluctuating, it is necessary to apply filtering to smooth the dependence of the velocity function on *u*_*2*_ (see S1 Text). Finally, we can explore the deterministic dynamics simply by choosing zero for each *κ*_*i*_.

The default initial conditions for the simulations are two cells (one **a**-cell and one α-cell) whose centers are separated by 4 μm in which the membrane species *u*_*i*_ is uniformly distributed on the cell surface, and thus no polarisome is formed in the beginning and initial pheromone secretion will be isotropic. Unless otherwise stated, no Bar1 (α-factor protease) is present, i.e., cells are considered bar1Δ; there is no background α-factor source.

At this point we note some of the limitations of the model which we expand upon in the Discussion. First the model is a generic representation of the system that lacks mechanistic detail. Second we employed a quasi-steady-state approximation of α-factor spatial dynamics to speed up the simulations. Third there was not rigorous fitting of the parameters to the experimental data but rather a sampling of different regions of parameter space that produced experimentally observed behaviors.

### 2. Noise disrupts mating alignment between two cells

In this section, we investigated the impact of noise in the context of exploring one specific parameter in the simulations, the **a**-factor diffusion constant.

#### 2.1. Varying the diffusion rate of a-factor without noise

**Fig 2:**
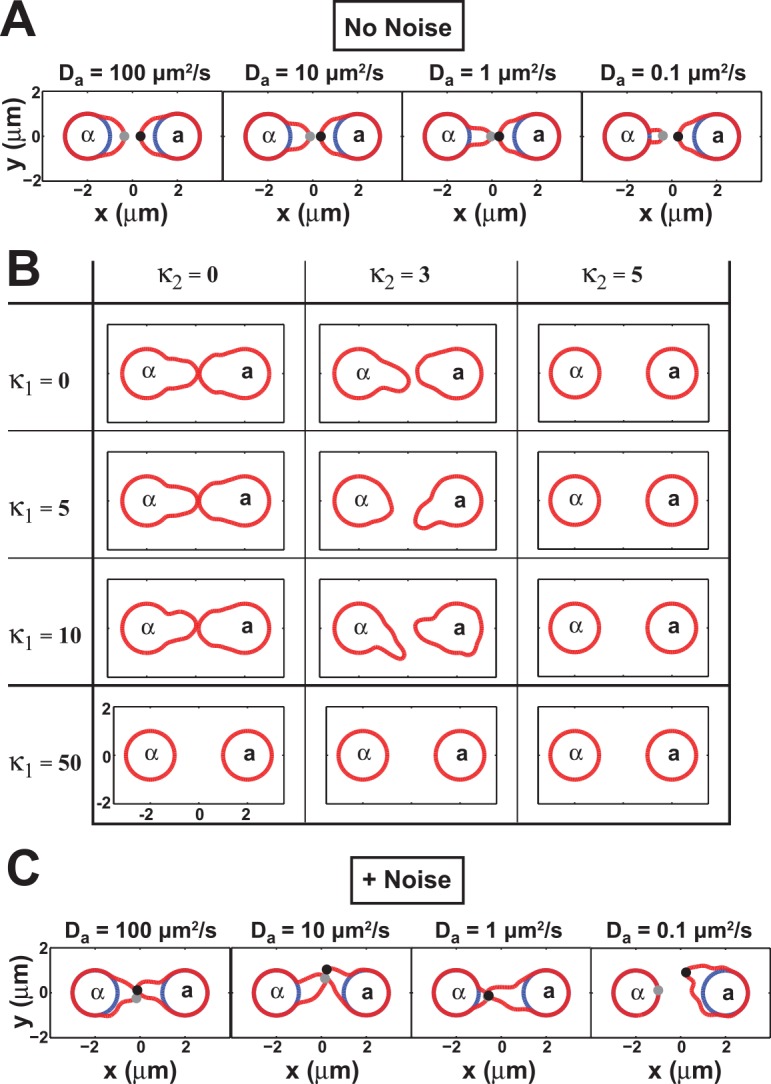
Effect of external and internal noise on yeast mating simulations. **(A)** Varying **a**-factor diffusion rates under no-noise simulation conditions. The diffusion constant for **a**-factor *D*_***a***_ was set to 0.1, 1, 10, and 100 μm^2^/s; the diffusion constant for α-factor was *D*_*α*_ = 100 μm^2^/s. The cell centers were separated by 4 μm at the start. In all cases the cells were able to grow toward each other successfully. **(B)** External and internal noise disrupt mating. External (*κ*_*1*_) and internal (*κ*_*2*_) noises were added to the simulations using a range of values (0, 5, 10, 50 for *κ*_*1*_; 0, 3, 5 for *κ*_*2*_). 10 simulations were run for each combination, and an example simulation is shown for each specified pair of values (*κ*_*1*_, *κ*_*2*_). **(C)** Varying **a**-factor diffusion rates in the presence of noise. Simulations performed as in (A) except in the presence of noise (*κ*_*1*_ = 5, *κ*_*2*_ = 3). All simulations produced mating except *D*_***a***_ = 0.1.

The diffusion constants for the pheromones α-factor and **a**-factor are known to be different. Because α-factor is water-soluble, its diffusion constant can be estimated to be *D*_*α*_ ~ 100 μm^2^/sec based on its molecular weight [42]. The diffusion coefficient for **a-**factor is thought to be lower than α-factor because of its hydrophobic tail, but the value is not known [43]. We investigated a range of values for this parameter, using our two-cell model in the absence of noise. As shown in Fig 2A, the diffusion rate *D*_***a***_ was varied to be 100, 10, 1 and 0.1 μm^2^/s. Overall the mating behaviors were similar (cells projected toward one another) although the different diffusion coefficients of **a**-factor give rise to different morphologies for the α-cell.

#### 2.2. Varying the external and internal noise amplitude

We investigated different values for the external (*κ*_*1*_) and internal (*κ*_*2*_) noise in the simulations. For simplicity, we set the value of noise on the negative feedback term to be a constant (*κ*_*3*_ = 0.1) in all simulations in order to focus on *κ*_*1*_ and *κ*_*2*_. In Fig 2B, we show a table of typical simulations in which *κ*_*1*_ was varied from 0 to 50, and *κ*_*2*_ was varied from 0 to 5. *D*_***a***_ was set to a test value of 10 μm^2^/s to reflect the fact that the lipid-modified **a**-factor is expected to diffuse slower than α-factor (which has no lipid modification) but faster than a lipid-modified membrane protein (0.1–1 μm^2^/s). The sample simulation indicates the effect of a given level of noise and is representative of at least 10 trial simulations.

Both types of noise make mating more challenging. For *κ*_*1*_ (external), low levels are similar to no noise. At intermediate levels of *κ*_*1*_ we observe less accurate mating. At high levels, the cells are unable to polarization. The same trend is observed with the internal noise parameter *κ*_*2*_ albeit with an even bigger effect. Combining the two noise terms further decreases mating and polarization. Based on these results, we selected intermediate values for both *κ* parameters, and set the noise amplitudes to be *κ*_*1*_ *=* 5, *κ*_*2*_ *=* 3 in the stochastic simulations that follow unless otherwise specified. This level of noise disrupts mating alignment but does not prevent mating. These values are consistent with previous theoretical work [25], which when applied to the yeast system have led to estimates of external noise arising from ligand diffusion and internal noise arising from ligand-receptor interaction to be in the range from 1 to 10 [28,41].

#### 2.3. Varying the diffusion rate of a-factor with noise

We re-tested the different values for the diffusion coefficient of **a**-factor in the presence of noise (Fig 2C) using the noise parameters *κ*_*1*_ *=* 5, *κ*_*2*_ *=* 3. Compared to the no-noise simulations (Fig 2A), we observed that mating did not occur for the lowest value of *D*_***a***_ = 0.1 μm^2^/s. One explanation is that at very low values of *D*_***a***_ then **a**-factor does not diffuse far enough to influence mating during the time period. On the other hand, values of 100, 10, and 1 all resulted in mating; we chose a default value of 10 μm^2^/s to maintain the asymmetry between **a**-factor and α-factor while avoiding low values that hinder mating.

### 3. Mating Efficiency of Two Cells

#### 3.1. Defining mating efficiency

A standard laboratory test of yeast mating is the mating efficiency assay [17]. Populations of **a**- and α-cells are mixed together and the percent of successful matings is calculated, i.e., percent of **a**-cells that have mated with α-cells to form diploids divided by the total number of **a**-cells. Below we attempt to reproduce this assay using the two-cell mating simulations. This approach is a simplification because it ignores the influence of surrounding cells. Later, we describe three- and five-cell simulations that take into account more cells.

**Fig 3:**
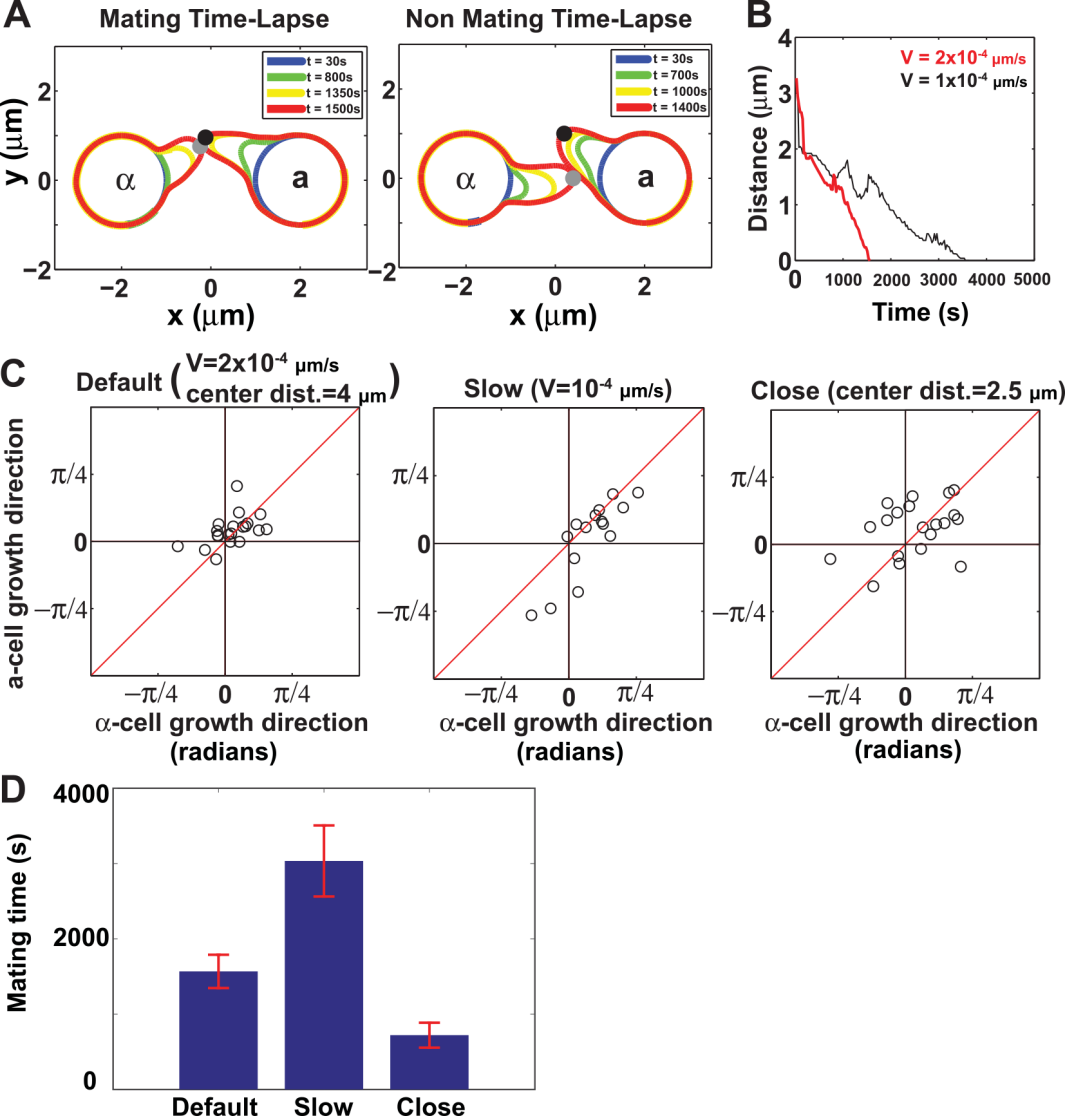
Computer simulations of mating efficiency. (A) Schematic of determining mating efficiency. In the simulation on the left, the tips marked by gray and black dots (polarisomes) of the two projections fall within a distance threshold (see **Methods**) so that the mating is considered successful. In the simulation on the right, the tips do not pass close enough to one another by the end of the simulation, and so the mating is deemed unsuccessful. (B) Faster and slower boundary velocities yielded similar mating trajectories. We ran simulations at two different boundary velocities (*V*_*amp*_ = 0.0001 and *V*_*amp*_ = 0.0002 μm/s). A plot of the distance between polarisomes of mating partners as a function of time is shown for a sample simulation. The plots are similar except the slower velocity took approximately twice as long to mate. (C) Direction plots for different boundary velocities and shorter cell-to-cell distance. In this plot each data circle represents one mating simulation. The average direction of each projection is plotted on the *x*-axis for the α-cell, and *y*-axis for the a-cell. The projections are toward one another when the data point lies along the diagonal line (i.e. top right and bottom left quadrants). We show the direction plots for the default simulation parameters (V = 0.0002 μm/s, left), slow boundary simulation (V = 0.0001 μm/s, middle), and close-cell positions (cell-to-cell distance = 2.5 μm instead of 4 μm, right). The mating efficiencies were similar for all three simulations. (D) Average mating time of successful matings under different simulation conditions same as in (C). Each bar represents the average time (± standard deviation) for successful mating. We performed 20 simulations for each condition, and the numbers of successful matings for default, slow and close parameters are 15, 17 and 16 respectively.

In the simulations, two cells were started with their centers 4 μm apart to leave sufficient room for the cells to grow while the mating occurs in a reasonable amount of time which is set to be 1800 seconds in our simulations. A successful mating was defined as the focal region of *u*_*2*_ (i.e. polarisome) from each cell coming into close contact (Fig 3A, see Methods). Snapshots of simulations at different time are provided in Fig F in S1 Text.

As a negative control, we performed simulations in the absence of pheromone secretion in which pheromone was added exogenously to create a uniform distribution. In this case, the cells did not mate (see S1 Text) consistent with the low mating efficiency of pheromoneless cells observed experimentally [44].

#### 3.2. Varying velocity only affects the time of cell mating without changing efficiency

We tested different growth velocities by a 2-fold change to measure the effect on mating behavior in the simulations. Snapshots of simulations at different time points are provided in Fig G in S1 Text. The distance plot (which measures the distance between polarisomes every 50s) for a typical simulation at each velocity shows a steady decrease in the distance between projections. Moreover, when the velocity is decreased in half, it takes approximately twice as long to mate (Fig 3B and 3D). One can also examine the direction of each projection to see if they are growing toward one another. The angle of projection of the α-cell is defined in a counterclockwise fashion taking values from $-\frac{\pi }{2}$ to $\frac{\pi }{2}$, whereas the angle of projection of the **a**-cell is defined in the clockwise direction so that two cells grow toward each other when the projection angles are the same indicated by the diagonal line in Fig 3C. The growth direction is collected every 50s, and the average for each simulation represents a point in the direction plot. The points of the direction plots for the default (0.0002 μm/s) and slow (0.0001 μm/s) velocities both lie on the red diagonal line reflecting equivalent mating efficiencies (Fig 3C, mating efficiency ME = 17/20 for slower velocity and 15/20 for default boundary velocity, p-value is 0.69 by Fisher’s exact test). Mating efficiency is calculated by dividing the number of successful matings (described above) by the total number of mating simulations. Based on the results, the mating efficiency is not significantly changed by velocity and we set the velocity amplitude to be 0.0002 μm/s for faster simulations.

#### 3.3. Shorter cell-cell distance yields similar results

The distance between two cells can affect their interactions. We compared the default distance of 4 μm between the cell centers with a shorter distance of 2.5 μm (0.5 μm between cell boundaries). Snapshots of simulations at different time are provided in Fig H in S1 Text. The mating efficiency was 15/20 for the 4 μm distance and 16/20 for the 2.5 μm distance, which were very similar (p-value is 1 by Fisher’s exact test). Although the direction vectors showed increased variability reflecting a more scattered distribution around the diagonal at the shorter distance (Fig 3C), ultimately the two projections were able to find each other. Thus, at the shorter distance, mating efficiency was approximately the same as at the default distance. When we measured the mating time for successful matings (Fig 3D), we saw that the mating time was approximately inversely proportional to the membrane velocity and proportional to the distance between cell membranes.

In summary we observed a mating efficiency of approximately 75% in two-cell simulations at two different distances. This mating efficiency is slightly lower than the 90–100% mating efficiency observed in experiments [19,45].

### 4. Robustness Strategies for Optimizing Mating Efficiency

In the natural environment, yeast mating is efficient and robust to a variety of perturbations. In this section, we explored how features of the mating process could promote robust and efficient mating; we compared different mating scenarios by modifying the model parameters.

#### 4.1. Polarized pheromone source distribution increases mating efficiency

**Fig 4:**
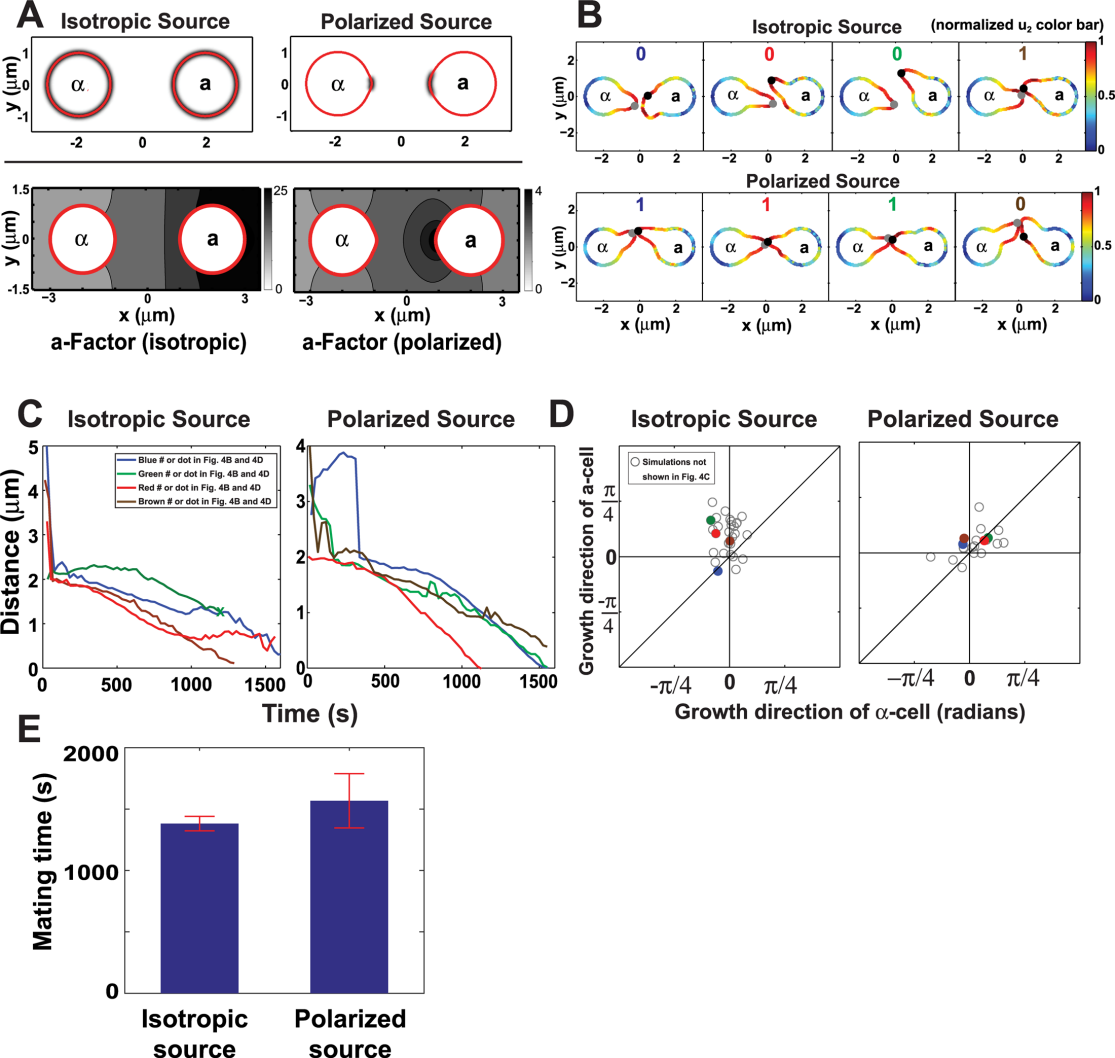
Mating efficiency of isotropic versus polarized pheromone source. **(A)** Schematic diagram of isotropic versus polarized (non-isotropic) pheromone source. Top row indicates in black shading the spatial distribution of the pheromone source function. The bottom row depicts the **a**-factor diffusion profile shown as a concentration contour plot for the isotropic source (left) and the polarized source (right). **(B)** Polarization plots of *u*_*2*_ showing mating cells at end of simulation. Four sample simulations each from the isotropic source group and from the polarized source group are shown. The normalized level of *u*_*2*_ is color coded on the surface of the cell according to the colormap on right. The polarisome is denoted by the black or gray dot at the projection tip. The polarized source produces higher mating efficiencies; the 1 or 0 indicates a successful or unsuccessful mating. The polarization plots, distance plots, and direction plots are color coded (blue, red, green, brown) for a particular simulation. **(C)** Distance plots for each of the four simulations. These plots show the distance between polarisomes of the mating partners as a function of time. With the isotropic source, the distances do not converge to 0 for some of the simulations. The green isotropic source simulation was terminated early because it did not meet the distance/direction threshold. **(D)** Direction plots for polarized source and isotropic source simulations. Each data point represents the averaged direction of the projection from each cell during mating. Axes are described in the legend to Fig 3C. Mating is more likely if the projections are in the same direction i.e. along the diagonal in the top right or bottom left quadrants. The average distance from the diagonal is 0.26 radians for the isotropic source compared to 0.12 for the polarized source matings. Colored filled circles correspond to simulations shown in (B) and (C). **(E)** Average mating time of successful matings with isotropic and polarized sources. Each bar represents the average time (± standard deviation) for successful matings. We performed 20 simulations for both conditions, and the numbers of successful matings for isotropic and polarized sources are 6 and 15 respectively.

One important variable is the spatial distribution of the pheromone source. We consider two possibilities with respect to the pheromone source: isotropic or non-isotropic (polarized) secretion. In the isotropic scenario, pheromone is secreted uniformly from all points on the cell surface. In the non-isotropic scenario, the source would be polarized to the front (Fig 4A). Intuitively, one may imagine that the polarized source distribution would contribute to accurate mating by helping the projections find each other, and in the simulations described above we used the polarized source as the default. However we wished to compare these two possibilities quantitatively as follows.

At the start of the simulations both cells secrete isotropically. Once the polarization is activated, the secretion becomes localized and is confined around the growth tip by being a function of *u*_*2*_ in the formulation of the polarized (non-isotropic) source distribution, whereas the isotropic function does not depend on *u*_*2*_ (see Methods).

As expected the polarized pheromone source matings produced higher mating efficiency (ME = 15/20) than the isotropic source matings (ME = 6/20). By carrying out Fisher’s exact test on the mating efficiency, p-value is 0.01, therefore the difference between the two ME scores is significant. The effect could be observed in four sample matings for each scenario. Snapshots of more examples at different times are provided in Fig I in S1 Text. A picture of the mating cells (Fig 4B) shows how with the polarized source the projections tend to meet, whereas with the isotropic source, they sometimes miss. The distance plots measure the distance between the polarisomes over the course of the simulation (Fig 4C). With the isotropic matings, sometimes the distance stops decreasing and begins increasing as the projections go past each other. With the polarized source matings, the projections tend to go toward each other so that the distance steadily decreases.

In addition, the direction plots (Fig 4D) show that compared to the polarized source case, the isotropic matings possess projection directions that are not always toward one another (i.e. points farther off-the-diagonal; 0.26 versus 0.12 radians). The polarized source mating projections are all in the two quadrants along the diagonal in which the projections are heading in the correct direction. However, when we measured the average mating time of successful matings for isotropic and polarized sources (Fig 4E), we did not observe a significant mating time difference if the mating is successful.

#### 4.2. Supersensitive cells exhibit decreased mating efficiency

For yeast cells, one challenge is keeping the ligand concentration in the proper range so that the cell can detect spatial differences. In mutant cells (e.g. *sst2Δ*) that are overly sensitive to pheromone (supersensitive), the signaling system becomes easily saturated and the cell cannot determine the concentration. As a result, they cannot detect the correct gradient direction and fail to mate [19]. We represented supersensitivity in our model by increasing the parameter *β*_1_, the reciprocal of the value achieving half-maximal activation in the term modeling external stimuli.

**Fig 5:**
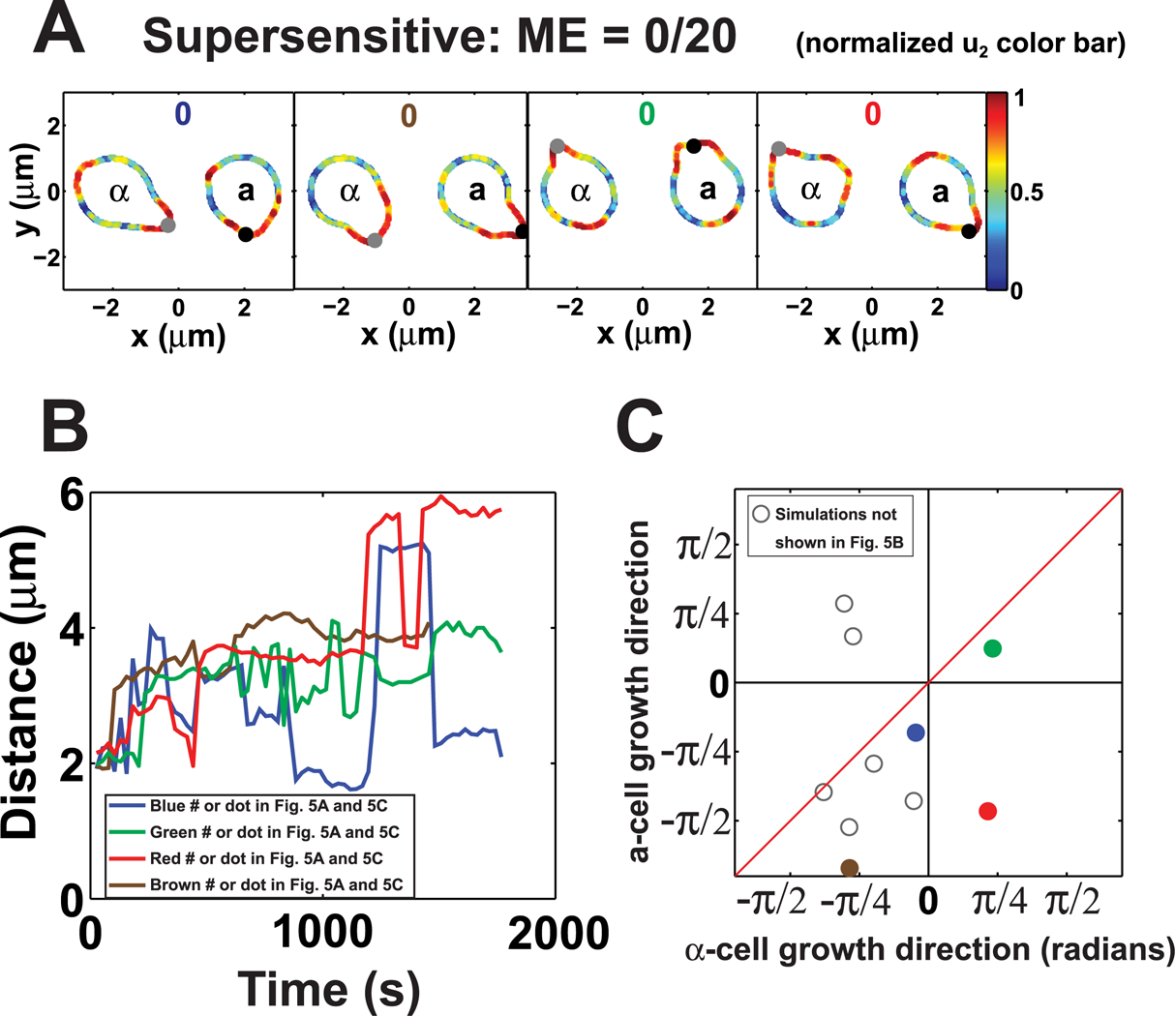
Reduced mating efficiency for supersensitive cells. **(A)** Polarization plots of *u*_*2*_ showing four pairs of supersensitive mating cells at the end time point. The spatial distribution of *u*_*2*_ is represented according to the normalized color map on the right. The 0’s indicate that none of the matings were successful. Both cells had *β*_*2*_ = 2.5. **(B)** Distance plot showing trajectories from four sample supersensitive mating simulations. The distance between polarisomes was plotted as a function of time. The distances did not steadily decrease as was observed in the normal sensitivity simulations. The four plots are color-coded to match with the polarization plots. **(C)** Direction plot for supersensitive cell mating simulations. Many of the data points lie far off the diagonal indicating that the cells are not pointing toward each other; the average distance from the diagonal is 0.68 radians compared to 0.12 for the normal sensitivity matings.

We tested the case *β*_1_ = 2.5 (*β*_1_ = 0.92 is default value) for both cells in two-cell mating in a noisy environment (Fig 5). In the presence of noise, both cells can successfully make a projection. Although the α-cell can detect the gradient and grow toward the source, the **a**-cell cannot. The growth of the **a**-cell is triggered by noise fluctuations, so that the cell picks a random direction which may not be correlated with the gradient. As a result, no matings are observed in the supersensitive simulations (ME = 0/20, p-value = 7.7E-07 by Fisher’s exact test). Snapshots of more simulations at different time points are provided in Fig J in S1 Text.

In summary, we observed dramatically reduced mating efficiency in supersensitive cells with ME = 0% in the simulations compared to the 75% in cells possessing normal sensitivity. Experimentally, past data from this lab showed a decrease in mating efficiency from 96% in wild-type cells to 28% in *sst2Δ* supersensitive cells [45].

#### 4.3. The presence of the α-factor protease Bar1 improves mating efficiency

The **a**-cell can secrete a protease, Bar1, to degrade α-factor in its vicinity during mating. Up to this point, we have not tried to model Bar1; in effect, the **a**-cells have been bar1Δ mutants. It is thought that cells without Bar1 mate less efficiently than cells with Bar1 [46]. One explanation is that the background level of α-factor increases without the presence of Bar1. This background level can saturate the sensing apparatus in a similar fashion experienced by the supersensitive mutants preventing gradient detection.

In the previous work [12,38], the authors suggest that Bar1 is necessary for efficient mating by reshaping the local pheromone concentration and avoiding nonproductive cell-cell interactions. To investigate this process, we compared the behavior of Bar1+ and bar1Δ cells at different background pheromone production rates.

For a Bar1+ cell, we need to solve one more equation in extracellular space for the protease distribution, which is formulated similarly to Eq (1),

$$\frac{\partial B}{\partial t}=D_{B}\Delta B+S_{B}\left(x,t\right)-k_{B}B,\;x\in \Omega^{ex}.$$ (8)

The equation of α-factor is modified to be

$$\frac{\partial f_{\alpha }}{\partial t}=D_{\alpha }\Delta f_{\alpha }+S_{\alpha }\left(x,t\right)-\left(k_{\alpha }+k_{Bf}\tilde{B}\right)f_{\alpha },\;x\in \Omega^{ex},$$ (9)

in which $\tilde{B}=\frac{B}{\text{max}\;B+\varepsilon }$ represents the degradation of α-factor by Bar1, and *ε*(≈ 10^−6^) is added to avoid zero in the denominator. We also set the production of Bar1 to be $S_{B}=\frac{1}{50}S_{a}$ in the simulations. Therefore, if the **a**-factor is secreted in a polarized fashion, then so is Bar1.

We simulated the increase in background α-factor with a new uniform source function $\left(S_{\alpha }^{'}=S_{\alpha }+C\right)$ that increased the global α-factor levels (by the constant *C*) while the cells generated the local α-factor dynamics (*S*_*α*_). For high values of *C*, the simulated bar1Δ cells did not mate as efficiently (Table 1).

**Table 1:** Mating efficiency: Bar1+ versus bar1Δ simulations.

	Background α-Factor production rate (C)			
	0	1	10	100
**bar1Δ**	15/20	9/20	5/20	2/20
**Bar1+**	20/20	18/20	17/20	16/20
p-value	0.024	0.0029	0.00016	8E-06

We then tested for the effect of the presence of Bar1. When *C* = 0, the bar1Δ and Bar1+ cells displayed approximately the same mating efficiency. For the bar1Δ simulations, as we increased *C* from 0 to 100, there was a progressive decline in mating efficiency from 15/20 to 2/20. For the Bar1+ simulations, there was also a decline but it was more gradual. At *C* = 10, the mating efficiency was still 85% and at *C* = 100 it was 80%, which was higher than the corresponding bar1Δ values. To test whether the mating efficiency of Bar1+ is significantly greater than that of bar1Δ, we performed Fisher’s exact test on H_0_ (null hypothesis): ME_Bar1+_ = ME_bar1Δ_, versus H_A_ (alternative hypothesis): ME_Bar1+_> ME_bar1Δ_ for the different production rates of background α-factor. At different values of *C*, the p-values are all less than 0.05 so that we can reject H_0_ at the 95% confidence level. Thus, in the simulations, Bar1 improves mating efficiency at all levels of background α-factor especially at higher production rates. The trend that increasing background α-factor decreases mating efficiency especially in bar1Δ cells is consistent with past experimental observations [19,44].

### 5. Mating competition among three cells: Bar1 adjusts pheromone gradient to attract mating partner

**Fig 6:**
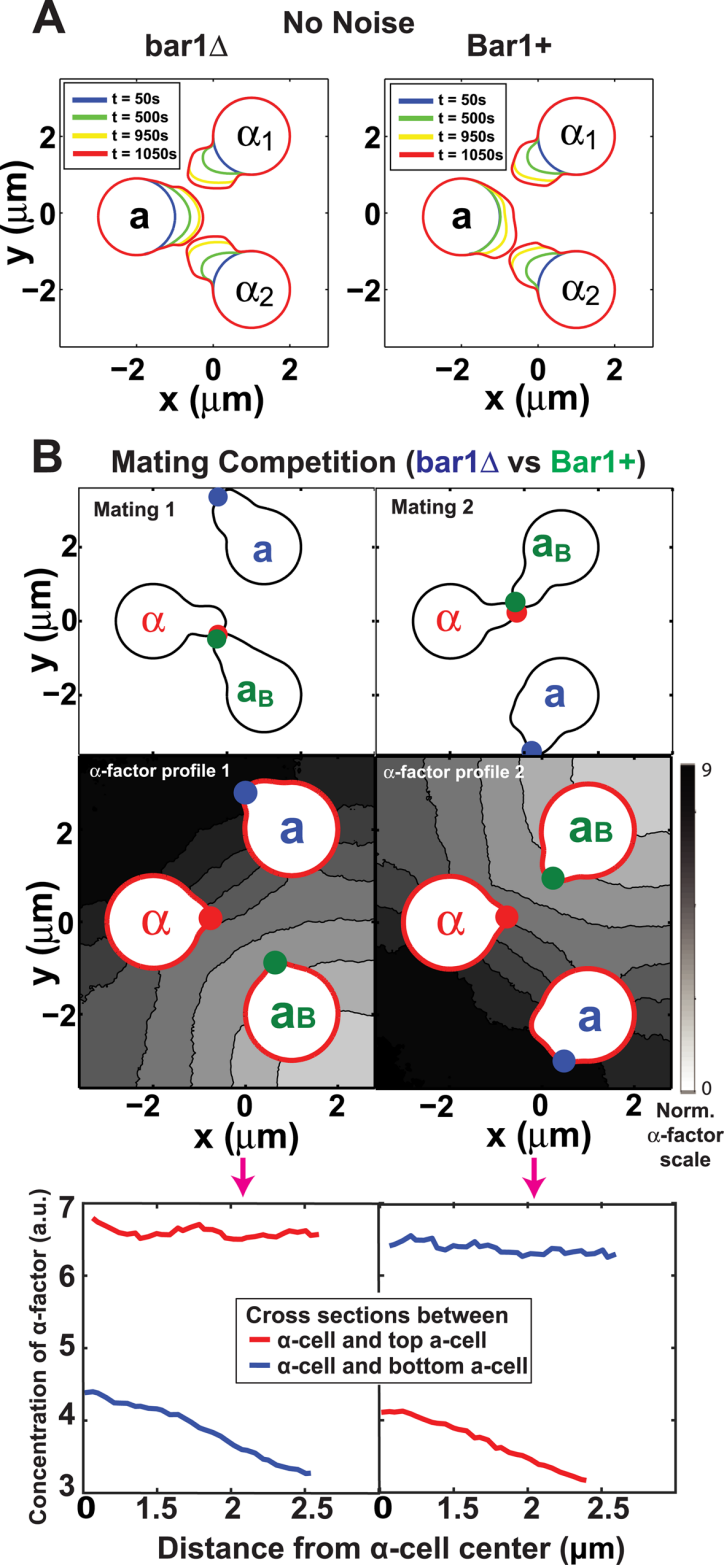
Three-cell simulations. **(A)** Bar1 helps **a**-cell distinguish closer α-cell. Two α-cells and one **a**-cell were positioned approximately at the vertices of a triangle with one of the two α-cells slightly closer to the **a**-cell than the other. We tested whether a Bar1+ or a bar1Δ **a**-cell could distinguish between the two α-cells in simulations performed in the absence of noise. The Bar1+ cell projected toward the closer α-cell, whereas the bar1Δ cell projected toward the middle between the two α-cells. **(B)** Mating competition simulations in which two **a**-cells compete for a single α-cell. In these three-cell simulations, one **a**-cell is Bar1+ and the other is bar1Δ. In 20/20 simulations, the Bar1+ cell mated with the α-cell, and two sample simulations are shown. At the top are snapshots with the projections in contact. In the middle are the α-factor profiles from the two simulations, which show how the high concentration of α-factor in the absence of Bar1 precludes gradient detection. At the bottom is the α-factor distribution along the cross-section between the α-cell and **a**-cell. In both cases, the steeper gradient is observed with the Bar1+ **a**-cell.

A natural extension of two-cell mating simulations is three-cell mating simulations. In three-cell simulations, the set-up can be either two α-cells and one **a**-cell, or two **a**-cells and one α-cell. In the former case, if the two α-cells are equidistant from the **a**-cell, we found that in the absence of noise, the **a**-cell projected toward the middle in between the two α-cells. Interestingly, if the two α-cells are slightly offset (i.e. the **a**-cell is located 0.1 microns below the middle line of two α-cells so that one is closer), then the **a**-cell still projected toward the middle (Fig 6A). If the **a**-cell is Bar1+, then the **a**-cell is able to gradually reorient to the closer mating partner. However adding noise to the simulations, the Bar1+ **a**-cell projected toward one or the other α-cell in a random fashion whether or not the cells were offset. Although the no-noise case is somewhat artificial, it indicates how Bar1 can improve the ability to detect the gradient direction in this idealized scenario.

Alternatively, the simulation can be between one α-cell and two **a**-cells. If the two **a**-cells have different genotypes, then there is a competition between the two for the single α-cell. This corresponds to mating competition experiments, a second important type of mating assay [47], in which one mixes two **a**-cell genotypes with a limiting quantity of α-cells. We tested the importance of Bar1 using mating competition. In mating competition simulations between Bar1+ and bar1Δ cells we found that the Bar1+ cells mated with the single α-cell partner 20/20 times (Fig 6B). Snapshots of more simulations are provided in Fig K in S1 Text.

Greater insight on why the Bar1+ cell has the advantage can be provided by the α-factor profiles for two sample simulations (Fig 6B, lower). The Bar1 helps to remove the excess α-factor so that the Bar1+ **a**-cell is able to sense the gradient from the α-cell. The bar1Δ cell is stuck in a region of high α-factor in which the gradient is shallower.

### 6. Mating discrimination among multiple cells

The third mating arrangement is having a single **a**-cell choose between two α-cells of different genotypes. One specific scenario is having one α-cell make α-factor whereas the other α-cell makes less or no α-factor. Experimentally this simulation corresponds to a third important type of mating assay: mating discrimination in which the **a**-cell must discriminate between the α-cell mating partner secreting α-factor from α-cell decoys that do not [20,47]. This assay measures the ability of an **a**-cell to sense and respond accurately to a pheromone gradient.

#### 6.1 Mating discrimination for three-cell mating: The a-cell chooses the mating partner producing more pheromone

**Fig 7:**
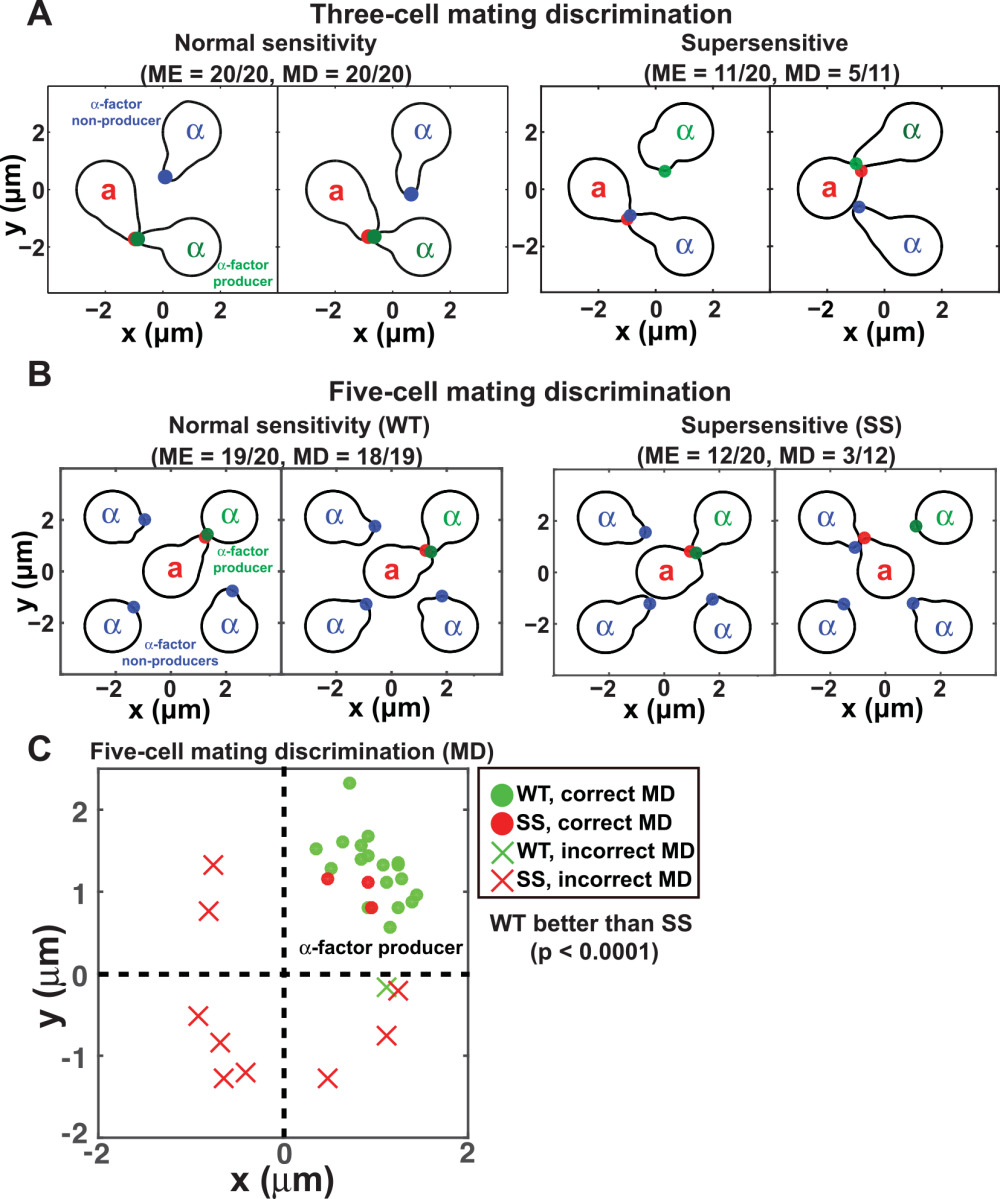
Modeling mating discrimination. **(A)** Three-cell mating discrimination simulations. One **a**-cell and two α-cells were arranged so that the **a**-cell was equidistant from the α-cells. One α-cell makes α-factor (α-factor producer, green) and the other α-cell does not (α-factor non-producer, blue). 20 simulations were run to determine the ratio at which the **a**-cell would mate with the α-factor producer versus the non-producer. Two sample simulations are presented. The left panel shows an **a**-cell with wild-type sensitivity, and the right panel shows a supersensitive **a**-cell. ME indicates mating efficiency; MD indicates mating discrimination. **(B)** Five-cell mating discrimination simulations. Four α-cells are arranged in a square with one bar1Δ **a**-cell in the center. One α-cell makes α-factor (α-factor producer, green) and the other three cells α-cell do not (α-factor non-producers, blue). 20 simulations were run to determine mating discrimination, and two sample simulations are presented. The left panel shows an **a**-cell with wild-type sensitivity, and the right panel shows a supersensitive **a**-cell. **(C)** Mating location plots for **a**-cells possessing normal sensitivity (WT, green) or supersensitivity (SS, red) in five-cell mating discrimination simulations. Each dot (correct MD) or cross (incorrect MD) symbol represents the polarisome location of the **a**-cell at the time of mating. The α-cell producing α-factor was in the top-right quadrant. The cells possessing normal sensitivity showed significantly better mating discrimination (MD) than the supersensitive cells (p < 0.0001, Fisher’s Exact Test).

The first arrangement we tested was to have one **a**-cell (bar1Δ) and two different α-cells; one α-cell makes α-factor and the other does not. In the 20/20 simulations in which a successful mating occurred, the **a**-cell mated with the correct partner thus exhibiting perfect mating discrimination (Fig 7A). Snapshots of more simulations are provided in Fig L in S1 Text.

Experimentally it is known that supersensitive cells exhibit reduced mating discrimination along with lower mating efficiency. We tested the scenario in which the **a**-cell is supersensitive in the three-cell simulations. 11/20 simulations exhibited successful matings, and from the 11 matings, the **a**-cell correctly mated with the α-cell making pheromone 5/11 times (Fig 7A), which is close to the random (50%) mating discrimination score observed in experiments [19]. The p-values (Fisher’s exact test) for comparing normal sensitive cells versus supersensitive cells for mating efficiency (0.0012) and mating discrimination (0.00063) indicated significant differences between the two sets of simulations.

#### 6.2. Defective mating discrimination by supersensitive cells in five-cell simulations

To create a more competitive mating situation, we extended the three-cell simulations to five-cell mating discrimination simulations (Fig 7B). In this scenario, a single **a**-cell was surrounded on 4 sides by four α-cells (3 non-producers, and 1 producer). The **a**-cell possessing normal sensitvity was able to mate efficiently (ME = 19/20) and with almost perfect mating discrimination (MD = 18/19). When the **a**-cell was supersensitive, we observed slightly increased mating efficiency compared to the three-cell simulations (ME = 12/20); however the mating discrimination remained poor (MD = 3/12). The latter represents random mating with no regard to which α-cell is secreting α-factor (Fig 7C). The p-values obtained in Fisher’s exact test were 0.02 for ME and 9.6E-05 for MD, indicating significant differences between normal sensitive cells and supersensitive cells. In summary, we observed nearly perfect mating discrimination in wild-type cells, whereas supersensitive cells exhibited random mating discrimination. These results match experimental data from wild-type and supersensitive *sst2Δ* cells [20,45].

#### 6.3. Bar1 improves mating discrimination with background α-factor

In the five-cell mating discrimination simulations described above, normal sensitivity cells exhibited good mating discrimination in the absence of Bar1. Experimentally, it has been observed that bar1Δ cells are indeed capable of mating discrimination, but only at low mating mixture densities [19]. At high cell densities, bar1Δ cells show poor mating discrimination (nearly random), whereas Bar1+ cells are still capable of good mating discrimination. We interpreted these findings to mean that a background concentration of α-factor hindered mating discrimination. We tested this hypothesis by adding a high level of background α-factor to the five-cell mating discrimination simulations.

**Fig 8:**
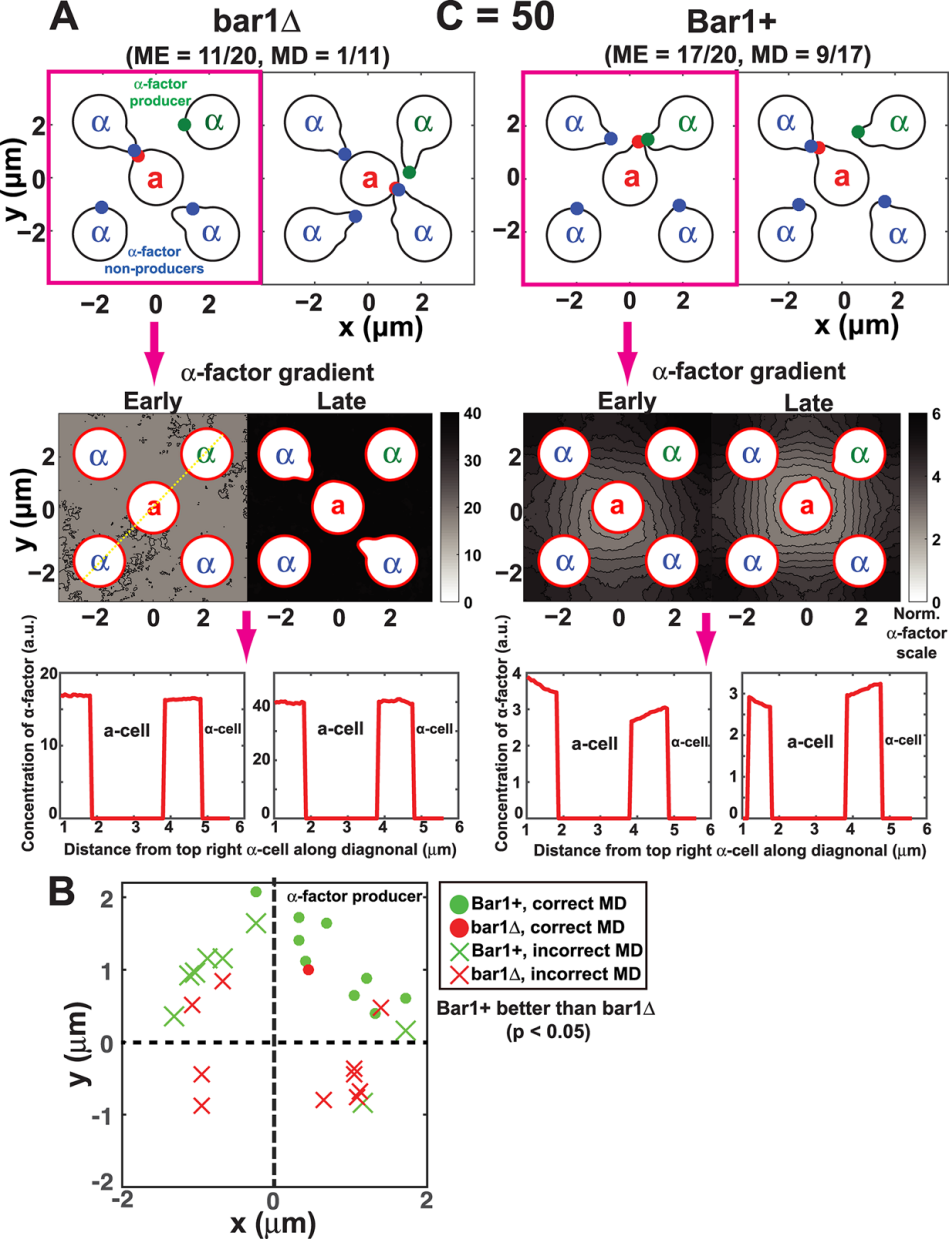
Role of Bar1 in mating discrimination with background α-factor. **(A)** Five-cell mating discrimination simulations with background α-factor in the presence and absence of Bar1. Four α-cells are arranged in a square with one **a**-cell in the center. One α-cell makes α-factor (α-factor producer, top right corner) and the other three cells do not. Background α-factor source was set to *C* = 50. There are two sample simulations for bar1Δ **a**-cells (left), and two sample simulations for Bar1+ **a**-cells (right). ME is mating efficiency, and MD is mating discrimination. Bar1 was secreted in a polarized fashion. The second row shows the α-factor profiles for one sample (left) simulation from each group. The third row shows the α-factor profiles along the top-right to bottom-left diagonal for one example (yellow dotted line). There is an early (T = 50s) and late (T = 570s) time point for each simulation with α-factor concentration indicated by the shading (color bar). Pheromone profiles show a steeper gradient in Bar1+ **a**-cell simulations; troughs represent the cell body which excludes α-factor. **(B)** Mating location plots for Bar1+ **a**-cells (green) or bar1Δ (red) in five-cell mating discrimination simulations. Each circle (correct MD) or cross (incorrect MD) symbol represents the polarisome location of the **a**-cell at the time of mating. The α-cell producing α-factor was in the top-right quadrant. The Bar1+ cells showed significantly better mating discrimination (MD) than the bar1Δ cells (p < 0.05, Fisher’s Exact Test).

With background pheromone production rate *C* = 50, the simulated mating discrimination for bar1Δ cells was 1/11 compared to 18/19 when *C* = 0. By comparison, the Bar1+ strains exhibited superior performance with MD = 9/17 when *C* = 50 (Fig 8). The p-value given by Fisher’s exact test was 0.041, indicating that mating discrimination was higher for the Bar1+ simulations. Thus, we show that Bar1 is important for both mating efficiency and mating discrimination in the presence of background α-factor. Indeed in previous experiments [19], mating discrimination for bar1Δ cells is sensitive to background α-factor concentrations, with discrimination perfect at low levels of α-factor but nearly random at high levels. Wild-type Bar1+ cells are much less sensitive to background α-factor concentrations consistent with the simulations.

Insight for this superior performance can be obtained by studying the α-factor profiles from the matings (Fig 8A, lower). Examining both an earlier and later time point, one observes that in the absence of Bar1, the level of pheromone becomes very high preventing a significant gradient from being formed. In the presence of Bar1, the background α-factor is sufficiently degraded so that a steeper gradient is created.

### 7. Estimate of the a-factor diffusion constant

We imaged mating mixes using both Bar1+ and bar1Δ cells as well as a combination of the two. We found that mating was short-range when the **a**-cells were Bar1+, i.e., both **a**- and α-cells made short projections (see S1 Text). With the bar1Δ **a**-cells, there was longer-range mating with only the **a**-cells forming longer projections. We hypothesized that degradation of α-factor by Bar1 resulting in short-range mating in the Bar1+ matings. The projection length in both simulations and experiments was defined by subtracting the initial cell radius from the distance between the center of the cell and the point that is farthest from the center on the cell membrane. The asymmetry in projections lengths in the bar1Δ matings was reminiscent of our simulations in which we varied the **a**-factor diffusion rate (Fig 2). In particular, as the **a**-factor diffusion rates became slower, the α-cell projection became shorter (and the **a**-cell projection became longer). We attributed this difference to the reduced spread of **a**-factor from its source when its diffusion constant is lower.

**Fig 9:**
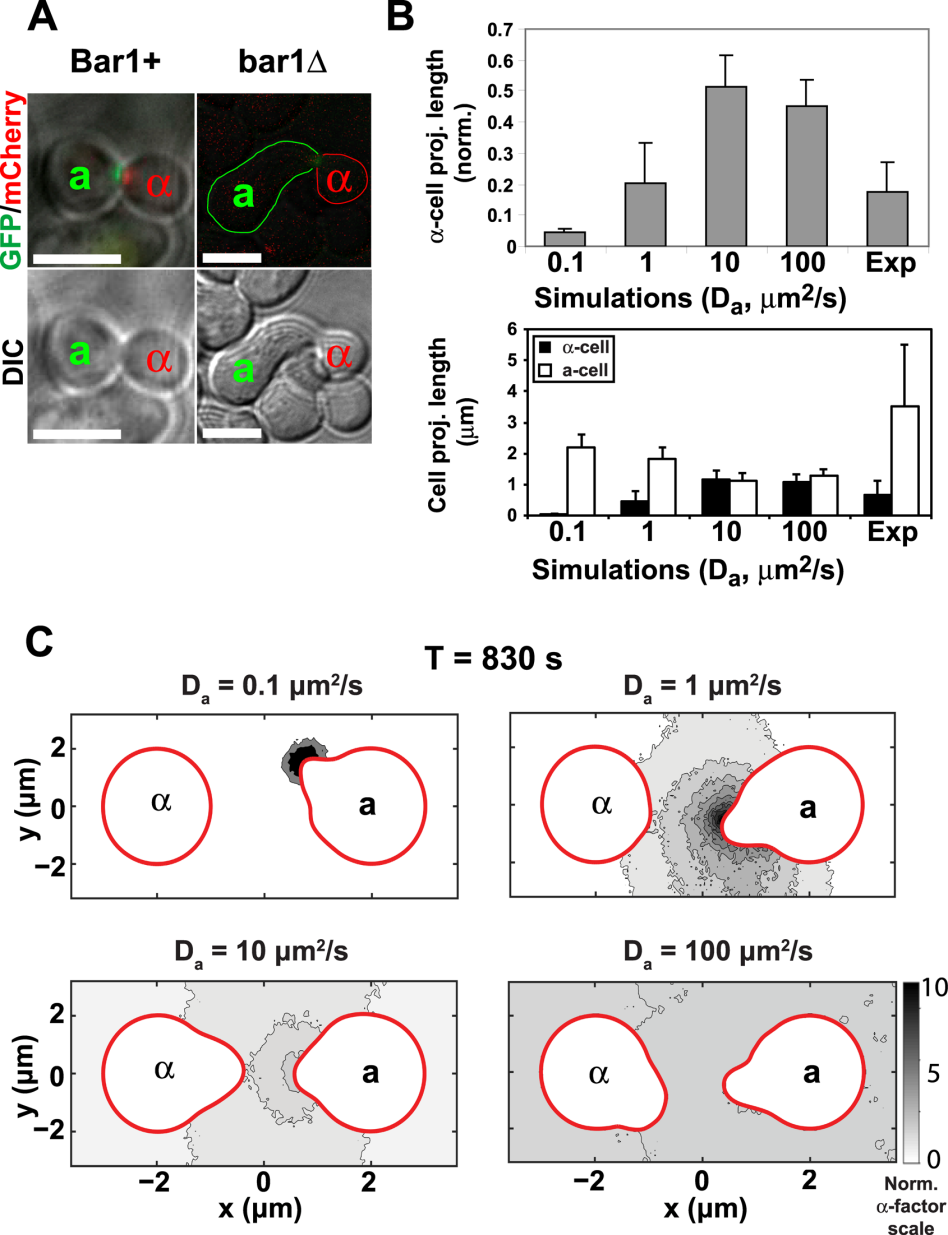
Estimating the diffusion constant of a-factor. **(A)** Projection lengths in bar1Δ versus Bar1+ **a**-cells. In the presence of Bar1, we only observed short-range matings in which both **a**-cells and α-cells possessed short projections. In the absence of Bar1 (bar1Δ matings), we observed longer projections made by the bar1Δ **a**-cells, whereas the α-cell projections remained short. The top two panels are fluorescent images of Spa2-GFP (**a**-cell) and Spa2-mCherry (α-cell) showing the adjacent/overlapping polarisomes indicating a successful mating. The bottom two panels are DIC images that depict the projection morphologies of the mating cells. Scale bars = 5 μm. **(B)** The relative projection lengths of α-cells versus **a**-cells in simulations compared to experiments. In the top bar graph, the α-cell projection length is presented as the fraction of the sum of the two projection lengths (*n* = 25 matings for Exp.); the average and standard deviation (error bars) are shown. The two-cell simulations with noise were performed as described in Fig 2 for varying α-factor diffusion values: *D*_*a*_ = 0.1, 1, 10, and 100 μm^2^/s. The average and standard deviation of the normalized α-cell projection length from 10 simulations are shown. In the bottom bar graph, the corresponding unnormalized **a**-cell and α-cell projection lengths (mean ± SD in μm) are shown. The **a**-cells in both experiments and simulations are bar1Δ. **(C)** Concentration profiles of **a**-factor for different diffusion constants. The **a**-factor distribution is color-coded using gray scale at T = 830s in one example simulation for different diffusion rates. With a diffusion constant of 0.1 μm^2^/s, **a**-factor is highly localized to its source and does not reach the mating partner. With the diffusion constant of 100 μm^2^/s, **a**-factor spreads widely and is almost homogeneous distributed.

To provide an estimate of the **a**-factor diffusion coefficient, we determined the relative length of the α-cell projection normalized by the total distance traveled by both projections, and plotted this α-cell length for both simulations and experiments in Fig 9. In the simulations we varied the **a**-factor diffusion rate from 0.1 to 100. From this comparison we estimate that the **a**-factor diffusion rate is 1 μm^2^/s.

## Discussion

In this paper we performed computer simulations of the yeast mating process for the first time. The main advance was constructing a computational framework for yeast mating which we used to explore different model structures and parameters. We reproduced qualitatively the basic mating behaviors and calculated the simulated mating efficiency. In addition, we were able to model mating competition and mating discrimination which together with mating efficiency form the three basic assays of yeast mating [19,47].

From a computational perspective, we combined modeling the shape of the cell using a moving boundary technique with the extracellular diffusion of the pheromone ligands with a previously described minimal model of pheromone-induced cell polarity. The simulations were CPU intensive because of the multiple time-scales, the evolution of the level set function over the computational domain, and the calculation of the velocity field. Overall the simulation time depended on the number of cells, time step size, length of simulation, and α-factor diffusion rate.

We examined for the first time the coupling among ligand secretion, ligand diffusion, and ligand-induced receptor activation which revealed new cell-cell interaction dynamics that could not be captured in single-cell simulations. We identified key factors that contributed to the efficiency and robustness of mating. First polarized secretion of mating pheromone resulted in higher mating efficiency than isotropic secretion. This finding is consistent with experimental data in which **a**-factor secretion through the Ste6 transporter is highly polarized [48]. It is likely that α-factor is secreted in a polarized fashion given the polarization of the secretory pathway during mating [22].

A second critical factor is the proper modulation of the sensitivity of the system. In experimental matings, strains that are “supersensitive” show considerably reduced mating efficiency and mating discrimination because they are unable to determine the pheromone gradient direction. By increasing the value of the parameter *β*_*1*_ in Eq (3), we were able to mimic the supersensitive phenotype, and the resulting mating simulations were defective.

Finally, the presence of Bar1 helped cells to mate in the presence of background α-factor. Bar1 has been implicated to play an important role in modulating the pheromone dynamics [38,39]. Our results are consistent with the conclusions in [38] that Bar1 helps to shape the α-factor gradient for optimal mating. More specifically, both results show that Bar1 can create an α-factor sink that amplifies the α-factor gradient promoting gradient-sensing. The simulations in this work incorporated stochastic effects, a generic description of intracellular signaling that drives the cell membrane, and polarized secretion of both pheromone and Bar1.

There are important limitations to this study. First, we did not attempt to present a detailed quantitative portrait of the mating process with mechanistic reactions. We employed a generic model of yeast cell polarity with a small number of variables for computational efficiency and to facilitate parameter exploration. Second, we employed a quasi-steady-state approximation of α-factor spatial dynamics, although we provide simulation data that this choice does not affect the basic results. Third, we employed mechanisms that only crudely approximate physical reality such as ligand normalization. Fourth, we have not attempted to fit the parameters to actual mating data; rather our approach was to test multiple parameters values to qualitatively explore different scenarios. We thus achieved our goal of constructing a computational framework that is capable of generating realistic-looking responses and reproducing basic behaviors.

From a technical standpoint, one important future challenge is speeding up the simulations so that the boundary velocity can be reduced to a more realistic value. One possibility is to employ a quasi-steady-state approximation for the fast α-factor dynamics. For the model with multiple cells, each cell would be assigned with a level set function and a velocity field in our framework, and so there is the potential to improve the efficiency by performing parallel computation for different level set functions or representing all cells by one level set function with a mixed velocity field. Current simulations are all restricted to two-dimensional space. Theoretically it is feasible to extend this framework into three dimensions, although the computation could be very expensive because the computational cost increases exponentially with respect to dimensionality. In addition, experimentally the mating reaction occurs on a surface (i.e paper filter) which is effectively two-dimensional [17].

Importantly this research helps to identify the key processes to focus on for future work. The generic framework is easily extended, and we can incorporate more sophisticated and detailed mechanistic models. Because of the absence of mechanistic details, the models in this work can be thought of as “general mating models”, providing a generic description of gradient tracking informed by the yeast mating system. For example, we plan to replace the normalized *f* term with pheromone-induced Bar1. In the future, an important goal is to replace the generic terms with more mechanistic terms.

With a more realistic mechanistic model of pheromone-induced cell polarity, we could attempt to simulate the mating defects of a variety of mutants. Numerous mutants have been isolated that affect mating efficiency and discrimination including *fus1Δ*, *spa2Δ*, etc. [49]. One goal would be to reproduce these mating phenotypes at a quantitative level; another goal would be to predict novel mutants that may affect mating.

## Methods

### 1. Details of mathematical model

An overview of the model including model equations is presented in the main text in Section 1. Here we present additional details.

#### 1.1. Pheromone source function

The ligand source *S*_*α*_(*x*, *t*) is a uniform or a smooth and localized function defined on the whole domain but with support only on the cell membrane. For the latter case, we approximate this source function by a Gaussian distribution in terms of the level set function *Φ* and the polarized species *u*_2_, $S_{\alpha }=\frac{1000}{\sqrt{2\pi }}\text{exp}\left(-100\phi^{2}-20{\left(1-\frac{u_{2}}{u_{2}{}_{\text{max}}}\right)}^{2}\right)$ where *u*_2max_ is the concentration of *u*_2_ at the center of the polarization region. This function represents the polarized source, whereas the isotropic pheromone source function does not have any dependence on *u*_2_, $S_{\alpha }=\frac{1000}{\sqrt{2\pi }}\text{exp}\left(-100\phi^{2}\right)$.

#### 1.2. Model parameters

We used the standard parameters described in previous work that were slightly modified [41]. The default parameters are given in the S1 Text along with simulation results from an alternative parameter set.

#### 1.3. Geometry of initial mating arrangement

For mating of two cells of different types, we set up a 2D rectangular domain [−3.6, 3.6] × [−1.6, 1.6] with one cell centered at (-2, 0). and the other centered at (2, 0). Each cell is initially represented by a circle of radius 1. For mating of three cells, the one **a**-cell is centered at (-2, 0), one α-cell centered at (1, 2) and the other centered at (1, -2) on a rectangle [−3.6, 3.6] × [−2.8, 2.8]. For the five-cell mating discrimination the **a**-cell is located at (0, 0), and the 4 α-cells are located at (±2, ±2) on a square [−3.72, 3.72] × [−3.72, 3.72].

#### 1.4. Definition of polarisome

We defined the polarisome to be the center of the minimum-length interval which contains an amount of *u*_*2*_ beyond a threshold *τ* along the cell membrane, which captures the peak of *u*_*2*_. More specifically, given 0 < *τ* < 1, let Θ_*τ*_ to be the set of intervals [*a*, *b*] satisfying $\int_{a}^{b}u_{2}\;d\theta \geq \tau \int_{S}u_{2}\;d\theta$; then the polarisome is (a*+b*)/2 where [a*, b*] is the interval with smallest b*−a* in Θ_*τ*_. In our simulations, *τ* = 0.4.

#### 1.5. Definition of a successful mating

Two cells of opposite mating type successfully mate if the projections are on average growing toward one another, and the minimum distance between their respective polarisomes is less than the distance threshold 0.04 (the mesh size).

### 2. Numerical methods

The evolution of the level set function *Φ* is governed by a Hamilton-Jacobi equation

$$\phi_{t}\left(x,t\right)+V\left|\nabla \phi \left(x,t\right)\right|=0,\;x\in D,$$

in which the velocity field *V* is defined in Eq (6). More information on how the boundary conditions are imposed on the computational grid as well as other technical details can be found in the S1 Text.

The time step is set to be 4 × 10^−4^ for extracellular pheromone, and 0.01 for membrane-associated dynamics.

The simulations were performed with the authors’ original MATLAB codes, and they can be provided by the authors upon request.

## Supporting Information

S1 Text Supporting information and figures. There are 7 sections and 14 figures (A–N) in the S1 Text.
(DOCX)
