# Supplementary material for: Modelling of Yeast Mating Reveals Robustness Strategies for Cell-Cell Interactions
Source: PLoS Comput Biol. 2016 Jul 12;12(7):e1004988. doi: 10.1371/journal.pcbi.1004988 (PMC4942089; doi:10.1371/journal.pcbi.1004988)
Supplement: S1 Text — There are 7 sections and 14 figures (A–N) in the S1 Text. (DOCX) [file pcbi.1004988.s001.docx]

**S1 Text: Supporting Information**

Modelling of Yeast Mating Reveal Robustness Strategies for Cell-Cell Interactions

Weitao Chen, Qing Nie, Tau-Mu Yi, Ching-Shan Chou

**Table of Contents:**

**S1. Model parameters**

**S1.1.** Default model parameters.

**S1.2.** Investigating a different set of parameter values.

**Figure A.** Two-cell simulations using the NPF-PF parameter set.

**S1.3.** Investigating mating behavior in uniform pheromone conditions.

**Figure B.** Simulations of mating in uniform background pheromone.

**S2. Quasi-steady-state for pheromone in the multi-scale model**

**S3. Normalization of pheromone provides computational benefits**

**Figure C.** Comparison of noise-free mating simulations which were run to quasi-steady state for pheromone (top), and for a fixed number of time steps (bottom).

**S4. Comparing constant and varying surface area simulations**

**S4.1.** Dilution effect on single cell simulations.

**Figure D.** Single-cell simulations with the dilution term.

**S4.2.** Dilution effect on two-cell mating.

**Figure E.** Two-cell mating simulations with the dilution term in the absence and presence of noise.

**S5. Details of simulations with level set methods**

**S5.1.** Representation of boundary on grid.

**S5.2.** Velocity filtering in noisy simulations.

**S6. Snapshots of simulations under different mating conditions (Figs. F to M)**

**S7. Mating cell mixtures containing Bar1+ a-cells**

**Figure N.** Snapshots of mating between Bar1+ **a**-cells and α-cells.

**S1. Model parameters**

Our generic model simplifies the polarization process into a two-module model incorporating Gβγ and Cdc42 signaling cycles. The state variables in the generic model are dimensionless, divided by the steady-state concentration of each species. There is a close correspondence between the generic model and our previously published mechanistic spatial model of the yeast mating system in which the state variables have units of concentration.

There are multiple feedback loops in the model. The negative feedback loop in the first module (Eq. (3)) approximates the negative feedback by receptor downregulation [1]; the negative feedback in the second module (Eq. (5)) represents the phosphorylation of Cdc24 by Cla4 which is activated by Cdc42 [2]. The positive feedback in the first module approximates the transcriptional induction of Gβγ as well as receptor [3]; the positive feedback in the second module represents the action of Bem1 which binds to active Cdc42 and which in turn recruits Cdc24 (Cdc42 activator) to the membrane [4].

The Hill-term parameters (*q* and *h*) represent the cooperative interactions made by Gβγ and Cdc42. Both are signaling scaffold proteins that bind numerous partners resulting in cooperative interactions. In previous work [5,6], we have shown that high values of *q* and *h* are necessary for a polarized response to a spatial gradient, and setting them to lower values such as 1 will curtail polarization. The fact that both Gβγ and Cdc42 are necessary for the formation of large signaling complexes such as the polarisome justifies values as large as 8 and 10, which are used in our generic model to establish cell polarization.

*S1.1. Default model parameters*

The default parameters are as follows. The units for the *k_i_*’s are (time unit)^-1^ in which the time unit is 100s; as described above the state variables are dimensionless, as are the Hill exponents (*q_i_* an *h_i_*) and coefficients (*β_i_* and *γ_i_*). We slightly modified the standard parameters described in previous work [2]:

$$\begin{matrix} k_{10}=1, k_{11}=10; k_{20}=1, k_{21}=10 \\ q_{1}=10, q_{2}=10; h_{1}=2, h_{2}=8 \\ \beta_{1}=0.92, \beta_{2}=0.9; \gamma_{1}=0.92, \gamma_{2}=1 \\ k_{2}=k_{3}=k_{4}=1 \end{matrix}$$

$$\begin{matrix} u_{1ss}=u_{2ss}=k_{1ss}=k_{2ss}=1 \\ D=0.0001\text{ }\mu\text{m}^{2}\text{/s} \end{matrix}$$

Initial cell geometry: 1 μm radius circle (2D).

Initial conditions:

$$u_{1}^{0}=1, v_{1}^{0}=1, u_{2}^{0}=1, v_{2}^{0}=1$$

*S1.2. Investigating a different set of parameter values*

We wished to test the mating with a different parameter set from the default parameter set described above. The default parameter set corresponds to the PF-PF version of the model described in [2] in which there are two stages both containing positive feedback (PF). We also tested an NPF-PF version of the model in which the first stage lacks positive feedback (No Positive Feedback or NPF) and acts as a filter for the positive feedback in the second stage. The key change is setting *k_10_* = 10 (instead of 1), and setting *k_11_* = 0 (instead of 10); the latter parameter influences the strength of the positive feedback. The full parameter set is shown as follows (the number in parentheses is the default parameter value):

$$\begin{matrix} k_{10}=10 (1), k_{11}=0 (10); k_{20}=1, k_{21}=10 \\ q_{1}=100 (10), q_{2}=10; h_{1}=2, h_{2}=8 \\ \beta_{1}=0.92, \beta_{2}=0.9; \gamma_{1}=0.92, \gamma_{2}=1 \\ k_{2}=k_{3}=k_{4}=1 \end{matrix}$$

We reproduced Figure 2 in the main text using this new parameter set which shows the effects of different levels of external (*κ_1_*) and internal (*κ_2_*) noise on the two-cell mating simulations (Fig. A). Overall the results were similar although the NPF-PF model showed more ability to mate at higher internal and external noise levels.

| $\boldsymbol{\kappa}_{\mathbf{1}}$ | $\kappa_{2}=0$ | $\kappa_{2}=3$ | $\kappa_{2}=5$ |
| --- | --- | --- | --- |
| $\kappa_{1}=0$ | 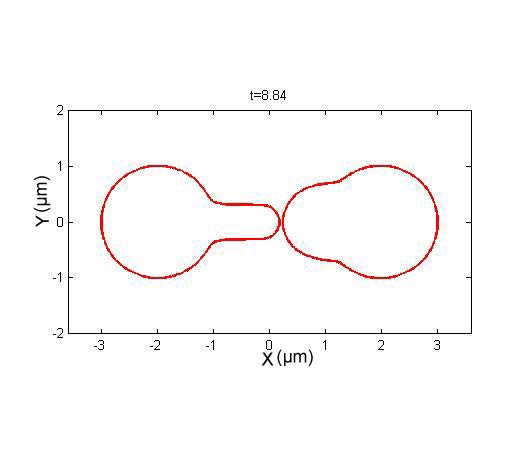 | 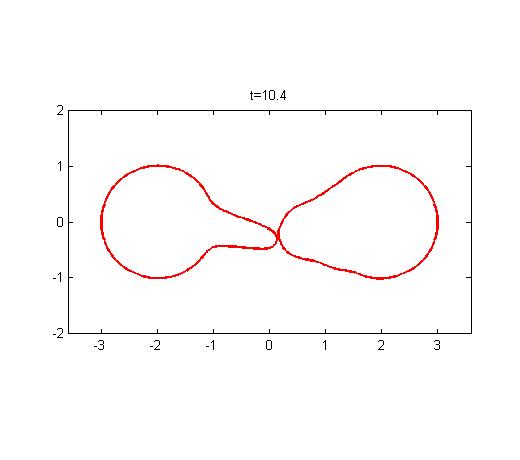 | 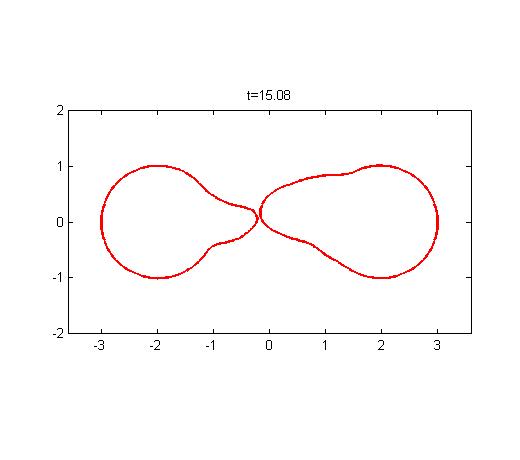 |
| $\kappa_{1}=5$ | 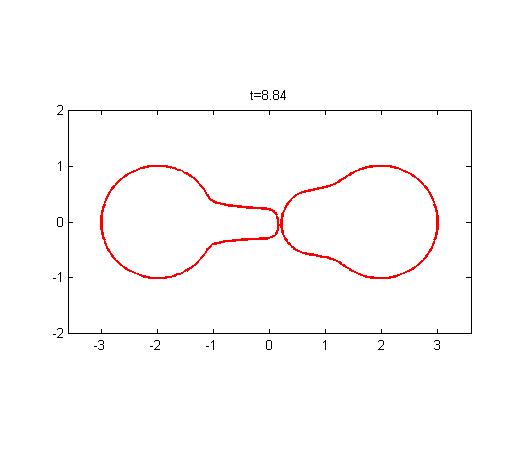 | 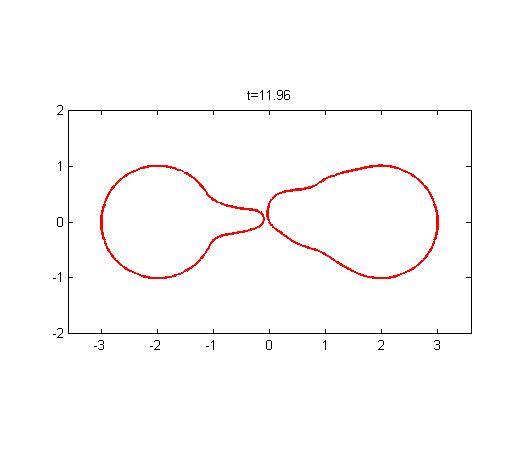 | 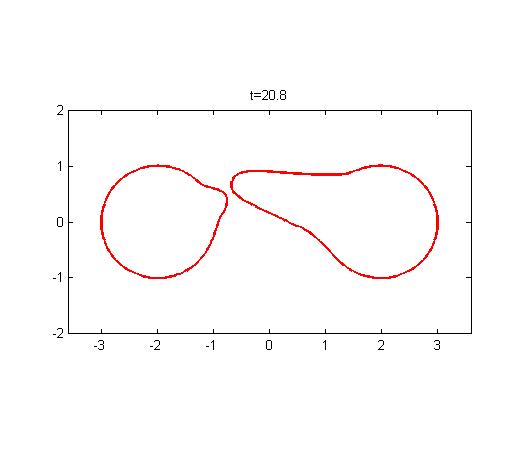 |
| $\kappa_{1}=10$ | 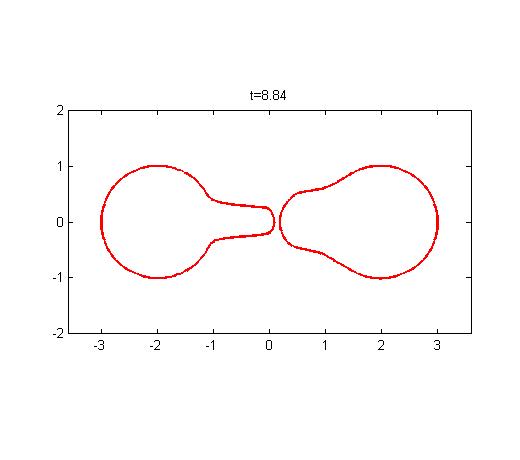 | 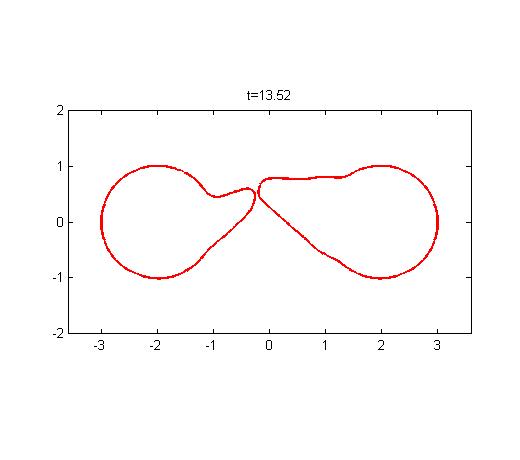 | 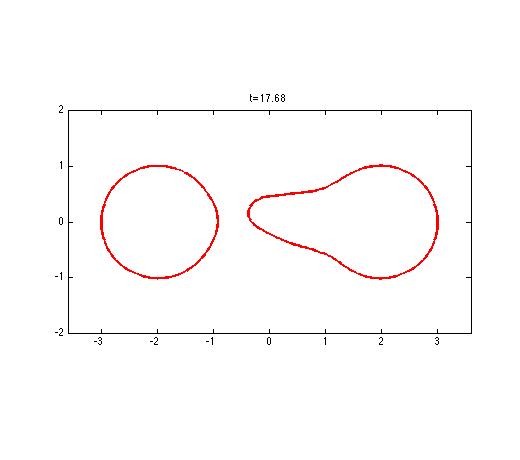 |

**Figure A.** Two-cell simulations using the NPF-PF parameter set were run for a range of values of external (*κ_1_*) and internal (*κ_2_*) noise (0, 5, 10 for *κ_1_*; 0, 3, 5 for *κ_2_*). An example simulation is shown for each specified pair of values (*κ_1_*, *κ_2_*). The α-cell is on the left, and the **a**-cell is on the right.

*S1.3. Investigating mating behavior in uniform pheromone conditions*

It is known that mating yeast can still polarize and form a mating projection in the absence of pheromone secretion when pheromone is added exogenously to create a uniform distribution. We tested the mating behavior under these uniform pheromone conditions using our stochastic model. The set-up was almost the same as the default simulation with two cells except that pheromone concentration was given as 10+10κξ, where κ=0.03 and ξ~N(0,1), in the background of the extracellular space. We performed 20 simulations, and each one was run until T = 2000 sec or the two cells touched. The mating efficiency was zero, which is consistent with the low mating efficiency observed experimentally under isotropic pheromone conditions [7]. Some typical results are shown below (Fig. B). Notice that due to the absence of a sharp gradient around the cells, the morphological changes exhibit more randomness, yielding irregular cell shapes and independent directions of growth.

**Figure B.** Simulations of mating in uniform background pheromone. Cells did not secrete pheromone, and instead there was a uniform background concentration of both pheromones at a value of 10. The simulations were run for T = 2000 seconds. The red and blue circles indicate the locations of the polarisomes.


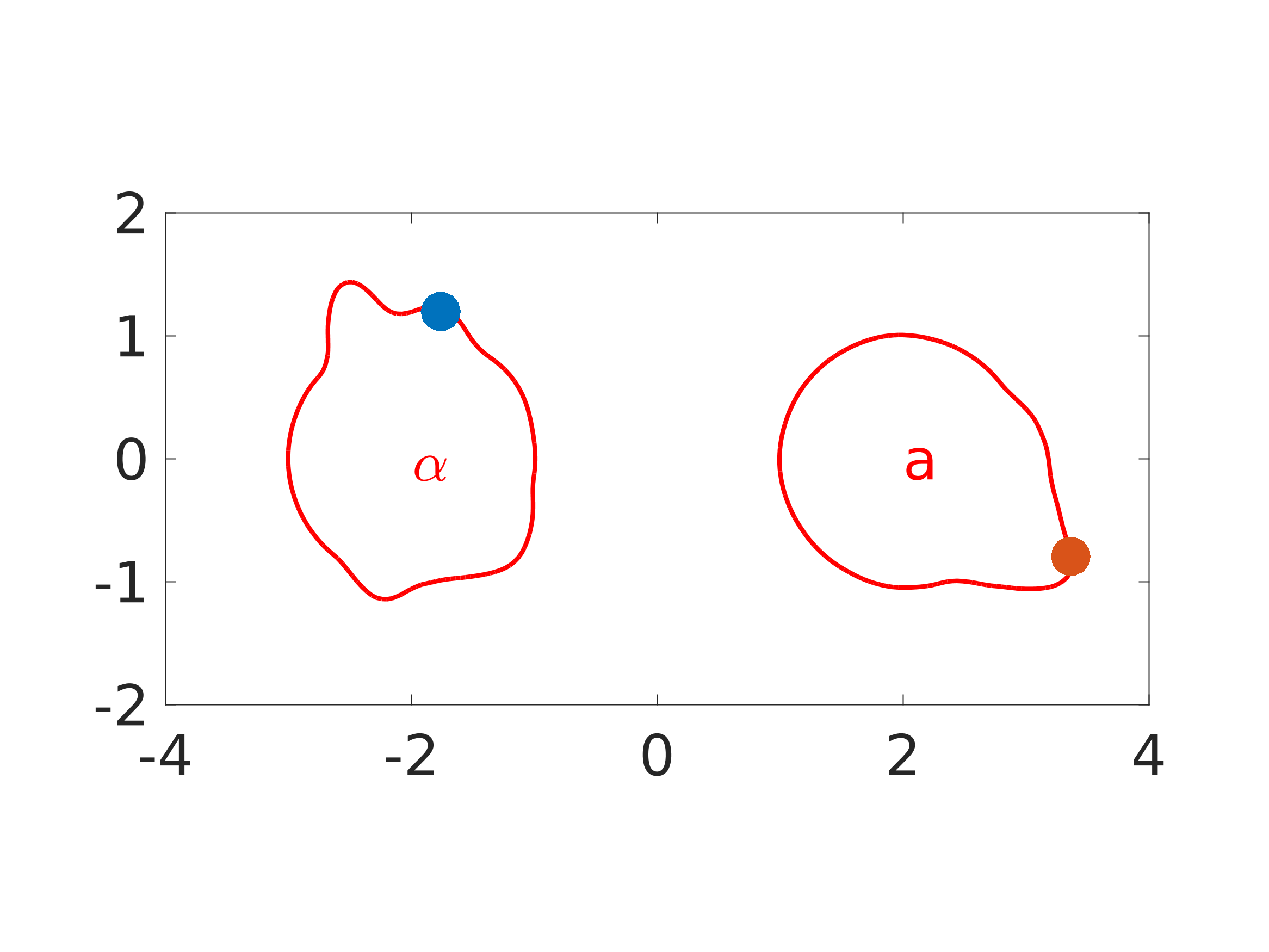

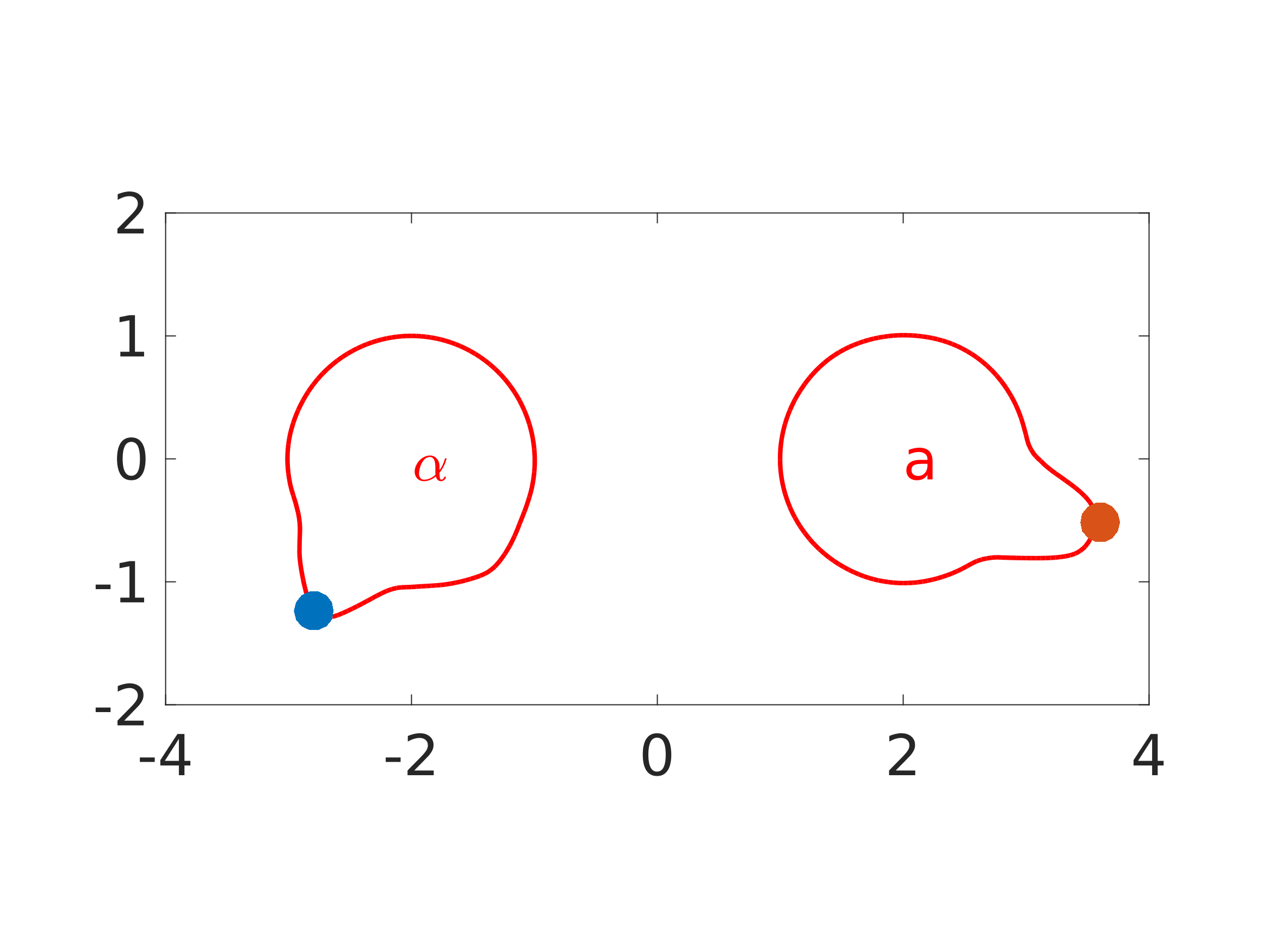


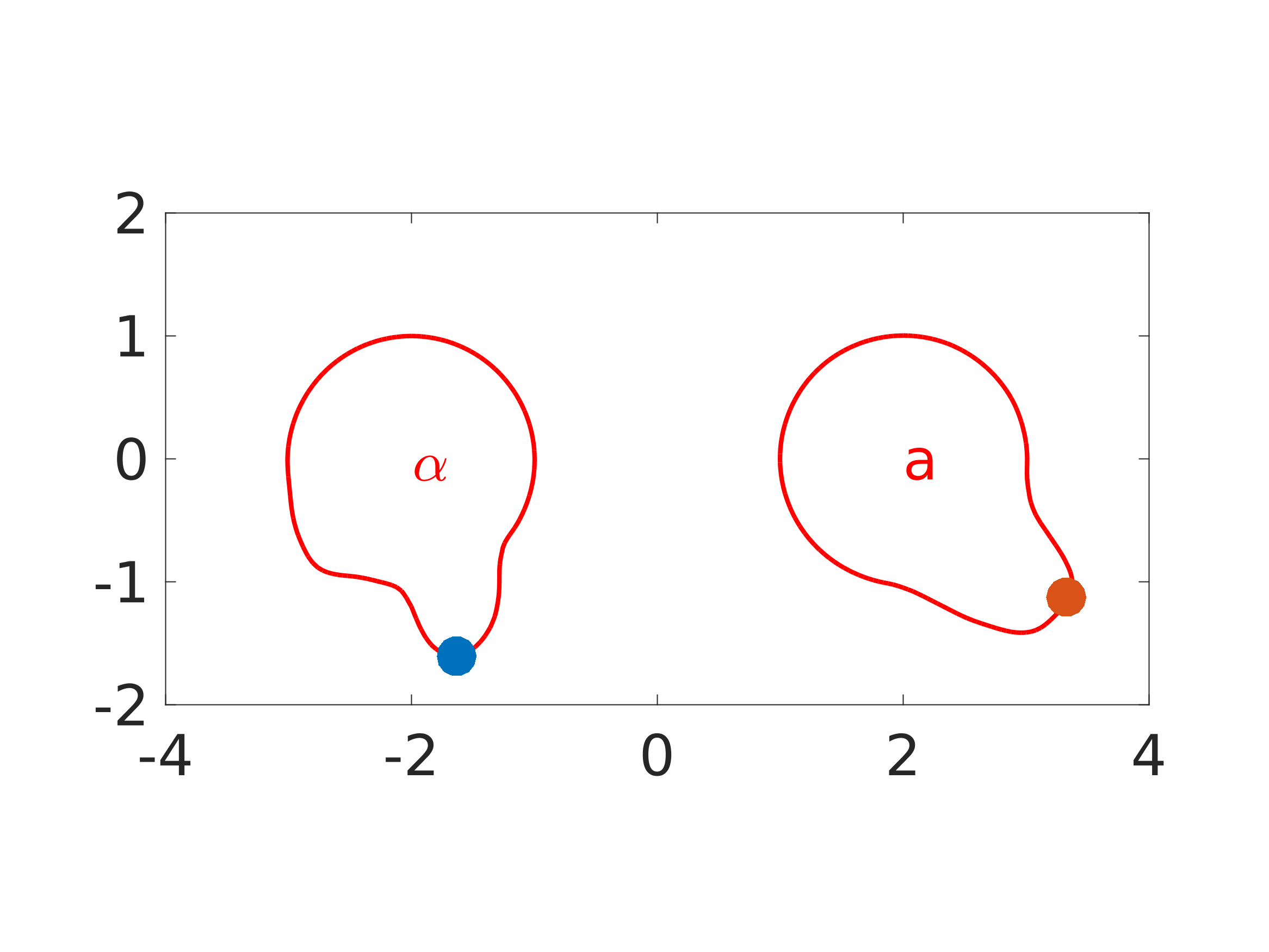

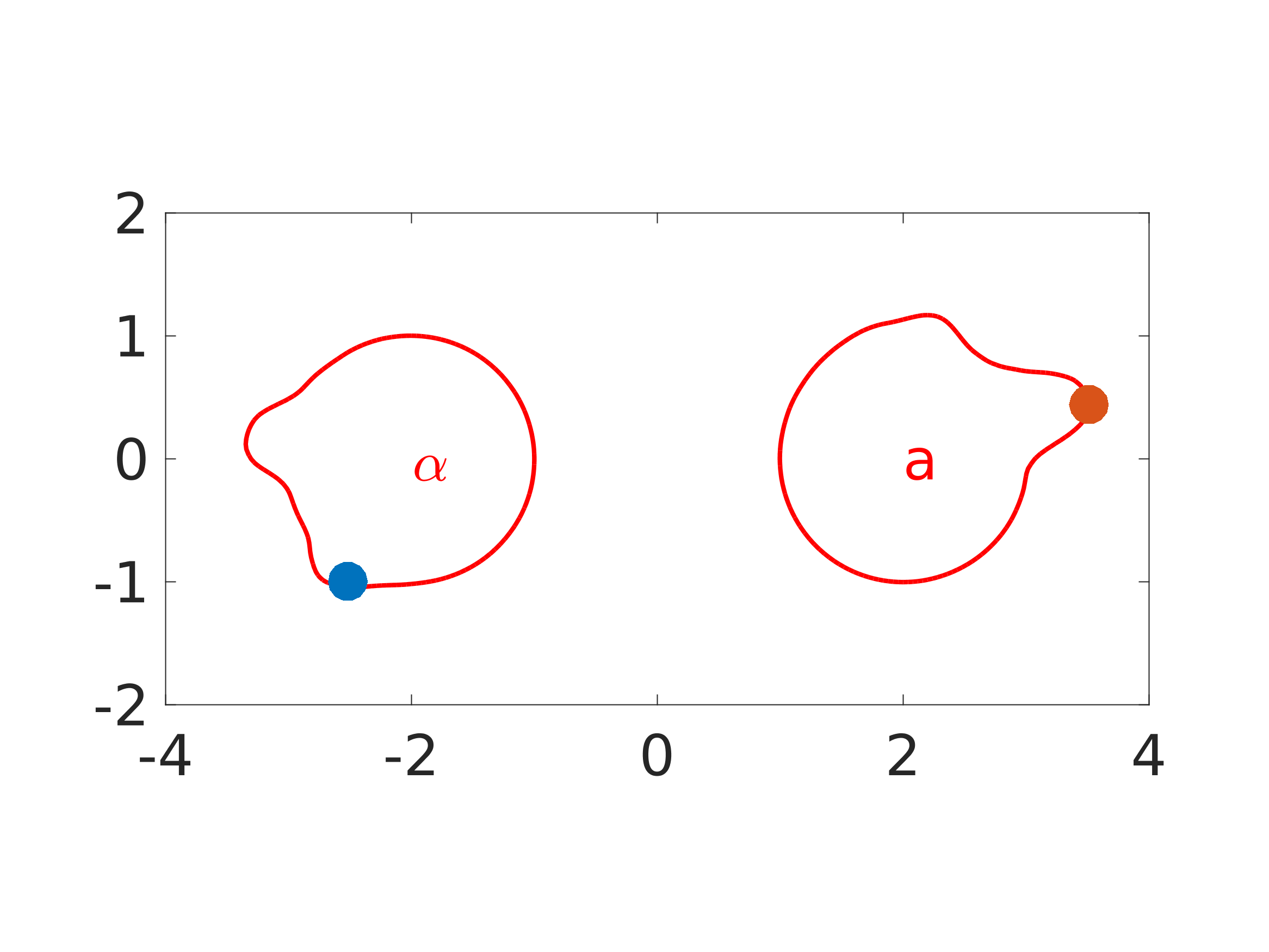


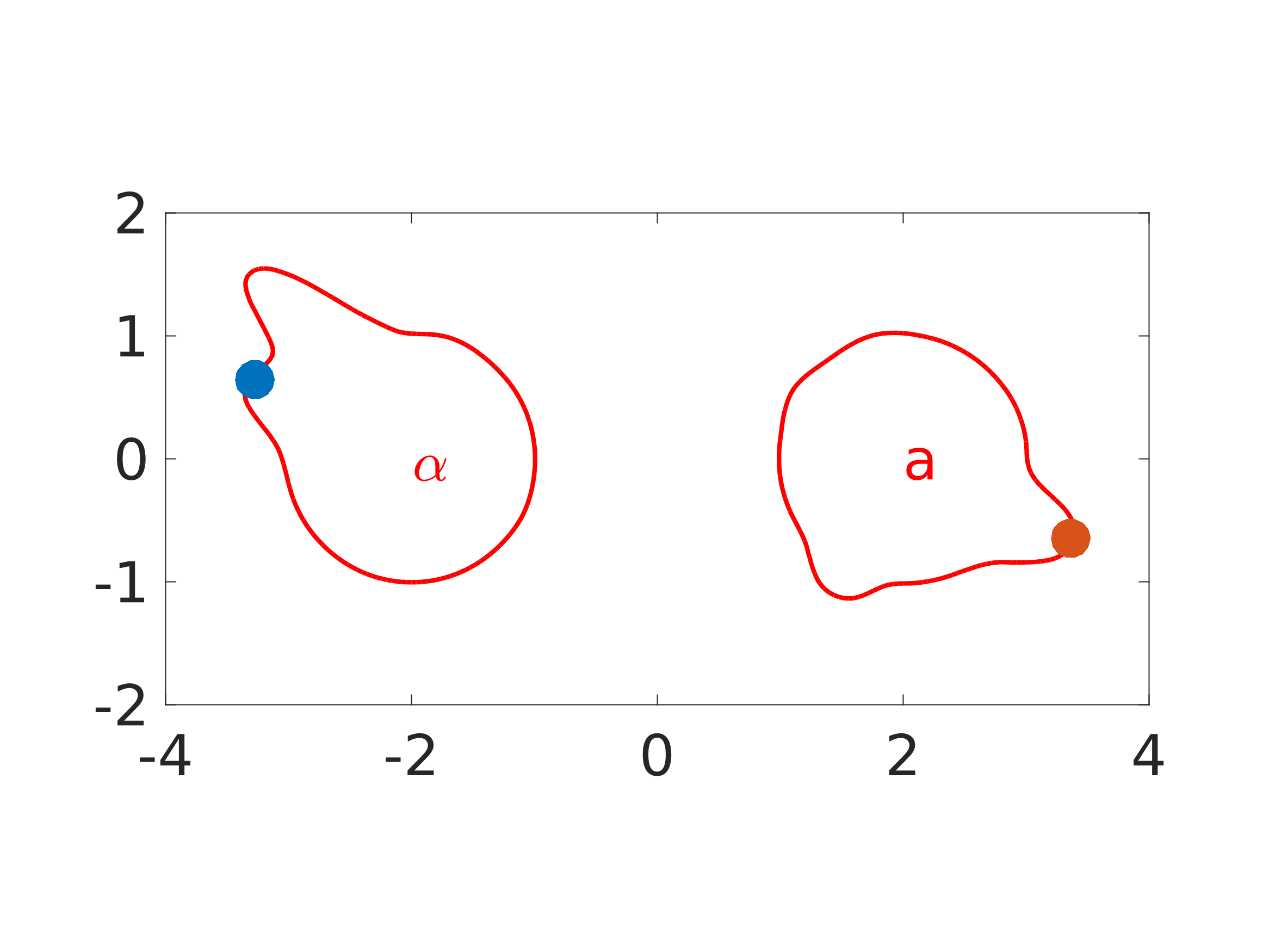

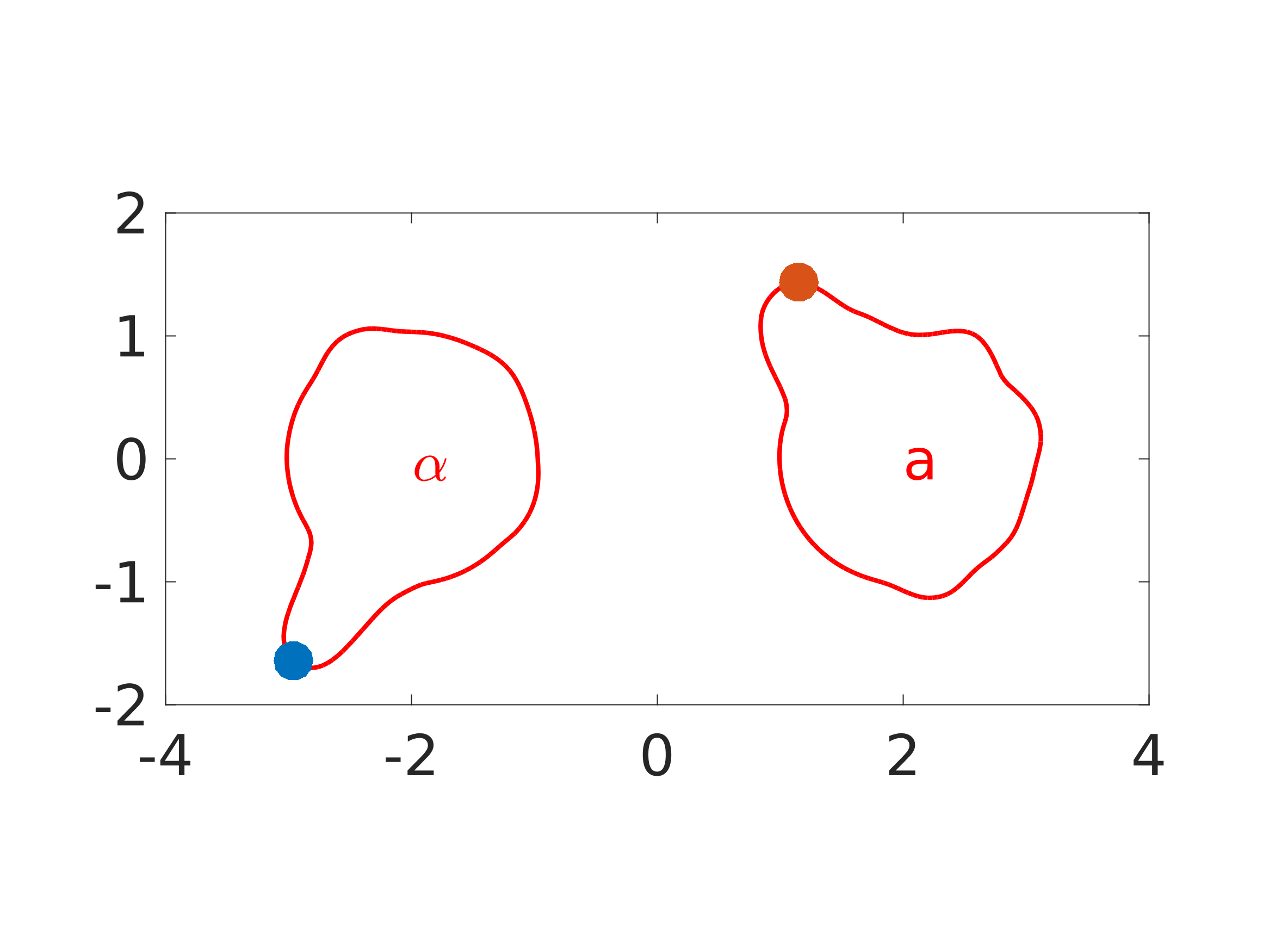


Y(μm)

X(μm)

**S2. Quasi-steady-state for pheromone in the multi-scale model**

The diffusion of pheromone (i.e. α-factor or **a**-factor) and movement of the cell boundary are at different time scales. Experimentally, extracellular diffusion is much faster (~10,000-fold) than cell growth. Because of its fast time-scale compared with cell growth, the distribution of pheromone can be approximated by solving for the quasi-steady-state. The boundaries of the pheromone diffusion include the moving cell membranes at which a no-flux boundary condition is imposed. Because we also would like to add pheromone noise, we use time marching to obtain the steady-state. For the purpose of efficiency, we apply the Crank-Nicholson method that is a semi-implicit method allowing relatively large time steps.

We specify the time unit in our simulations to be 100s. However, below we show that the quasi-steady-state for pheromone distribution is independent of the time unit, and more specifically is the same whether *t_u_* = 1s or 100s.

*2.1. Quasi-steady-state of pheromone is independent of time unit*

Consider the equation of the pheromone in extracellular space; the biological focus is on α-factor because of its faster diffusion:

$\frac{\partial f}{\partial t}=D\Delta f+S\left(x,t\right)-kf,\text{ }x\in\Omega^{ex}$. (S1)

All coefficients are associated with the time unit. However, if we consider the steady state of the above equation, it is in the form of $f=f_{s}e^{-x/\lambda}$ with the length scale $\lambda=\sqrt{D/k}$, which is independent of the time unit. This indicates that the coefficients in the equation of pheromone could be modified if the time unit is changed, but the steady-state is invariant with respect to the time unit.

We tested the idea that the pheromone quasi-steady-state is insensitive to the time unit with a simple example. Two simulations were implemented with different time units: one with a time unit of 1 second and the other with a time unit 100 seconds. Both simulations were run until the stationary state was achieved. The spatial profiles of α-factor and **a**-factor from both simulations were nearly identical (i.e. within numerical error; data not shown). Thus, pheromone achieves a quasi-steady-state independent of the time unit as expected. These data suggest that setting the unit of time to be 100s instead of 1s would not alter the pheromone distribution, making the simulations relevant for a broad range of time-scales.

Note that for the steady-state of a stochastic differential equation, changing the time unit will change the temporal frequency of noise, which affects the variance in the solutions but does not change the mean.

**S3. Normalization of pheromone provides computational benefits**

In our model, we normalize the pheromone concentration to the maximum value on the cell surface. In this manner we ensure that the proper dynamic range for gradient-sensing is maintained. Over time, the amount of pheromone in the extracellular space increases as long as pheromone production exceeds pheromone degradation by Bar1. Without normalization, the sensing mechanism which is modeled by a Hill function with fixed coefficients becomes saturated and the cell cannot distinguish front from back, i.e. which end is exposed to the higher level of pheromone. Thus, pheromone normalization allows us to use a consistent parameter set in the simulations containing more α-cells. Biologically, this normalization may represent the adjustable dynamic range mechanisms in the system designed to prevent the sensing from saturating, i.e., cells adapting to the change in environmental stimulus. In the future, we plan to explore explicit mechanisms for this sensing adaptation.

In addition, normalizing the pheromone concentration turns out to improve the computational efficiency of solving the steady state of Eq. (S1) when there is no external noise (see Section S2). Usually, achieving the steady-state by advancing in time is time-consuming. With ligand normalization, only around 2500 time steps will be needed to obtain a good approximation of the steady state (Figure C).


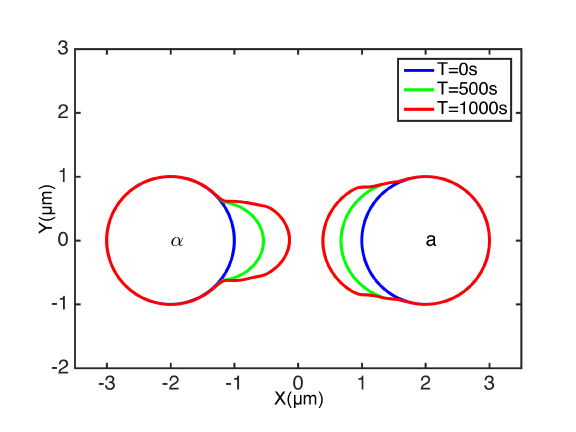


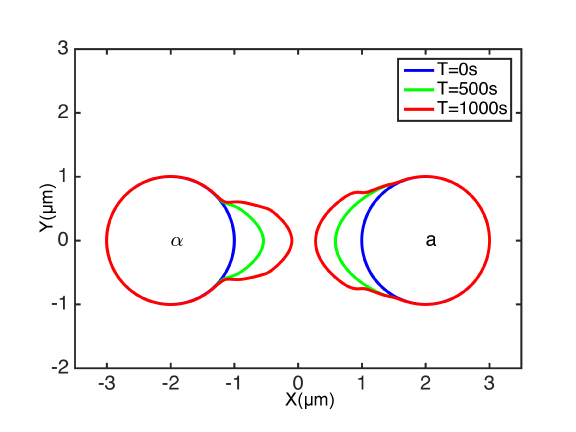


**Figure C.** Comparison of noise-free mating simulations which were run to quasi-steady-state for pheromone (top), and for a fixed number of time steps (bottom). The morphological change in time is quite similar for the two simulations.

**S4. Comparing constant and varying surface area simulations**

During the morphological change, the cell membrane surface area is expected to increase so that the concentration of membrane-associated molecules will be diluted if the number of molecules is constant. The dilution effect is described by the advection term $\nabla_{s}\cdot(uV)$ in Eq. (3) of the main text. This term, after simplification, can be written as $v_{n}\kappa u$, where $v_{n}$ is the normal component of *V*, $\kappa$ is the curvature of the membrane, and $R(u)$ represents the reaction term. In other words, the local decline in concentration due to the dilution term is related to the velocity and the curvature of the moving surface.

To numerically solve the PDE, we approximated the curvature in terms of the level set function *ϕ*. The unit normal vector to the cell membrane can be calculated as . According to its definition, $\kappa$ can be calculated as , and a central difference formula is used to calculate $\kappa$. The computation of the right hand side and the time integration are performed as described in Methods of the main text.

We then compared simulations with the dilution term to our previous simulations without it. We examined simulations of single cells as well as two-cell mating simulations both in the absence and presence of noise. Our expectation was that they would be similar based on the fact that the model contains integral feedback control that will keep the total steady-state concentration of the membrane species at a constant even with changes in the surface area.

*S4.1. Dilution effect on single-cell simulations*

**(A)**


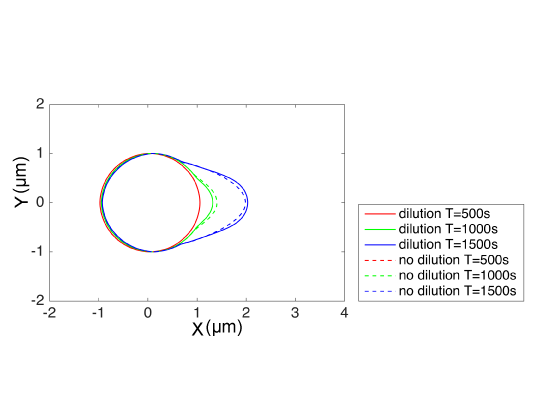


**(B)**


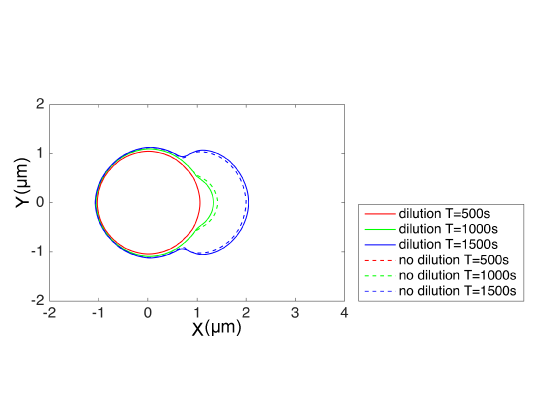


**Figure D.** Single cell simulations with the dilution term. (**A)** Mating projection formation. (**B)** Budding. Solid lines are with dilution (i.e. taking into account expanding surface area of growing cell) and dotted lines are without dilution (i.e. standard simulations).

We implemented single cell simulations with or without the dilution term and compared the cell shape at different time points. We examined growth of a mating projection or bud in the absence of noise. Growth was induced by an external cue (pheromone gradient) for the projection and an internal cue for the bud [6]. For both the projection and bud, the simulations with or without the dilution term produced nearly identical morphologies (Fig. D). Thus, in single cell simulations the dilution term did not affect the results while slowing down the simulations approximately five-fold.

*S4.2. Dilution effect on two-cell mating*

**(A)**


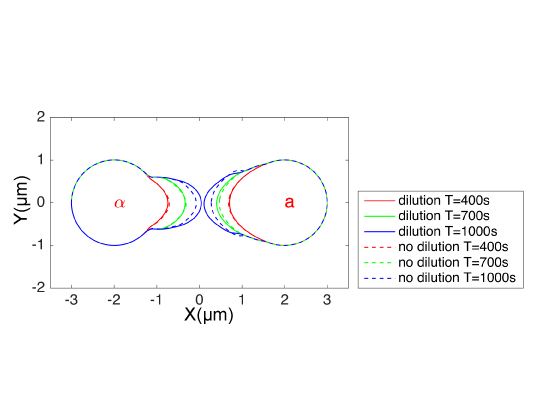


**(B)**


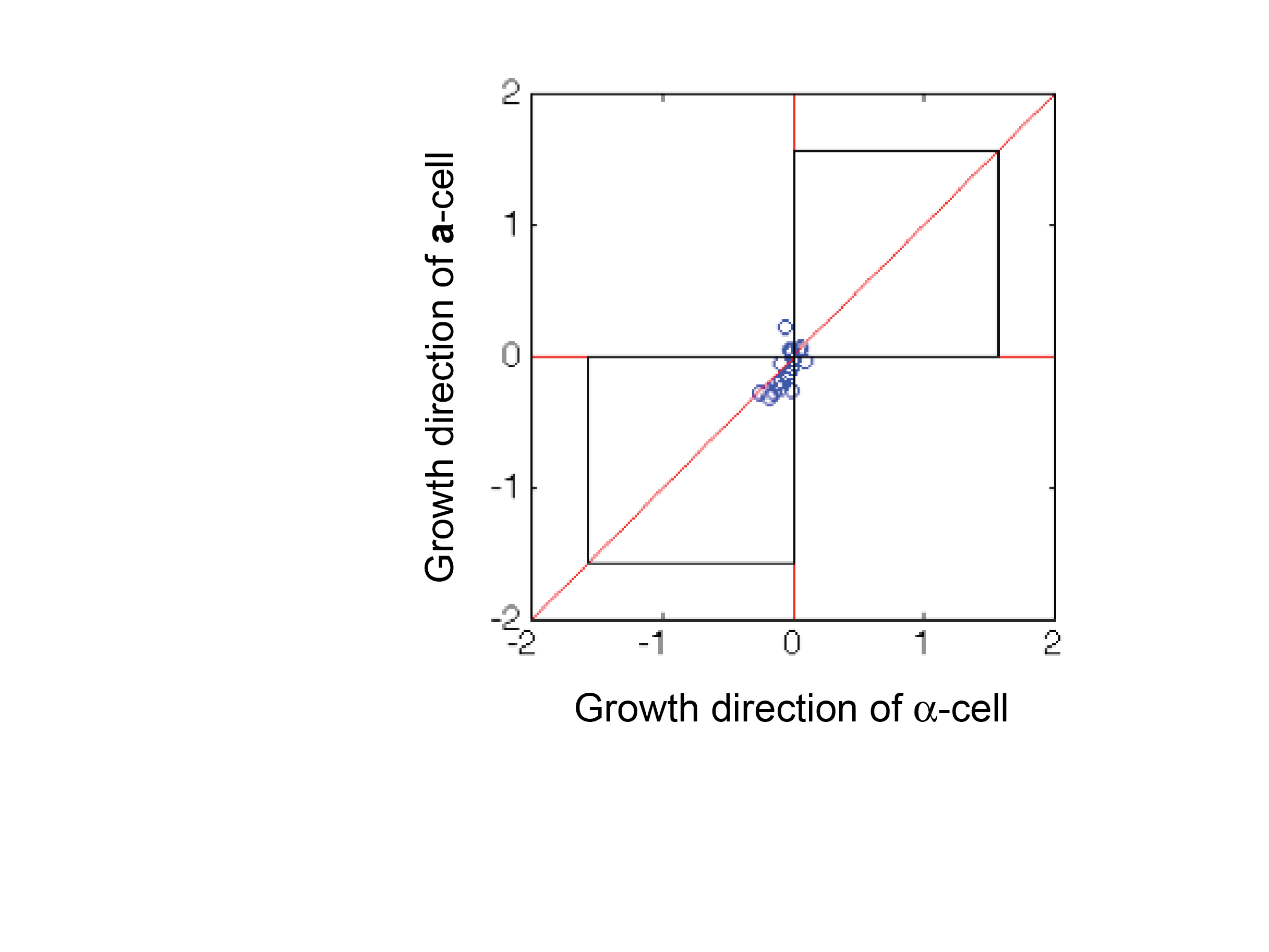


**Figure E.** Two-cell mating simulations with the dilution term in the absence and presence of noise. (**A)** Including the dilution term does not significantly change the cell morphology during noise-free mating simulations. (**B)** Direction plot of two-cell mating with the dilution term in the presence of noise. Mating is slightly more efficient with the dilution term than without, and the direction points are closer to the diagonal. The cells were bar1Δ.

We also implement the simulation with dilution effect for two-cell mating in the absence and presence of noise. Comparing the cell morphologies with or without the dilution term in the noise-free case, we find a similar behavior for the cell projection shapes of the two cells (Fig. E(A)). We then measured mating efficiency in the presence of extrinsic and intrinsic noises ($\kappa_{1}=5,\kappa_{2}=3$), and found that with the dilution effect, mating efficiency is slightly higher at 95% (19/20) versus 75% (15/20) for the default simulations without the dilution term. The direction plot shows the efficient mating with the points close to the diagonal representing projections pointing toward each other (Fig. E(B)).

Overall, including the dilution term did not drastically change the simulation results in terms of cell morphology and mating efficiency. On the other hand, the simulations of our models ran much faster without the dilution term, which is why we excluded the term in the default simulations described in the main text.

**S5. Details of simulations with level set methods**

Our model incorporates both protein dynamics on the membrane and pheromone diffusion in the extracellular space. These processes operate at different time scales; pheromone diffusion is much faster. Therefore, we used different time steps to solve the pheromone diffusion equation (Eq. (1) of main text) compared to the rest of the polarization model (Eqs. (3)-(7)).

Pheromone diffusion is described by a typical reaction-diffusion equation in a 2D domain, for which the Crank-Nicolson method is used in the simulations, with time step around 10^-4^. All simulations were run until *t* = 2000s or until cells successfully mated. Since
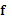
 *f* only diffuses in the extracellular region, no-flux boundary conditions are imposed along the cell membranes. Due to the irregular cell shapes, the cell membrane may intercept with the mesh at non-grid points and the normal direction of the surface is not necessarily aligned with the axes. To avoid computationally expensive interpolation, we implemented a constant extension along the normal direction by solving the stationary Hamilton-Jacobi equation [8,9],

.

Such constant extension can be obtained by iterating the time evolution problem

until its steady state is reached. Since accurate information of $f$ is needed only around the cell membrane which will be captured by first few iterations, only a couple of iterations are necessary to obtain the values on a narrow band around the cell membrane. This extension provides approximate solutions at ghost points for imposing the boundary conditions.

The dynamics of molecules on the cell membrane are updated with a much larger time step (~10^-2^) along with the level set evolution. Surface diffusion, which refers to lateral surface diffusion as a result of random motion of molecules restricted to the surface, is transformed into the equivalent operator on a 2D domain, which converts the equations for *u_1_* and *u_2_* (Eqs. (3) and (5)) into a form suitable for a finite difference scheme on a fixed mesh. The model equations (3)-(6) are discretized by a second-order central difference scheme and march in time with the second order total variation diminishing (TVD) Runge-Kutta method. The level set function, Eq. (7), is solved by the fifth order Weighted Essentially Non-Oscillatory method and a third order TVD Runge-Kutta method. Because the velocity function ***V***, determined by intracellular molecules, is only well-defined along the zero contour of *ϕ*, it is necessary to extend ***V*** into the whole domain so that it assumes constant values along directions normal to the cell membrane. This was done by solving a stationary Hamilton-Jacobi equation. The algorithm is summarized in the following:

| Algorithm: Incorporating dynamics of multiple chemical species (u_i_, v_i_ ) on the cell membrane and cell growth during yeast mating |
| --- |
| Initial condition: u_i_ = 1 and v_i_ = 1. Let ϕ_i_ be the level set function representing the i^th^ cell which is a unit circle initially.  While *t < Tcut_off* and no cells touch each other   1. Perform a constant extension of u_i_‘s and v_i_‘s along the normal direction of the corresponding ϕ_i_. Define pheromone source functions on each cell. 2. Update a- and α-factor distribution as quasi-steady state according to Eq. (1a) and (1α). 3. Update u_i_‘s, v_i_‘s as described in Eq. (2-5), and ϕ_i_‘s by defining V_i_, the velocity field to drive evolution of ϕ_i_, according to Eq. (6).   End |

*S5.1. Representation of boundary on grid*

When using the level set method to track the plasma membrane [10], the cell interface rarely intersects with the grid points, which results in a poor representation of the membrane by insufficient points if the strict equality is chosen to describe it. To deal with that, we define points that satisfy to be the plasma membrane, where $\delta$ is a constant multiple of the mesh size. Hence, the cell membrane is tracked as a narrow band. In our simulations, we let $\delta$ be equal to the mesh size.

During the simulations, cell surface area increased because of projection growth. However, for computational efficiency, we did not take into account the dilution effect. Relaxing this constraint did not alter the simulation results in a significant fashion (see Section S4).

*S5.2. Velocity filtering in noisy simulations*

To smooth spikes in the velocity function caused by noise, we apply two filtering strategies commonly used in signal processing, the median filter and the Savitzky-Golay smoothing filter. These filters represent unmodeled biochemical processes that produce spatial and temporal averaging. For the median filtering, the velocity at a given point is replaced by the median of neighboring values within a given window size (31 points). For the Savitzky-Golay smoothing filter, the velocity is smoothed by convolution with its adjacent (24) data points.

**S6. Snapshots of simulations under mating different conditions**

We provide several examples of simulations under different biological conditions discussed in the Results section of the main text. Simulations were carried out on a Mac Pro with a 3.5 GHz 6-Core Intel Xeon E5 processor. The computational time for a default two-cell mating was approximately 8 hours.


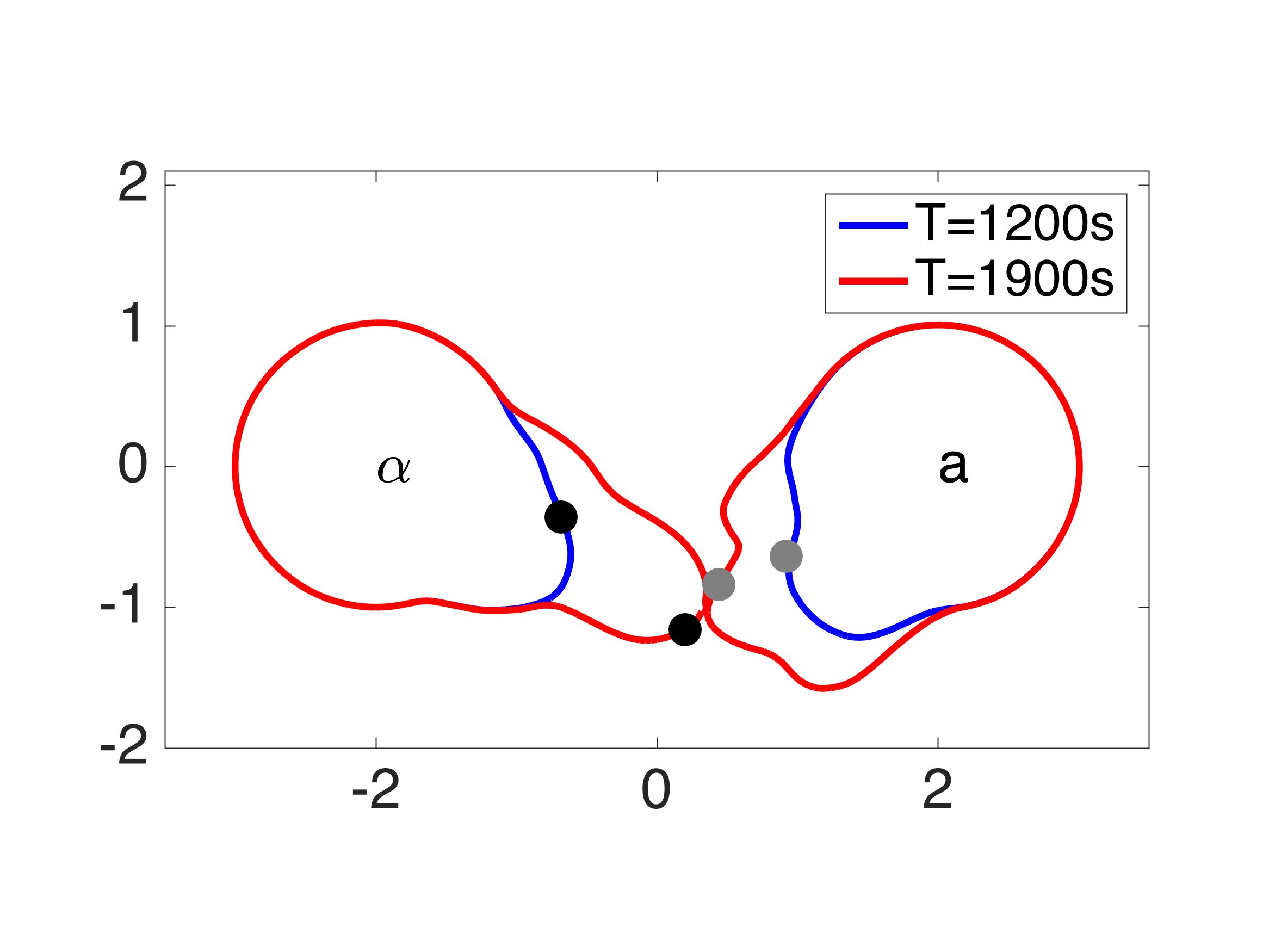

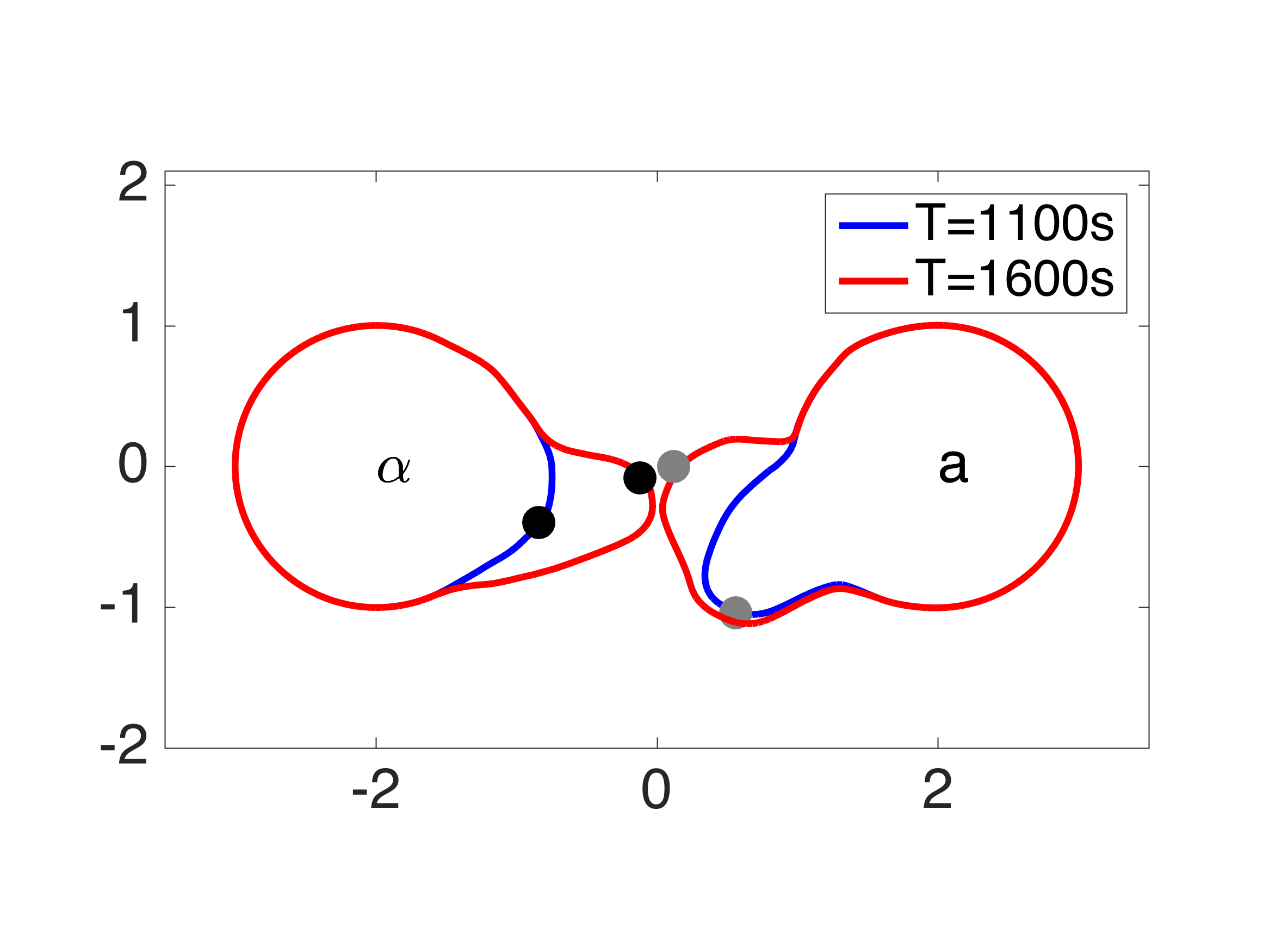


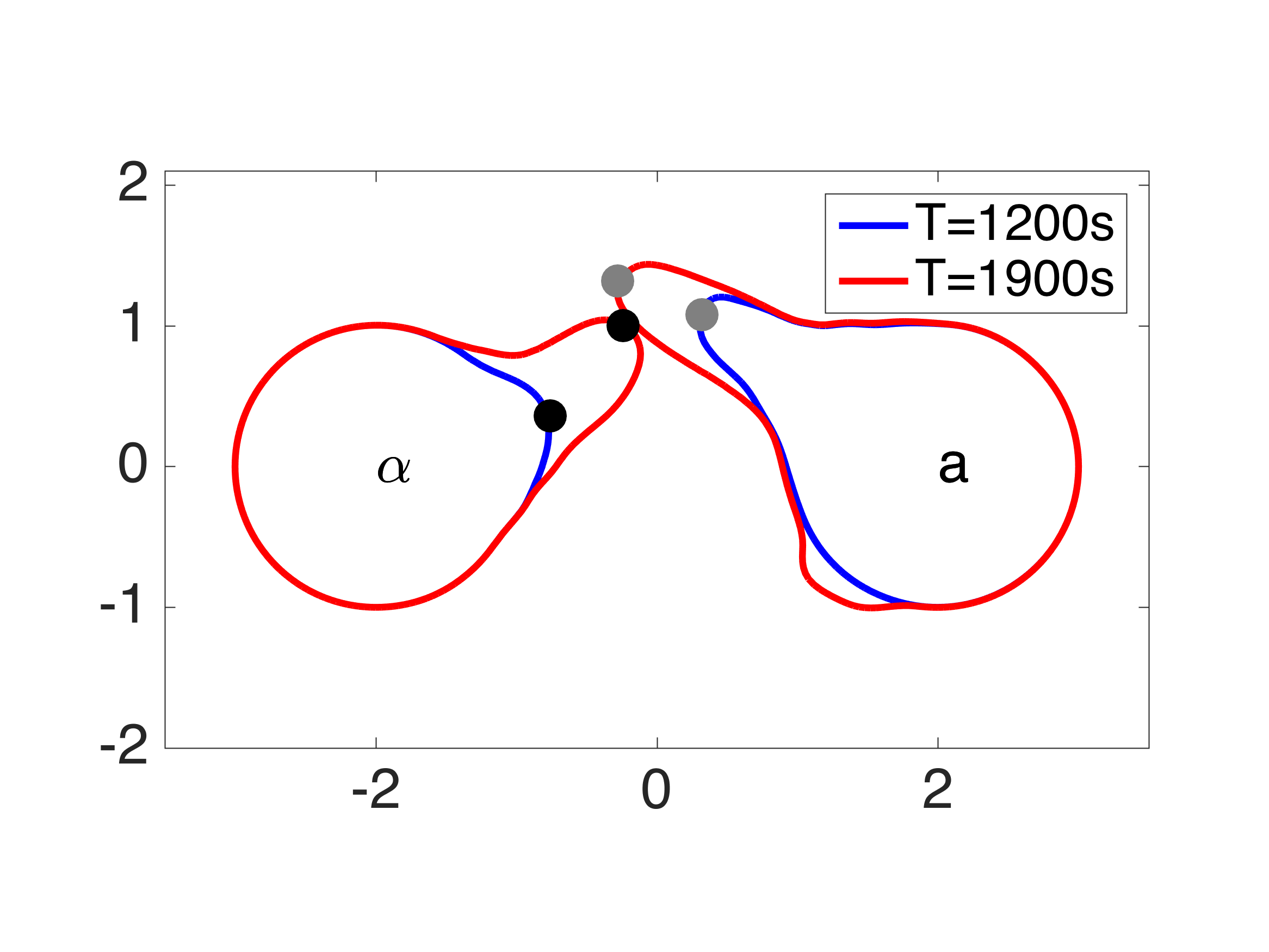

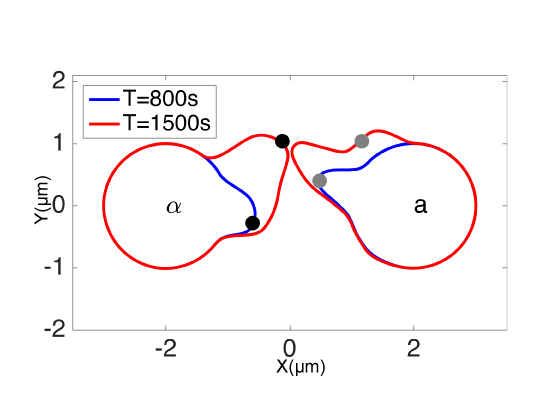


**Figure F.** Examples of two-cell mating under default condition. Filled circles represent positions of polarisomes.


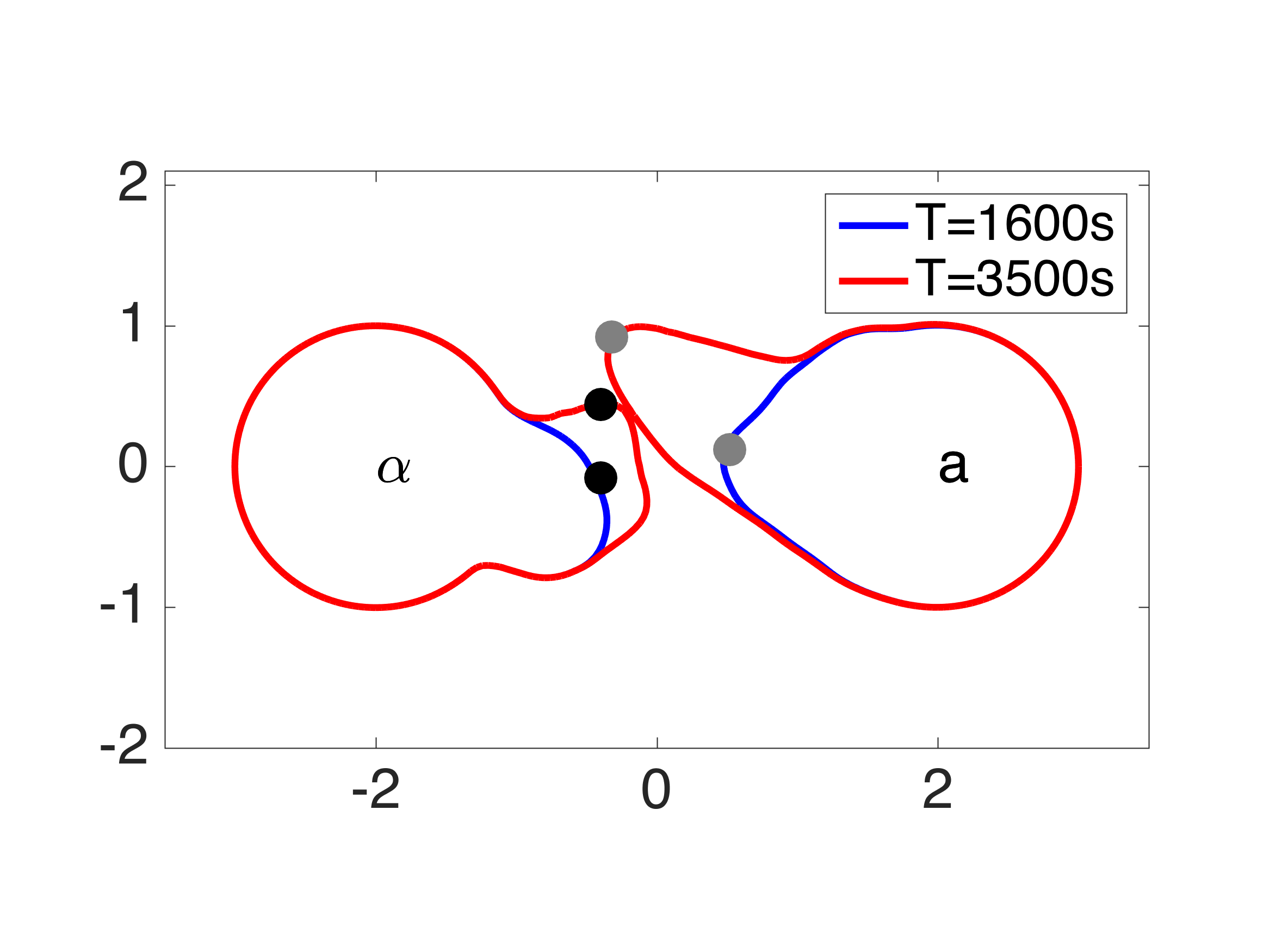

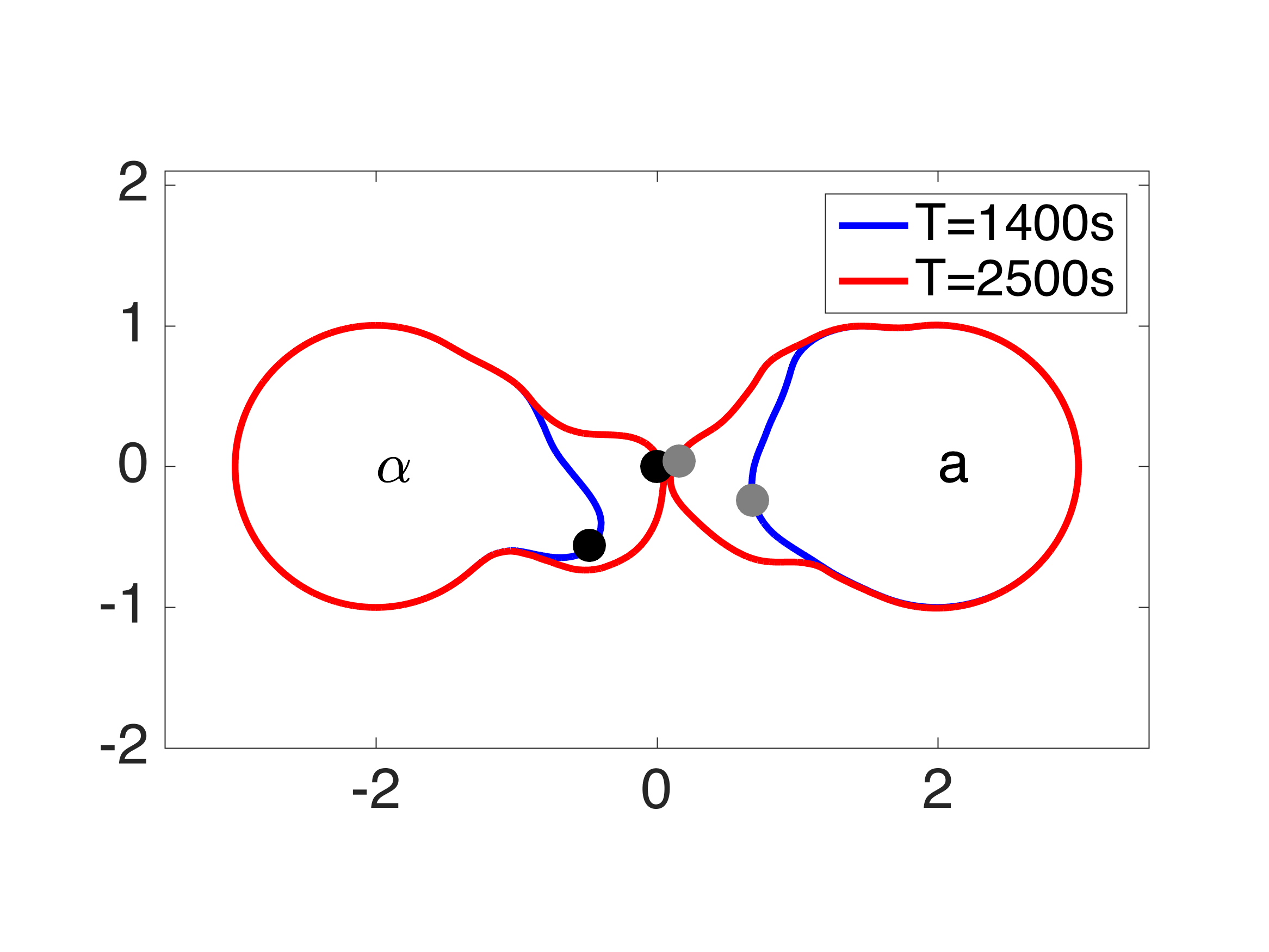


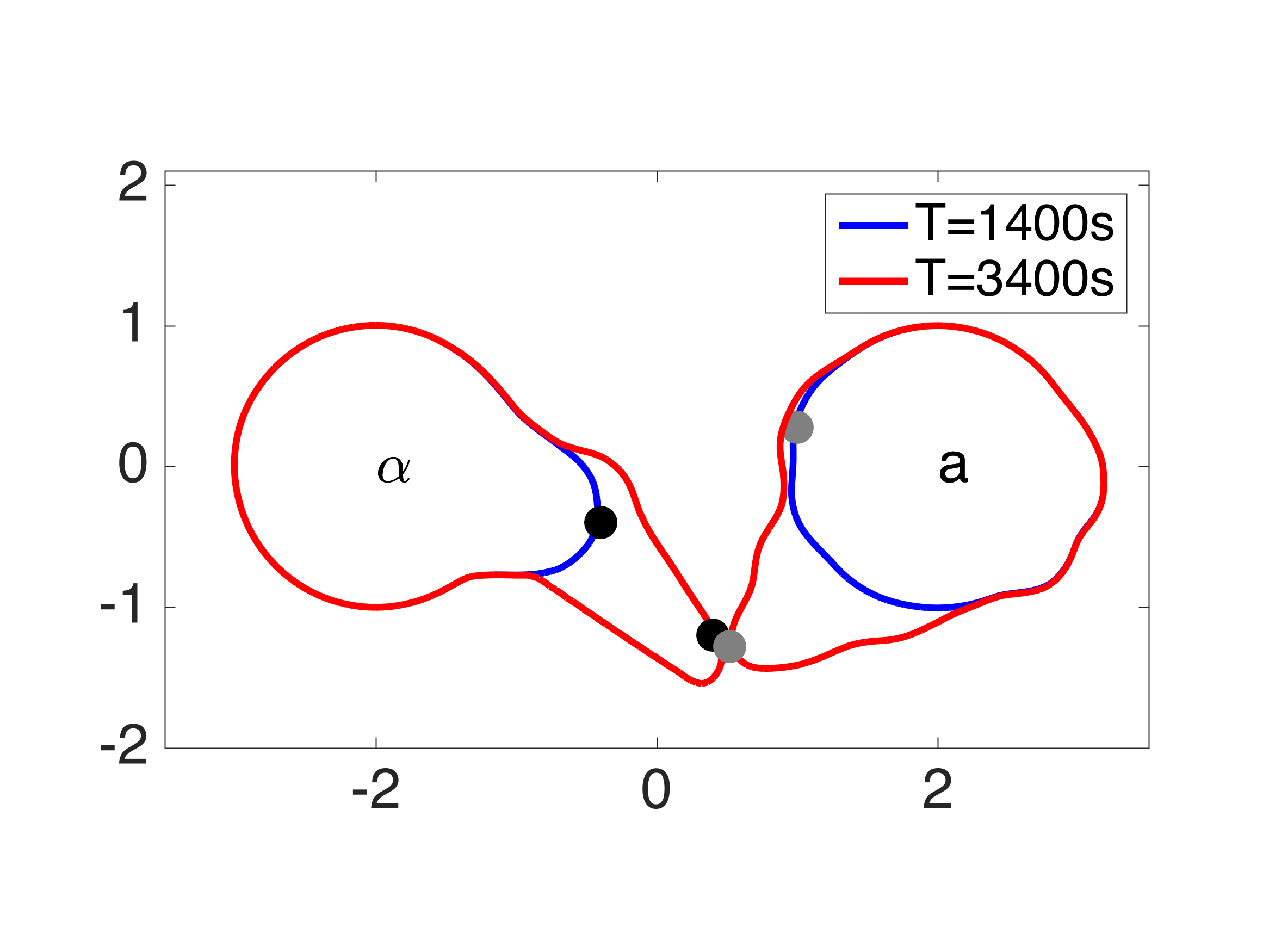

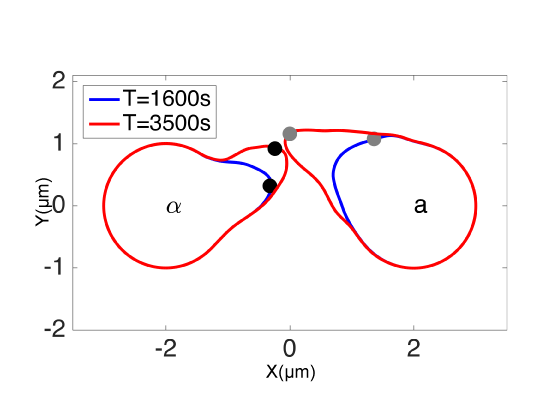


**Figure G.** Examples of two-cell mating under slow boundary velocity conditions. Filled circles represent positions of polarisomes.


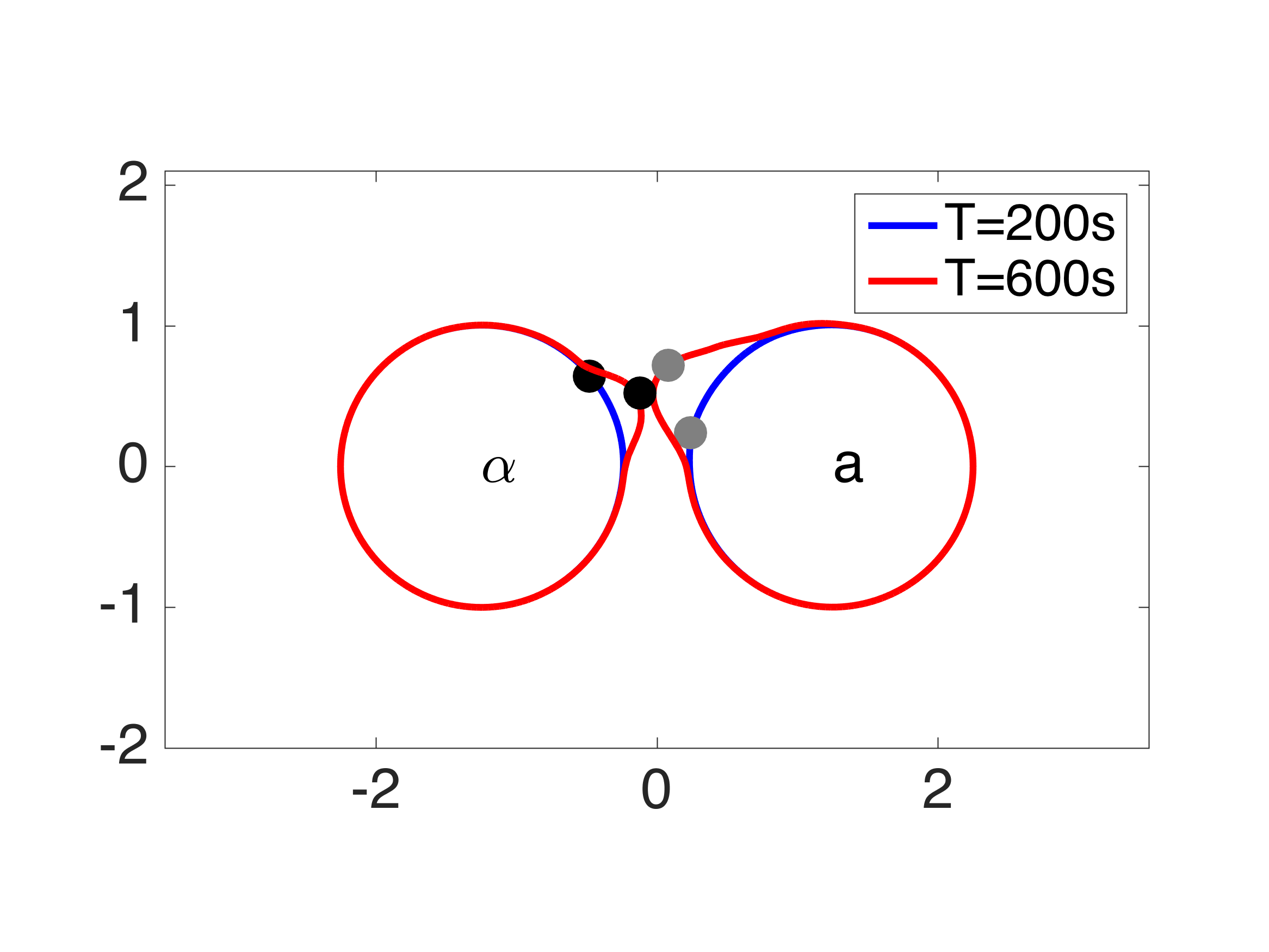

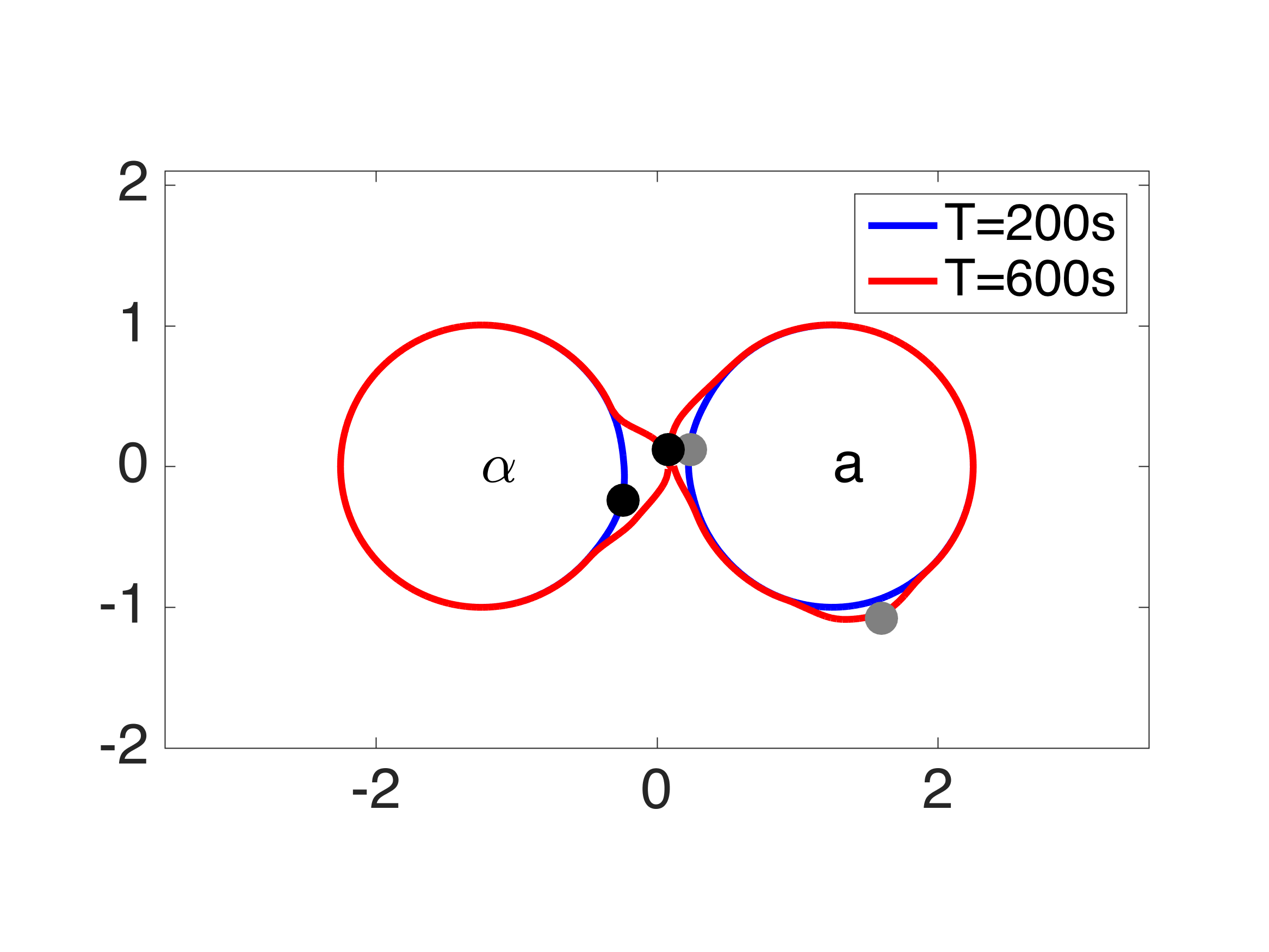


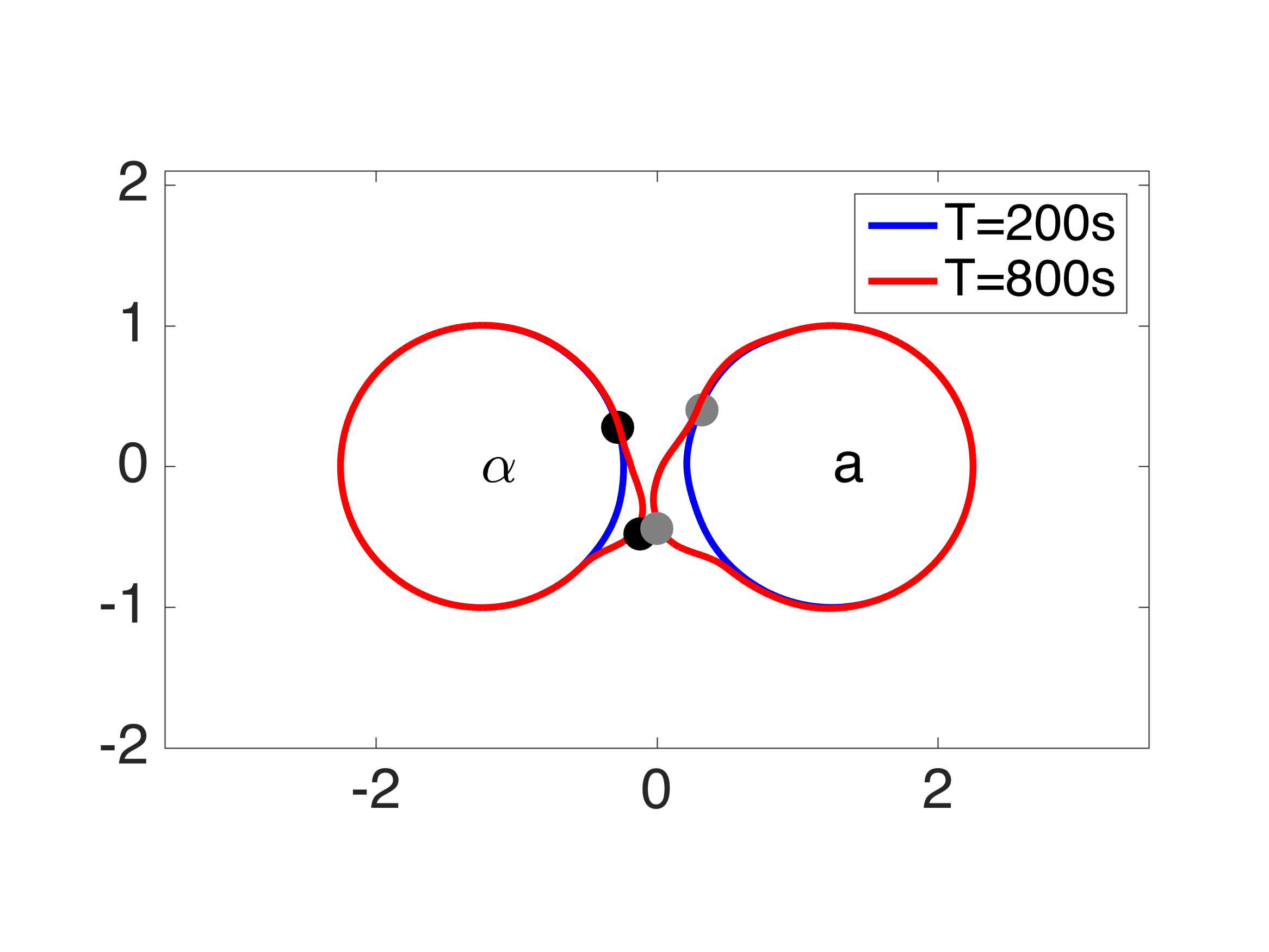

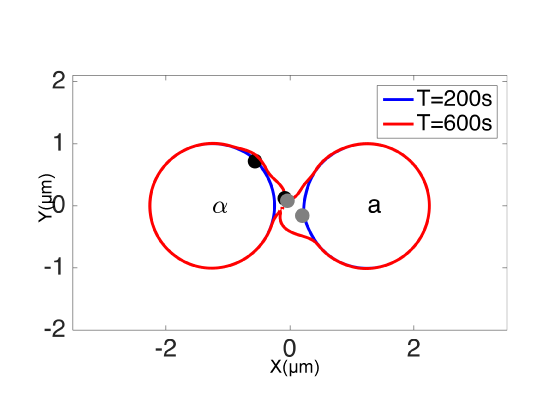


**Figure H.** Examples of two-cell mating when cell centers are 2.5 μm apart (close conditions). Filled circles represent positions of polarisomes.


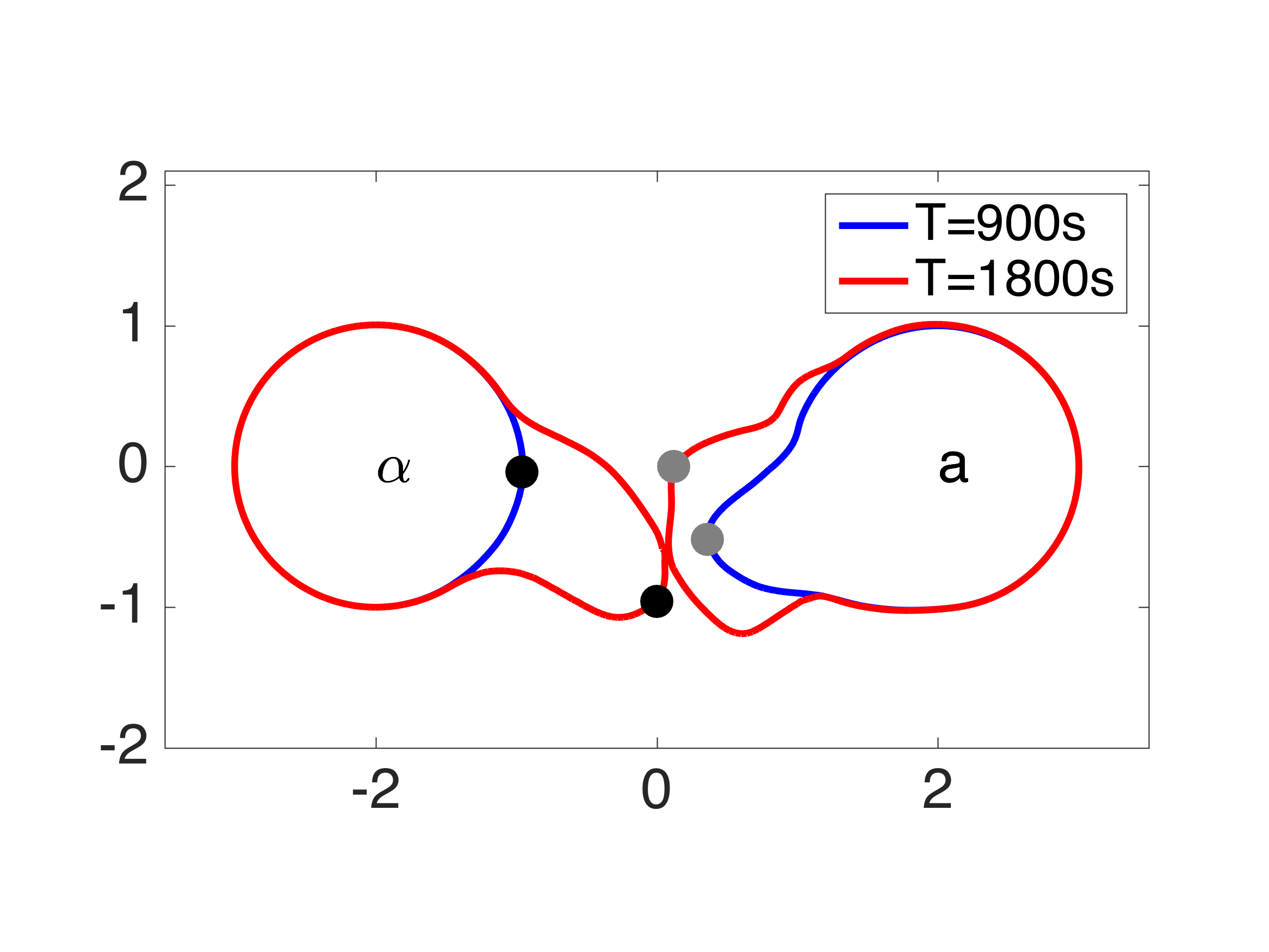

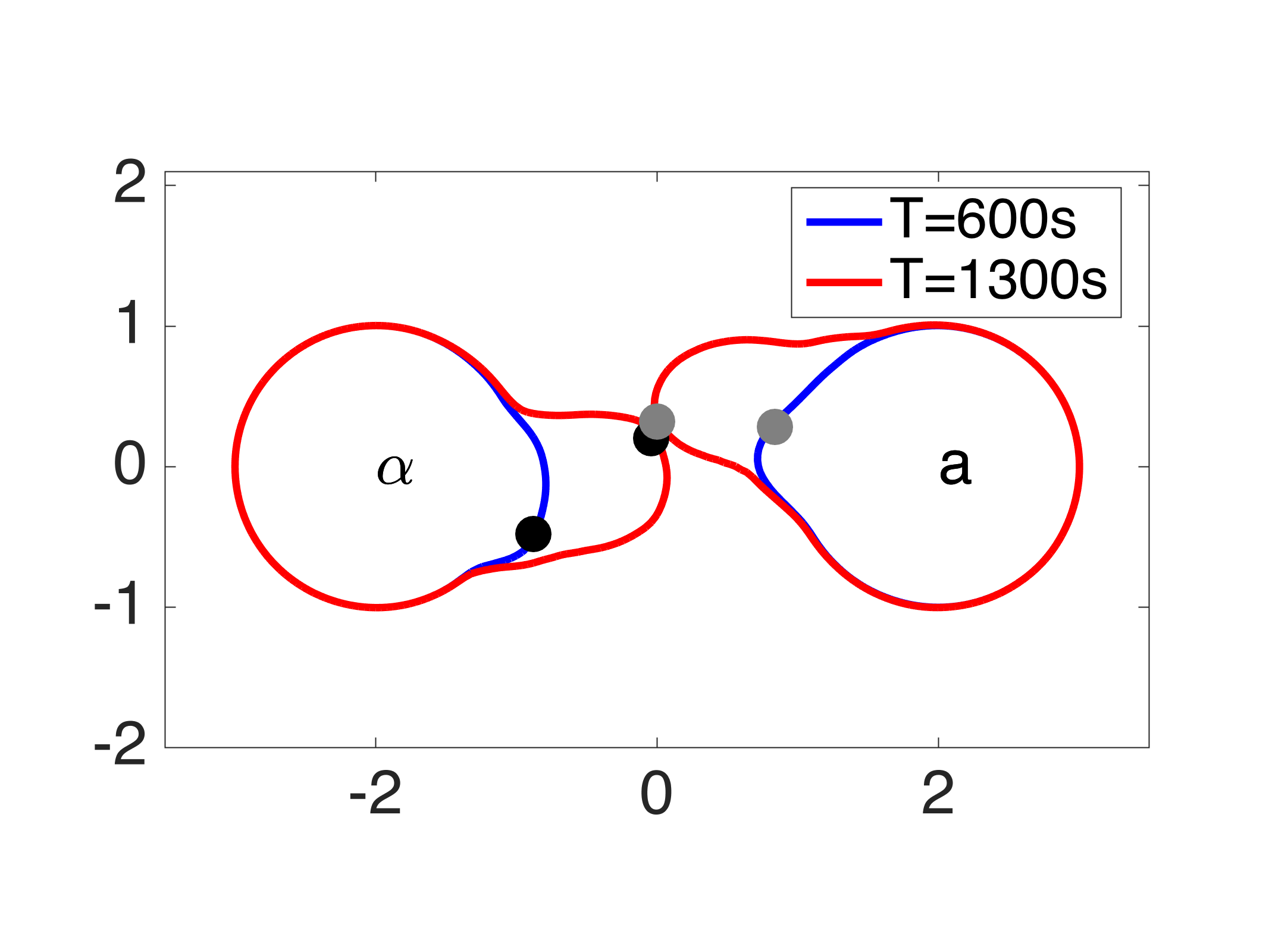


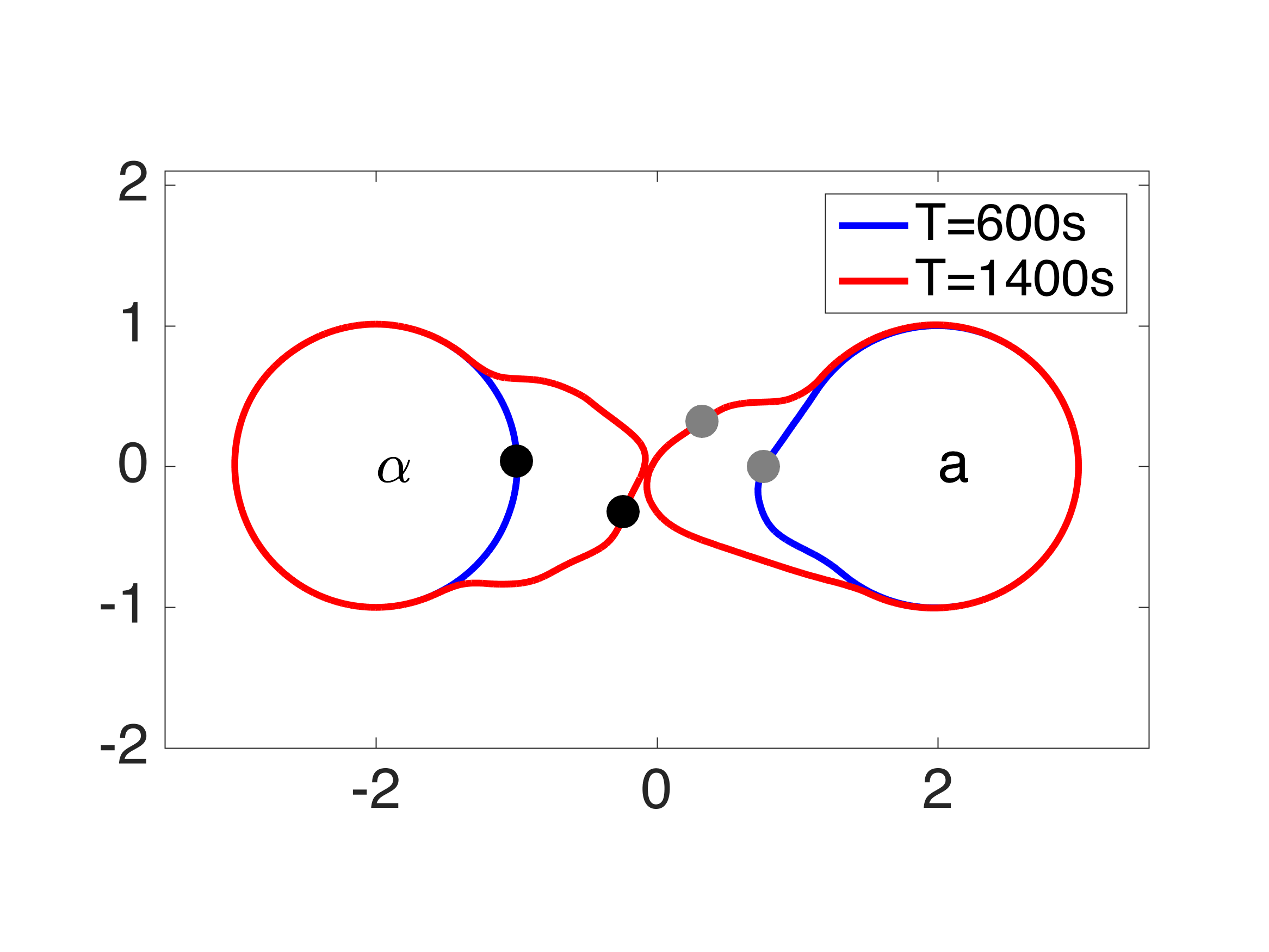

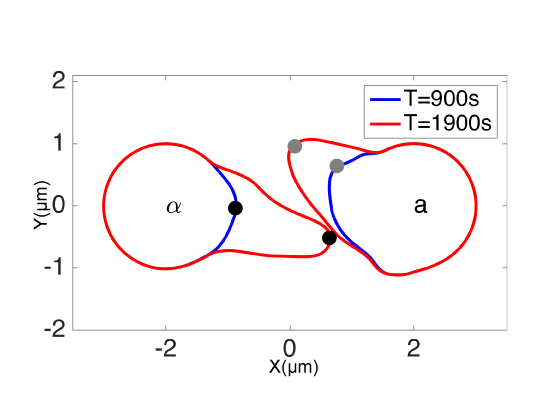


**Figure I.** Examples of two-cell mating with isotropic sources of pheromone. Filled circles represent positions of polarisomes.


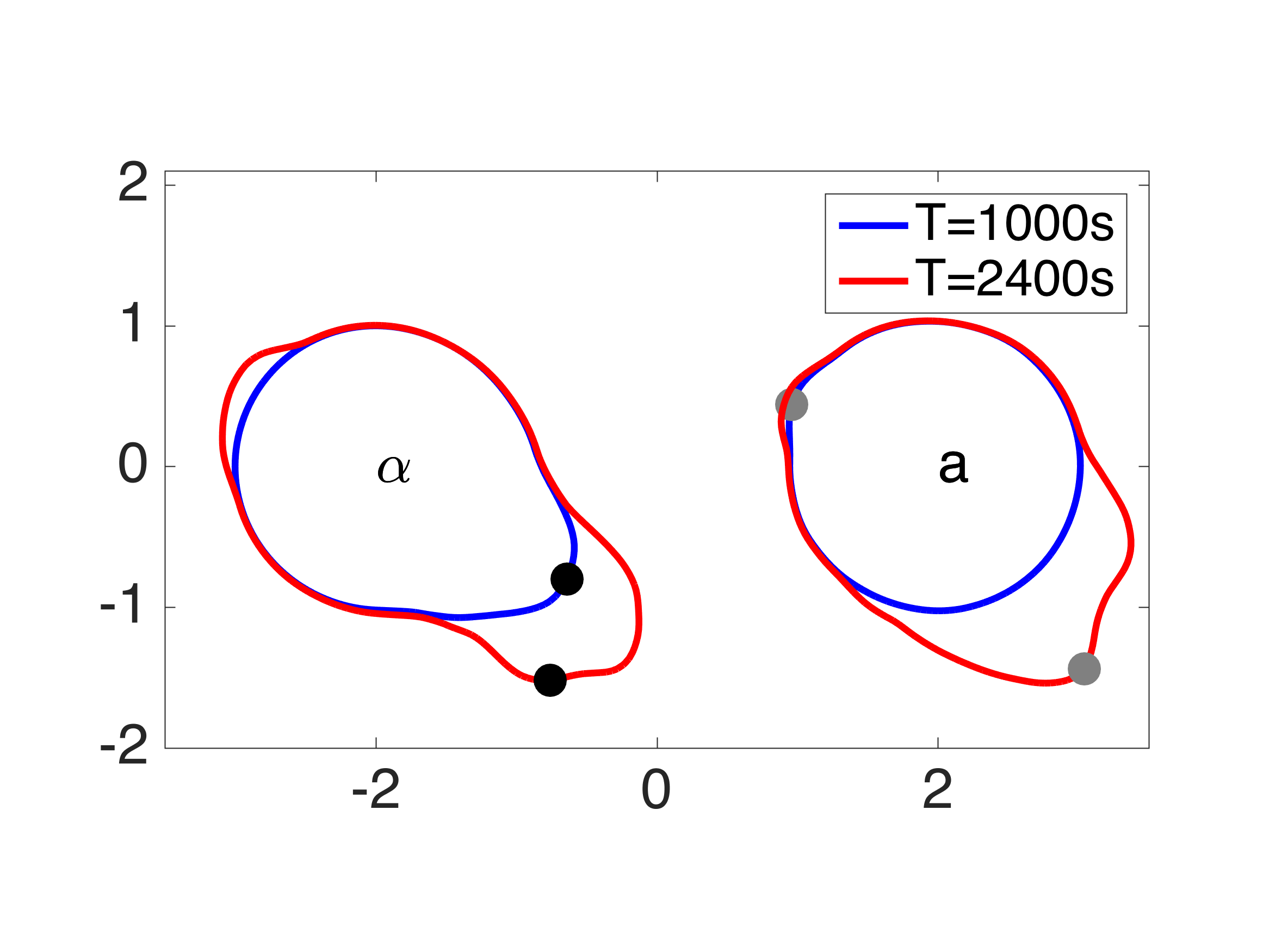

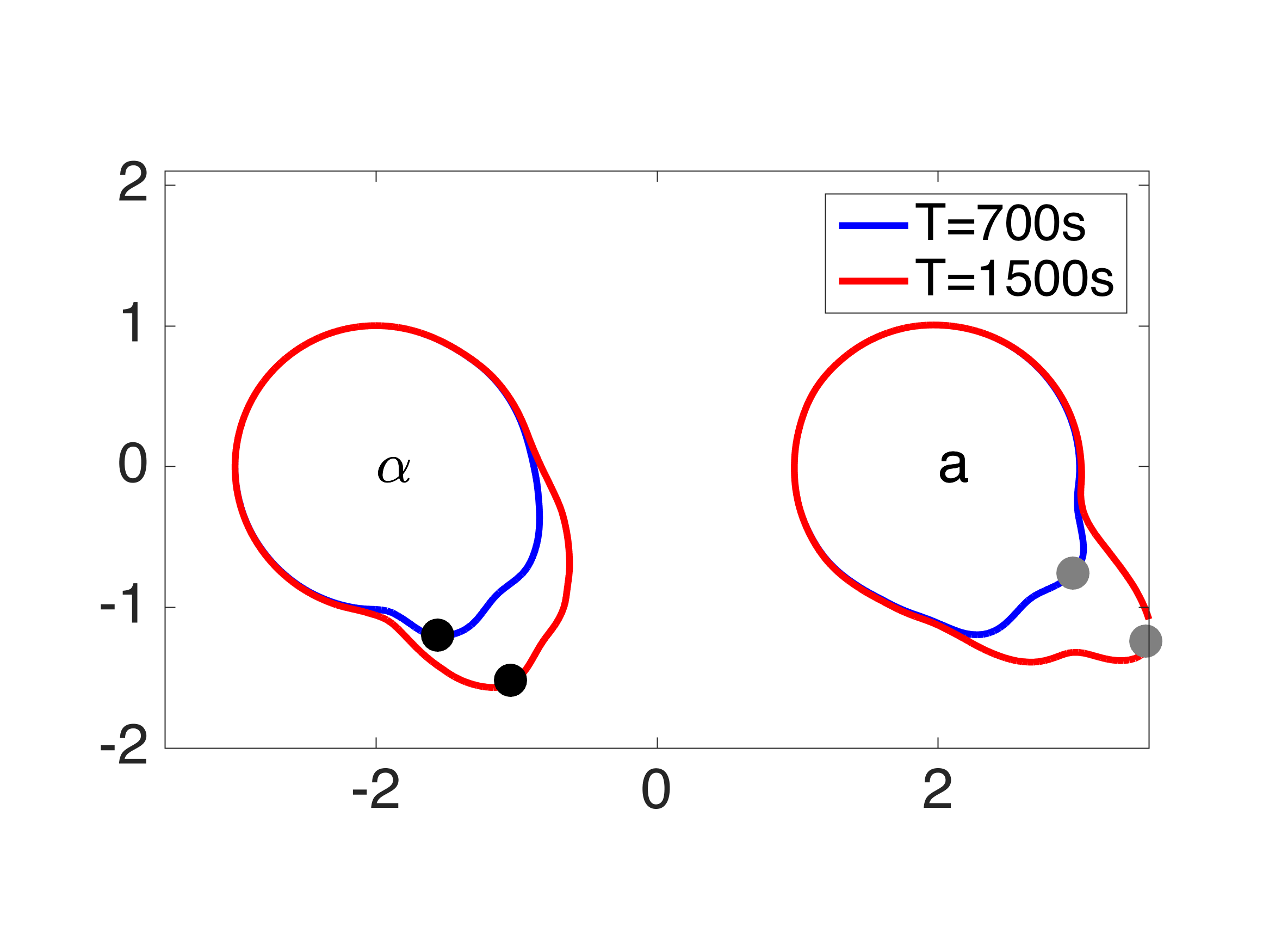


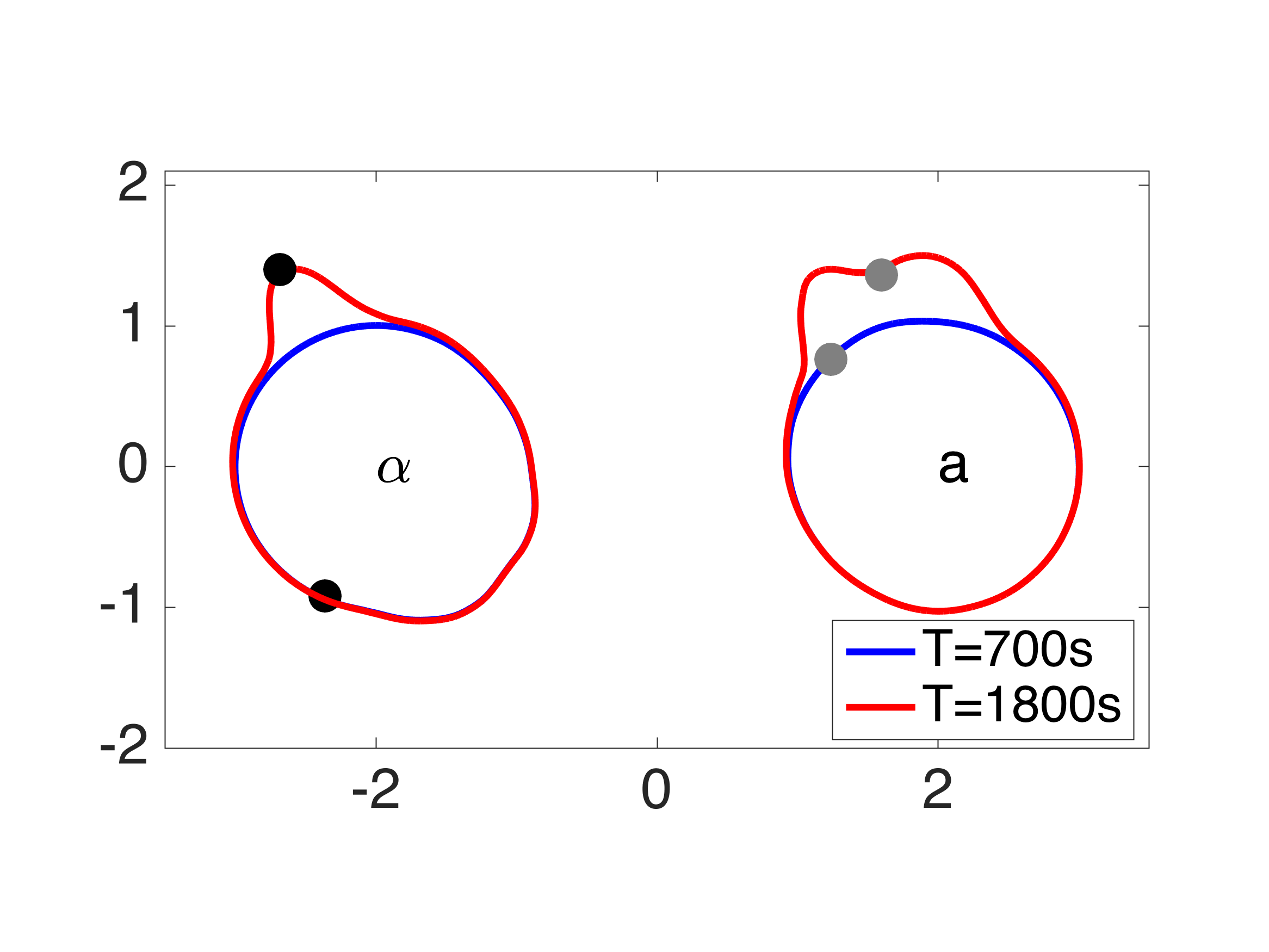

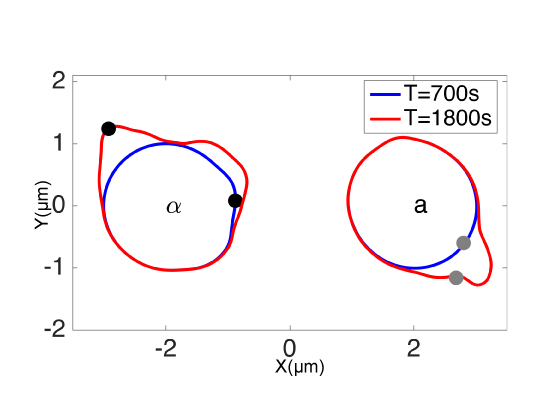


**Figure J.** Examples of two-cell mating with supersensitive cells. Filled circles represent positions of polarisomes.


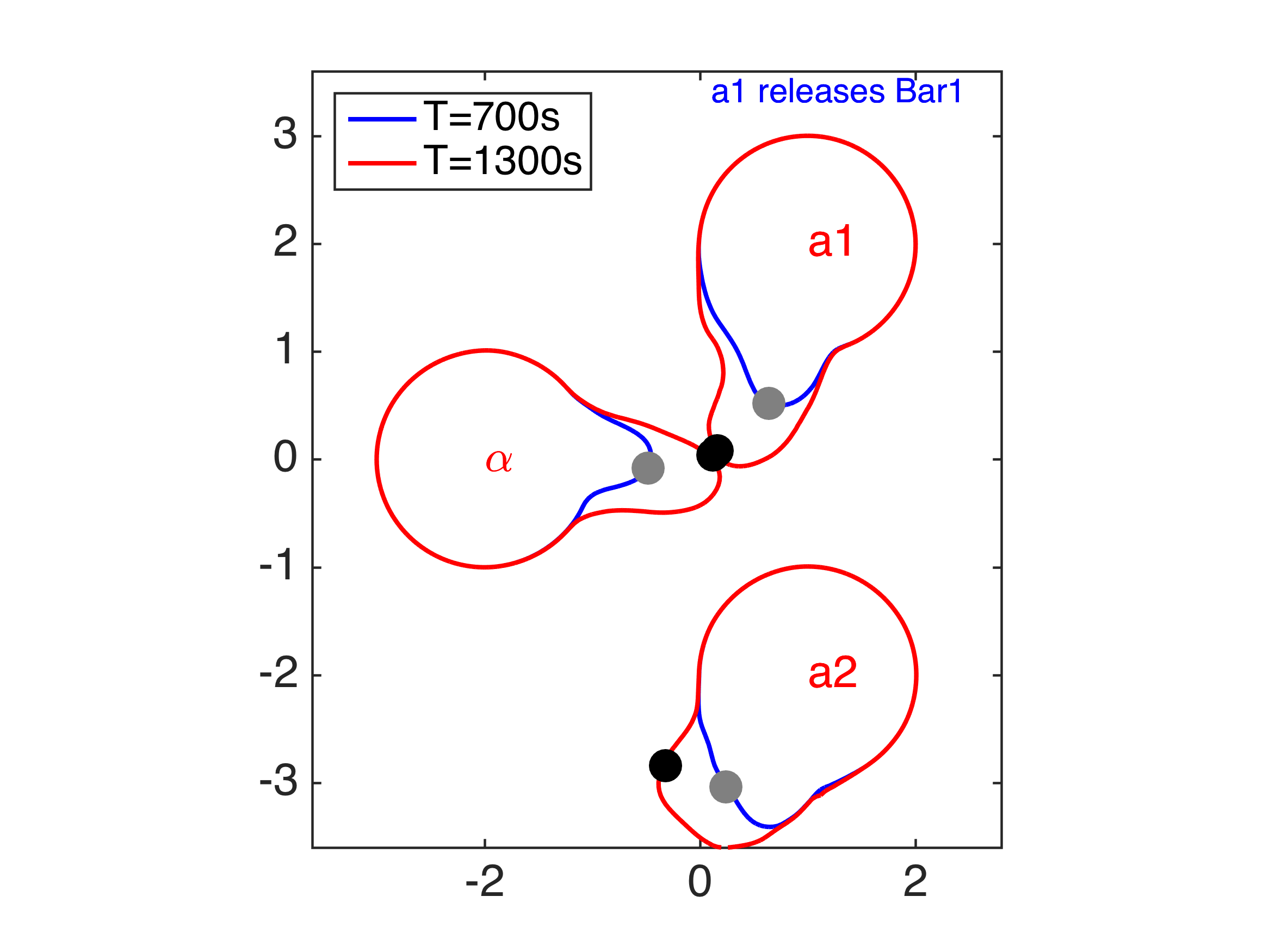

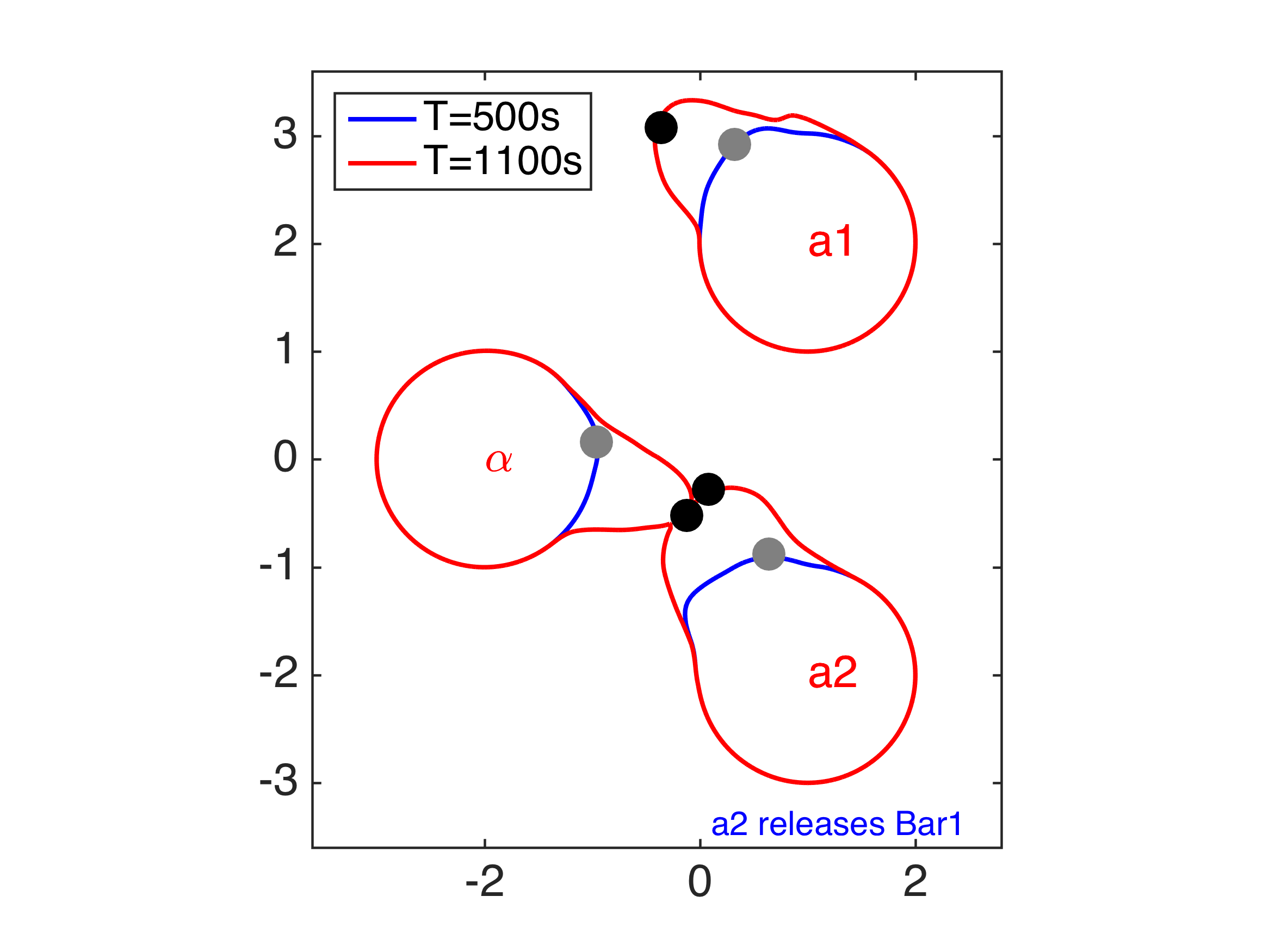


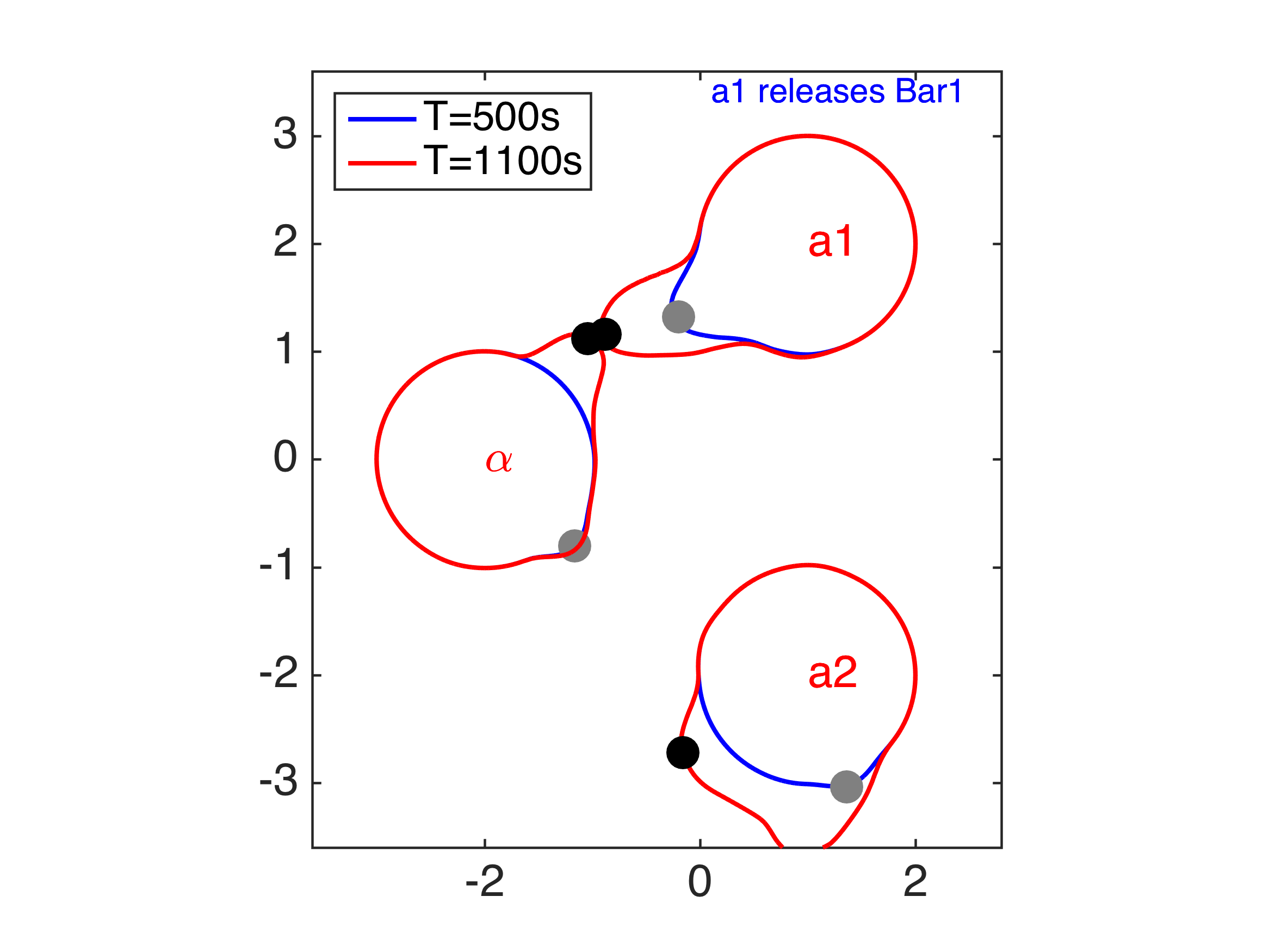

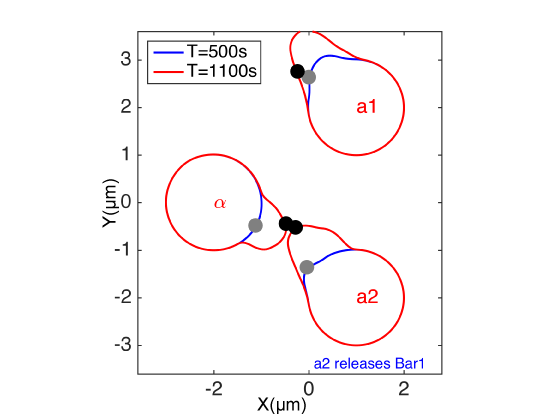


**Figure K.** Examples of three-cell mating competition with two **a**-cells (one Bar1+ and the other bar1Δ) competing for one α-cell. Filled circles represent positions of polarisomes.


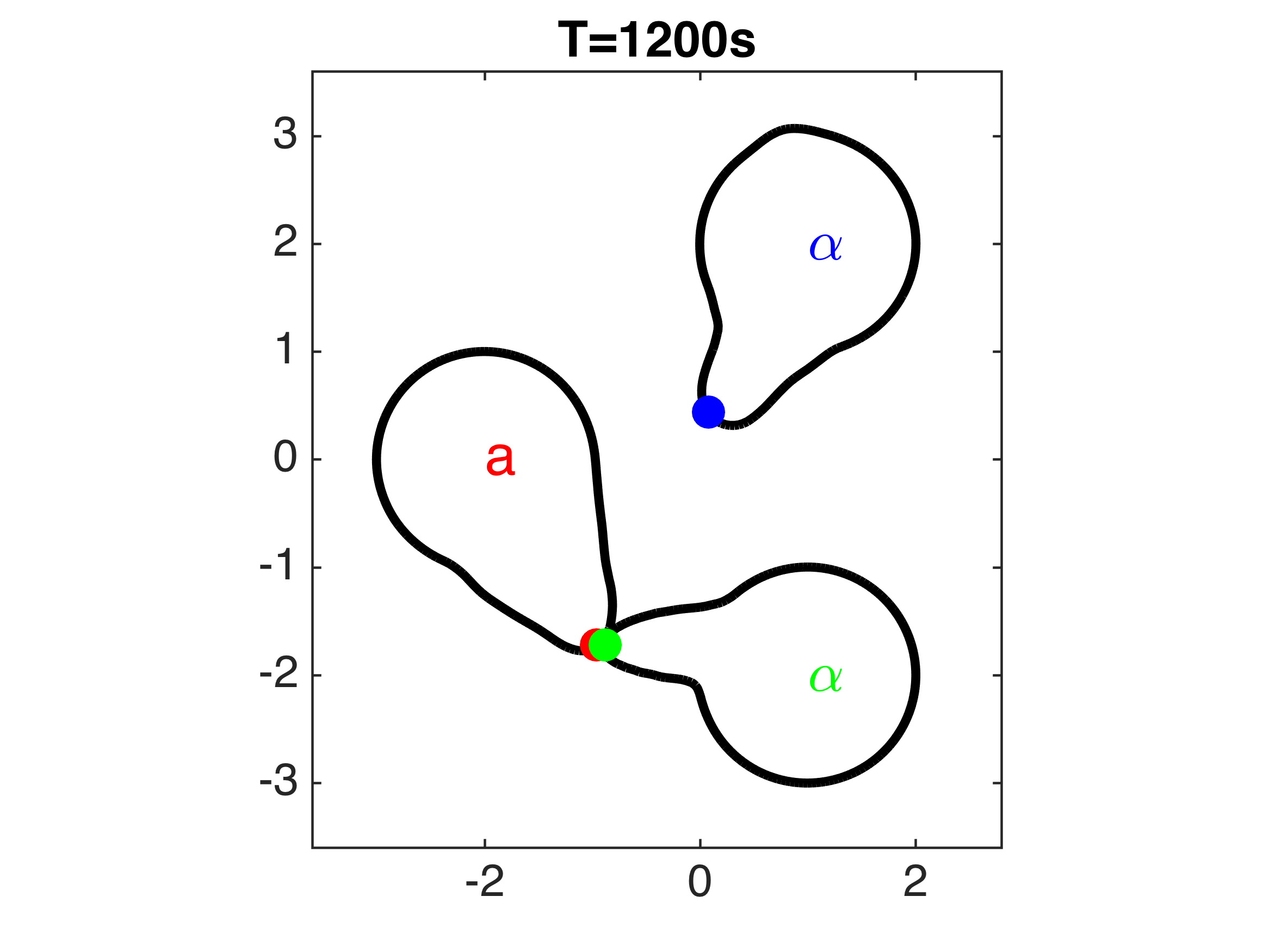

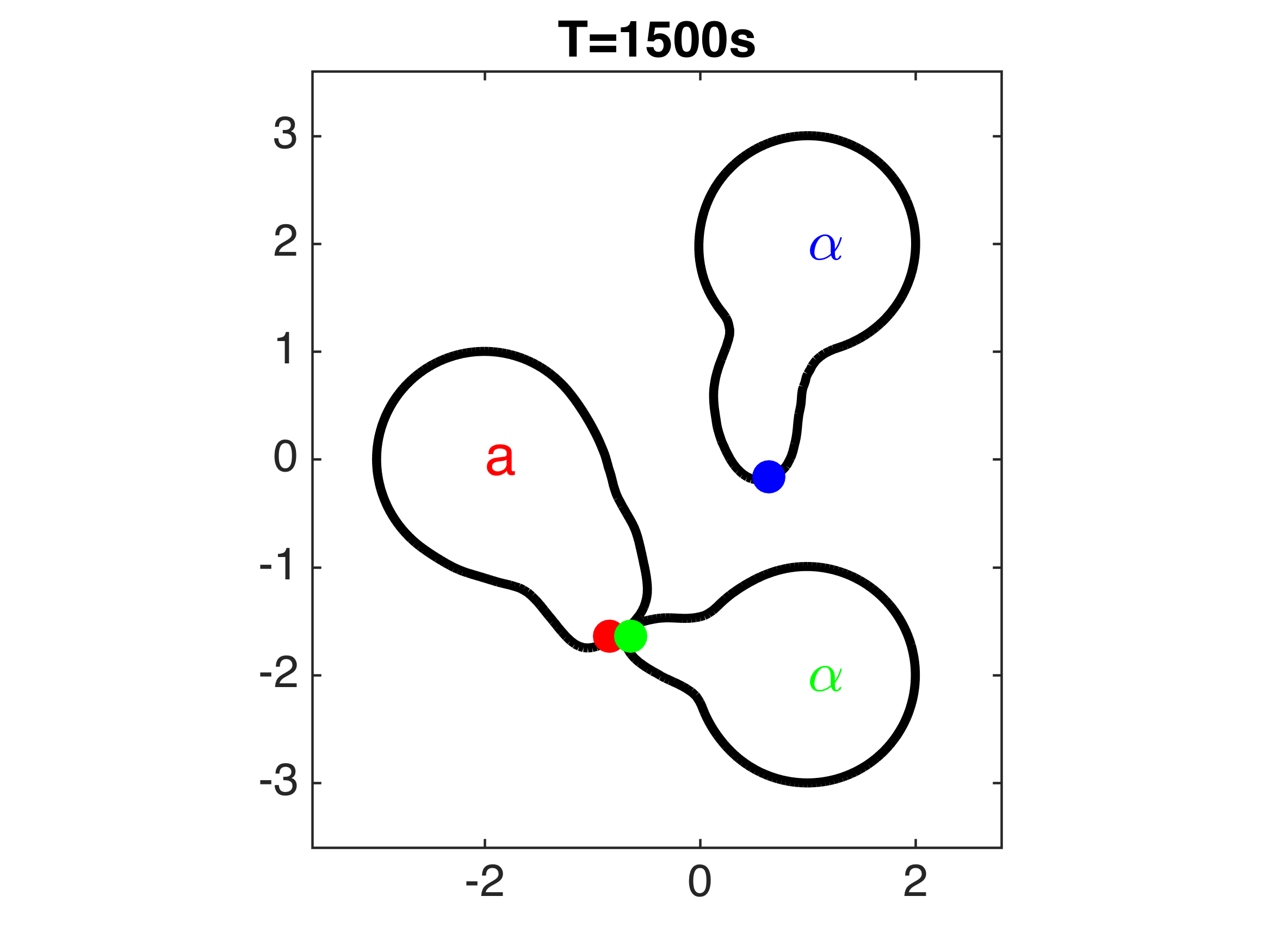


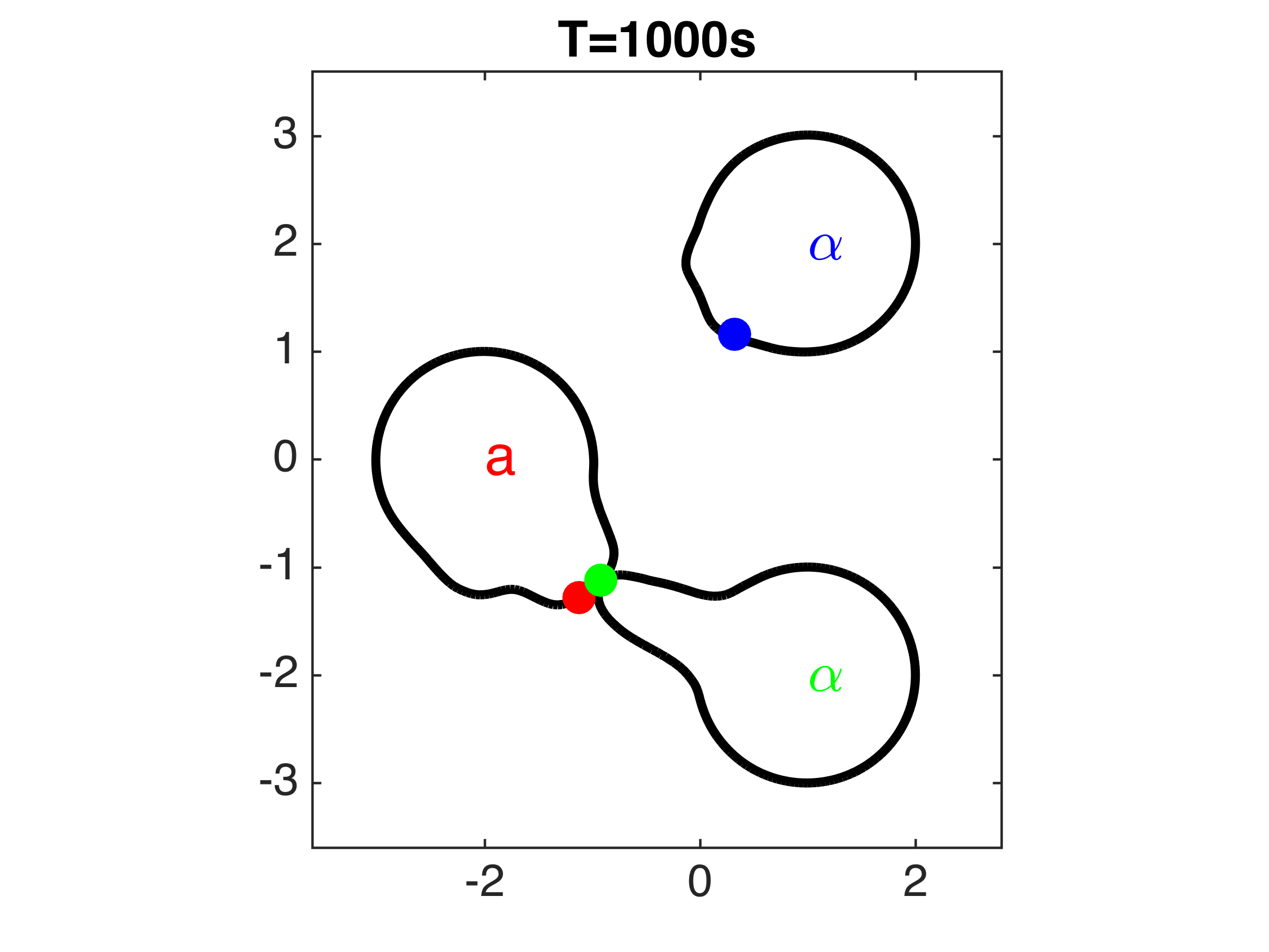

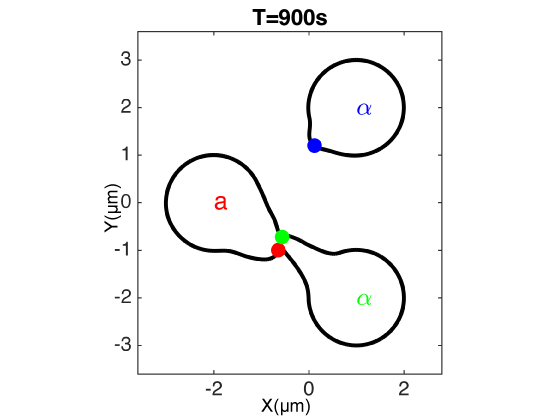


**Figure L.** Examples of three-cell mating discrimination with normal sensitivity cells, in which the α-cell in green secretes α-factor whereas the blue α-cell does not. Filled circles represent positions of polarisomes.

.


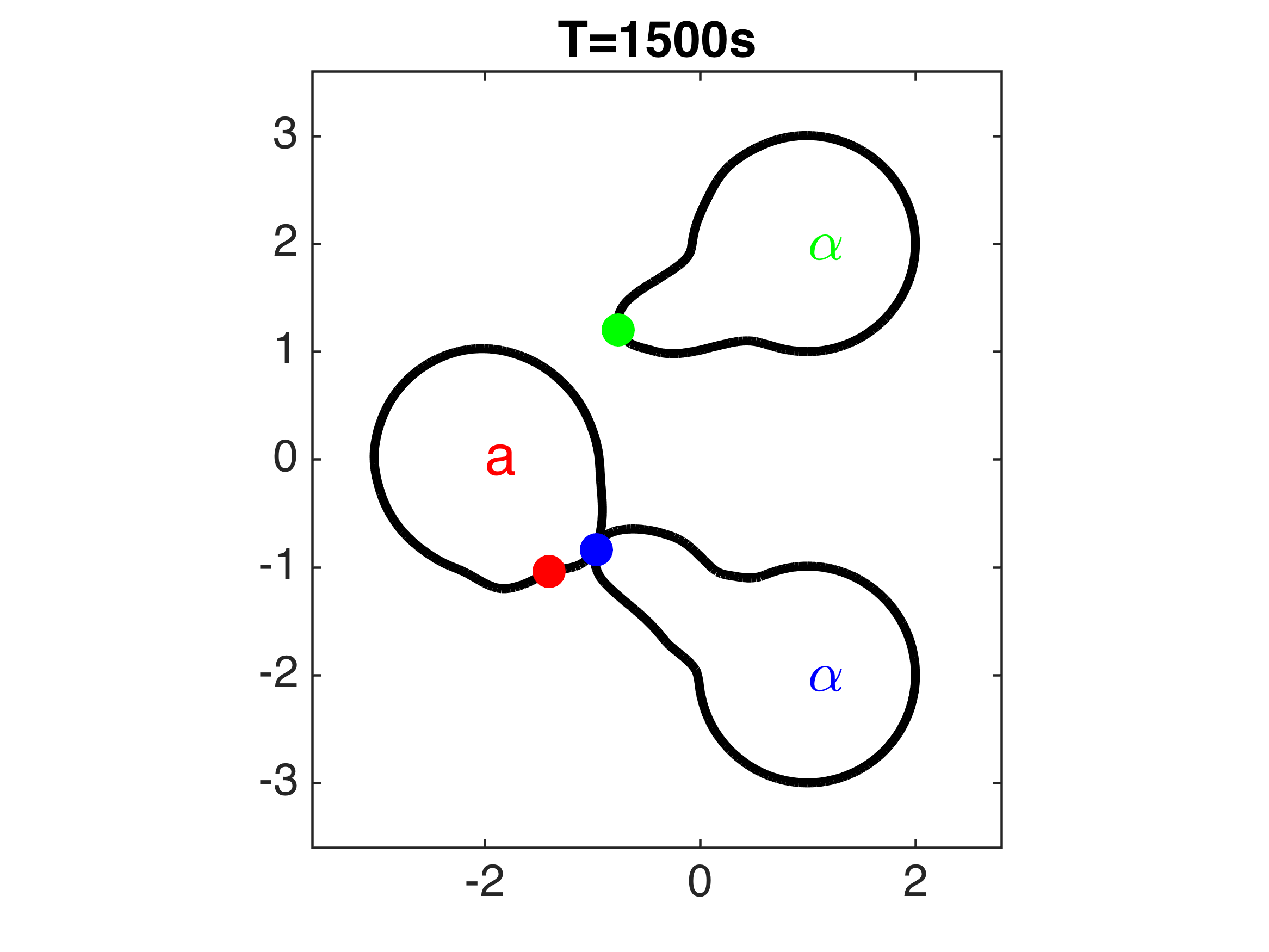

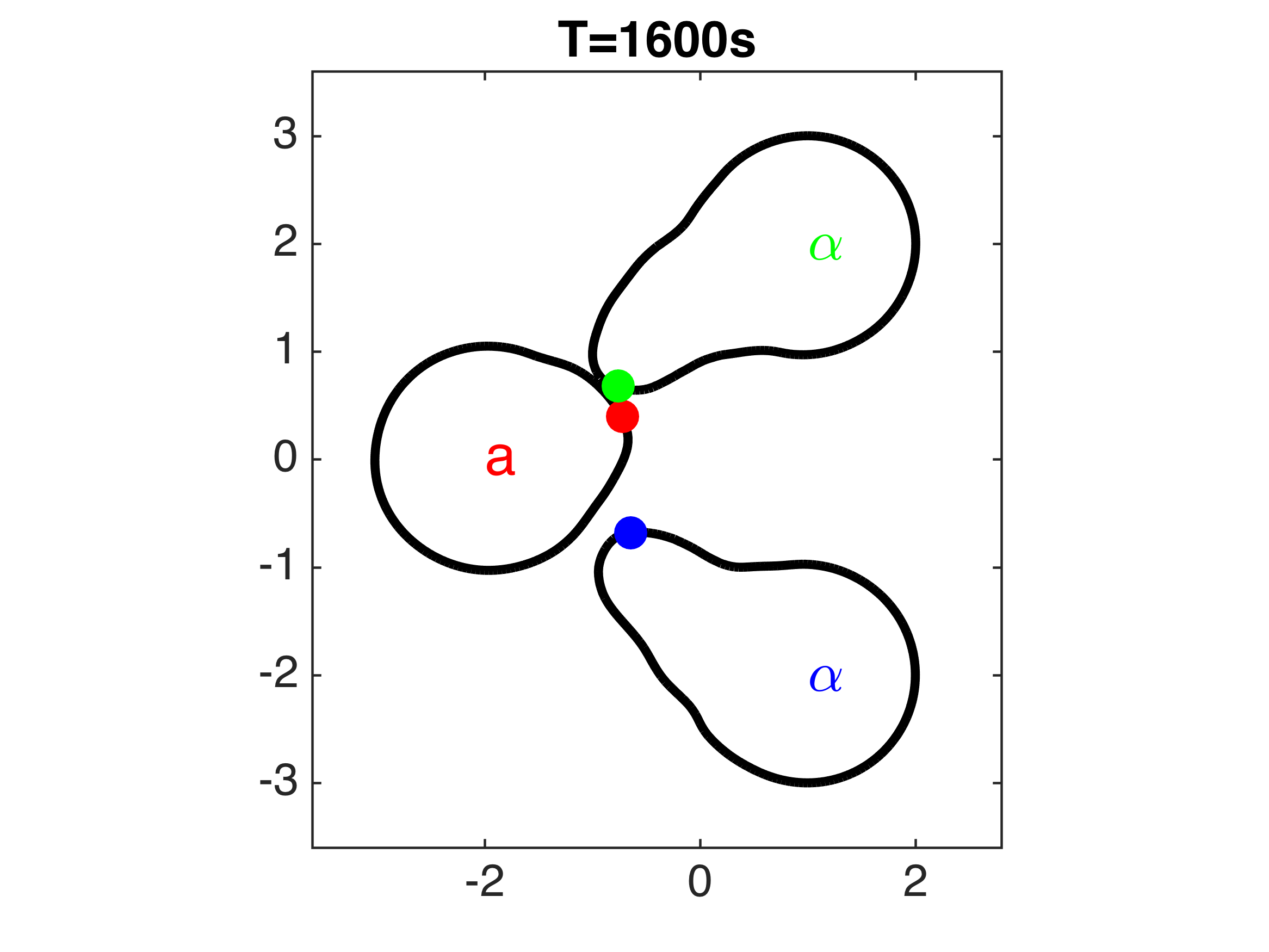


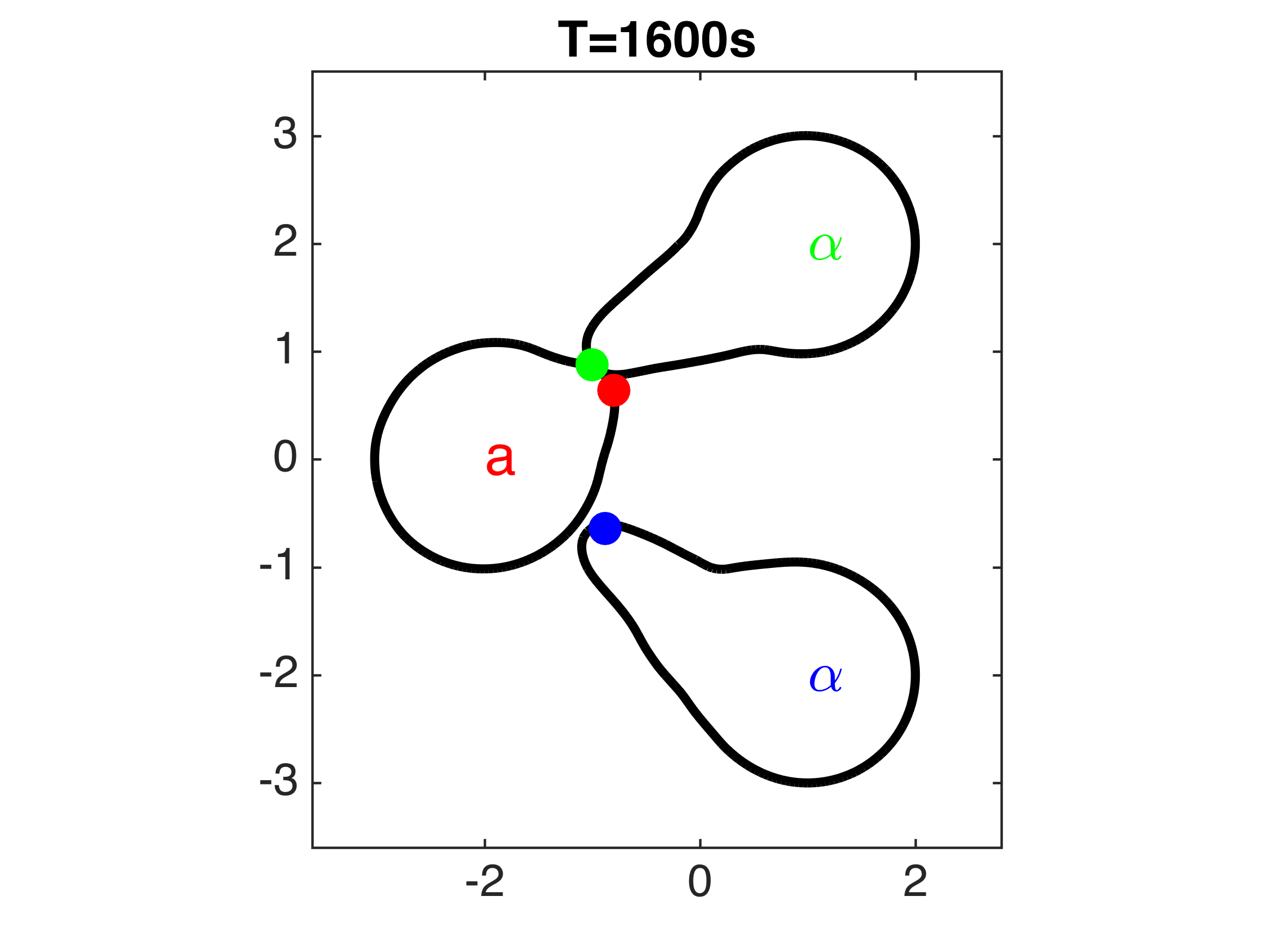

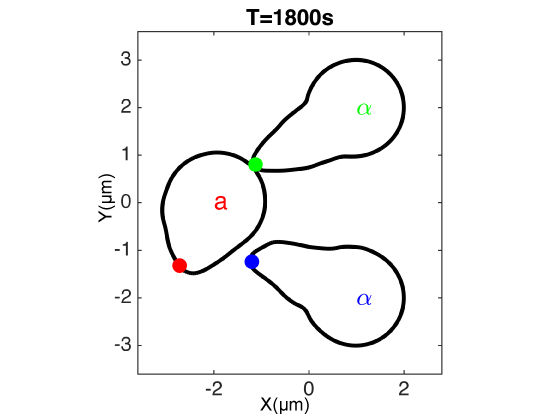


**Figure M.** Examples of three-cell mating discrimination with supersensitive cells in which the α-cell in green secrets α-factor whereas the blue α-cell does not. Filled circles represent positions of polarisomes.

**S7. Mating cell mixtures containing Bar1+ a-cells**

We imaged mating cell mixtures between Bar1+ **a**-cells and α-cells. Four shapshots are shown below (Fig. S14). In all of the examples, we observed short-range matings with center-to-center distances of less than 10 μm.

**
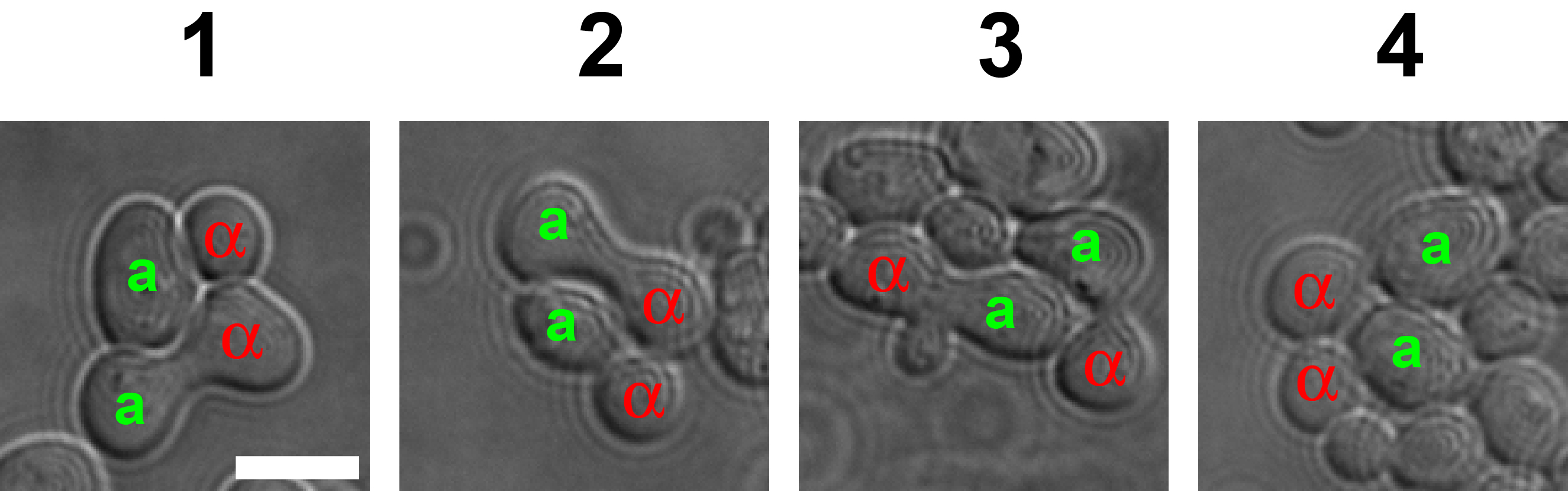
**

**Figure N.** Snapshots of mating between Bar1+ **a**-cells and α-cells. In each of the four pictures there are two pairs of mating cells. Scale bar = 5 μm.

**References**

1. Yi TM, Kitano H, Simon MI (2003) A quantitative characterization of the yeast heterotrimeric G protein cycle. Proceedings of the National Academy of Sciences of the United States of America 100: 10764-10769.

2. Chou CS, Bardwell L, Nie Q, Yi TM (2011) Noise filtering tradeoffs in spatial gradient sensing and cell polarization response. BMC Syst Biol 5: 196.

3. Sprague GF, Jr.; Thorner, J. W. (1992) Pheromone Response and Signal Transduction during the Mating Process of Saccharomyces cerevisiae. The Molecular and Cellular Biology of the Yeast Saccharomyces: Gene Expression. Cold Spring Harbor Monograph Archive. pp. 657-744.

4. Chou CS, Nie Q, Yi TM (2008) Modeling robustness tradeoffs in yeast cell polarization induced by spatial gradients. PLoS One 3: e3103.

5. Chou CS, Bardwell L, Nie Q, Yi TM (2011) Noise filtering tradeoffs in spatial gradient sensing and cell polarization response. Bmc Systems Biology 5.

6. Chou CS, Moore TI, Chang SD, Nie Q, Yi TM (2012) Signaling regulated endocytosis and exocytosis lead to mating pheromone concentration dependent morphologies in yeast. Febs Letters 586: 4208-4214.

7. Dorer R, Pryciak PM, Hartwell LH (1995) Saccharomyces cerevisiae cells execute a default pathway to select a mate in the absence of pheromone gradients. J Cell Biol 131: 845-861.

8. Peng DP, Merriman B, Osher S, Zhao HK, Kang MJ (1999) A PDE-based fast local level set method. Journal of Computational Physics 155: 410-438.

9. Adalsteinsson D, Sethian JA (1999) The fast construction of extension velocities in level set methods. Journal of Computational Physics 148: 2-22.

10. Osher S, Sethian JA (1988) Fronts Propagating with Curvature-Dependent Speed - Algorithms Based on Hamilton-Jacobi Formulations. Journal of Computational Physics 79: 12-49.
